# Biparental incubation-scheduling: no experimental evidence for major energetic constraints

**DOI:** 10.1093/beheco/aru156

**Published:** 2014-09-03

**Authors:** Martin Bulla, Will Cresswell, Anne L. Rutten, Mihai Valcu, Bart Kempenaers

**Affiliations:** ^a^Department of Behavioural Ecology and Evolutionary Genetics, Max Planck Institute for Ornithology, Eberhard Gwinner Str. 7, 82319 Seewiesen, Germany and; ^b^School of Biology, University of St. Andrews, St. Andrews, Harold Mitchell Building, Fife KY16 9TH, UK

**Keywords:** Arctic, biparental incubation, *Calidris pusilla*, constancy, cross-over design, energetic constraints, energetic demands, incubation bout length, replication, scheduling, semipalmated sandpiper, shorebird, statistical power.

## Abstract

Incubation is energetically demanding, but it is debated whether these demands constrain incubation-scheduling (i.e., the length, constancy, and timing of incubation bouts) in cases where both parents incubate. Using 2 methods, we experimentally reduced the energetic demands of incubation in the semipalmated sandpiper, a biparental shorebird breeding in the harsh conditions of the high Arctic. First, we decreased the demands of incubation for 1 parent only by exchanging 1 of the 4 eggs for an artificial egg that heated up when the focal bird incubated. Second, we reanalyzed the data from the only published experimental study that has explicitly tested energetic constraints on incubation-scheduling in a biparentally incubating species (Cresswell et al. 2003). In this experiment, the energetic demands of incubation were decreased for both parents by insulating the nest cup. We expected that the treated birds, in both experiments, would change the length of their incubation bouts, if biparental incubation-scheduling is energetically constrained. However, we found no evidence that heating or insulation of the nest affected the length of incubation bouts: the combined effect of both experiments was an increase in bout length of 3.6min (95% CI: −33 to 40), which is equivalent to a 0.5% increase in the length of the average incubation bout. These results demonstrate that the observed biparental incubation-scheduling in semipalmated sandpipers is not primarily driven by energetic constraints and therefore by the state of the incubating bird, implying that we still do not understand the factors driving biparental incubation-scheduling.

## Lay Summary

It is believed that a bird’s energetic reserves determine when and for how long it incubates its eggs. We challenge this view for species where both parents incubate. We experimentally reduced the energetic demands of incubation by heating and insulating the nest. These treatments had no major effect on the length of incubation bouts.

## INTRODUCTION

Avian incubation is energetically demanding (e.g., Vleck 1981; reviewed by Williams 1996; and by Tinbergen and Williams 2002; Piersma et al. 2003), mainly because incubating parents trade-off their energetic needs with thermal needs of their developing embryos (i.e., trade-off between foraging and incubation; reviewed by Reid et al. 2002; and by Tinbergen and Williams 2002). Thus, energetic demands of incubation should constrain incubation-scheduling, that is, the length of incubation bouts, their constancy (the amount of time birds actually incubate within an incubation bout), and their timing.

Such energetic constraints on incubation-scheduling are expected, reported, and experimentally confirmed for unassisted, uniparental incubation (e.g., Aldrich and Raveling 1983; Bryan and Bryant 1999; Reid et al. 1999; Cresswell et al. 2004; Ardia et al. 2009) and for extreme events where biparental incubation (temporarily) turns into uniparental incubation, for example, when an off-nest bird delays its return to the nest (Davis 1982; Chaurand and Weimerskirch 1994; Weimerskirch 1995; Gauthier-Clerc et al. 2001). In these cases, there appears to be a body-mass threshold, below which birds interrupt their incubation and leave the nest (Aldrich and Raveling 1983; Chaurand and Weimerskirch 1994; Weimerskirch 1995; Gauthier-Clerc et al. 2001).

In contrast, during (regular) biparental incubation, the energetic constraints on incubation-scheduling are expected to be reduced (Williams 1996; Tinbergen and Williams 2002). Here, parents may always have enough time to replenish their energy reserves, which became depleted during their previous incubation session. The 2 experimental studies addressing this issue in biparentally incubating birds yielded contradictory results (Cresswell et al. 2003; Kosztolanyi et al. 2009). The first study—conducted in the high Arctic on semipalmated sandpipers, *Calidris pusilla*—reported that an experimental reduction in the energetic demands of incubation (achieved with a polystyrene insulation of the nest cup) prolonged incubation bouts and concluded that biparental incubation-scheduling is energetically constrained (Cresswell et al. 2003). To date this is the only experiment that explicitly tested the hypothesis that energetic constraints affect biparental incubation-scheduling. However, here we argue that the conclusion of this study needs to be revised because the data and analyses were inconsistent with the experimental design (detailed in Supplementary 1: Section 1, Reanalysis of the Insulation Experiment). The second study—conducted in a hot, arid environment on Kentish plovers, *Charadrius alexandrinus*—reported that an experimental increase in the energetic demands of incubation (by artificial cooling of the nest during the night) increased constancy of incubation, suggesting that biparental incubation-scheduling is not energetically constrained (Kosztolanyi et al. 2009). However, the night conditions in this arid environment (with ground temperatures around 25 °C) are within the thermo-neutral zone of small shorebird species (Kersten and Piersma 1986), and thus should not be energetically stressful to Kentish plovers. Hence, one could argue that the energetic demands of heating the eggs were negligible under these conditions (Tinbergen and Williams 2002), implying that the cooling treatment might have been ineffective in manipulating the energy reserves of the Kentish plover parents. In sum, it still remains unclear whether energetic demands of incubation constrain incubation-scheduling in biparentally incubating species.

The aim of this study is to resolve whether energetic demands of incubation are a major factor driving incubation-scheduling (i.e., the division of parental care) in biparental semipalmated sandpipers breeding in harsh, energetically stressful conditions in the high Arctic. Using a new experiment and a more rigorous and appropriate analysis of the previous experiment (Cresswell et al. 2003), we tested whether reduced energetic demands of incubation changed incubation-scheduling. Both experiments were carried out at the same site, under similar environmental conditions (Supplementary 1: Section 2, Seasonal Differences), but were separated by 12 years. Although the previous experiment reduced energetic demands of incubation for both parents, in the new experiment, we reduced energetic demands of incubation for 1 parent only using an artificial egg that provided heat when the focal bird incubated (heating experiment). The expected effect of this manipulation on incubation-scheduling depends on which individual determines incubation bout length. If the level of energy reserves of the incubating parent determines incubation bout length (as implied by Cresswell et al. 2003), then the treated birds should prolong their incubation bouts or increase their incubation constancy because their energetic reserves will last longer when demands are reduced. Alternatively, if the energy reserves of the off-nest parent determine incubation bout length, then the off-nest bout of the treated bird (which is the incubation bout of the untreated bird) should decrease in length. This is because the off-nest bird, which was treated during its previous incubation bout, will have a smaller energy deficit to recover, and thus could return to the nest earlier. Both scenarios could potentially also reduce the number or length of exchange gaps (time period during which eggs are left unattended as part of the exchange process at the nest), but our previous work shows that exchanges are usually immediate (Bulla et al. 2014a). We further investigated in how far the results of the 2 experiments are comparable given potential differences in the strength of the treatment: heating in the new experiment and insulating in the first experiment. To this end, we used artificial nest scrapes, and approximated the amount of energy an artificial brood-patch consumed when a nest scrape either contained a heating egg (heating experiment) or was insulated (insulation experiment).

## MATERIALS AND METHODS

### Heating experiment

#### Study area and species 

We studied a population of semipalmated sandpipers near Barrow, Alaska (71.32° N, 156.65° W), between 1 June and 21 July 2012. The study area and species are already described in detail elsewhere (Ashkenazie and Safriel 1979; Bulla et al. 2014a). Barrow has continuous daylight throughout the breeding season, but environmental conditions show consistent and substantial diel fluctuations; ambient temperatures are generally low, below 5 °C, but surface tundra temperatures can reach up to 28 °C (Supplementary Figure S1 in Bulla et al. 2014a). Previous work on the same study area found that incubation lasted on average for 21 days, with an average bout length of 11.5h; incubation bout length increased over the incubation period and was ca. 50min longer in females than in males; females had a slightly higher incubation constancy, and a higher overall probability to incubate during the colder period of the day (Bulla et al. 2014a).

#### Sampling of individuals and monitoring of incubation behavior

Capture, marking, measuring, and blood sampling of individuals (for sexing), as well as the general procedures to monitor incubation behavior are described in detail elsewhere (Bulla et al. 2014a). Briefly, incubation was determined by a high-resolution MSR® external temperature-probe placed in the middle of a nest between the 4 eggs and connected to a MSR® 145 data logger (MSR® Electronics GmbH, http://www.msr.ch/en/); this logger also recorded the tundra surface temperature outside of the nest. Both temperatures were logged every 5 s throughout the incubation period. Constant incubation-temperatures higher than tundra temperatures were interpreted as continuous incubation; the start of incubation was determined from a steep increase, the interruption of incubation from a steep decrease in nest temperatures (Supplementary Figure S4 in Bulla et al. 2014a).

A radio frequency identification device (RFID)—a thin antennae loop fitted into a nest cup and connected to a data logger—registered every 5 s the identity of an incubating bird (marked with a green flag with embedded passive-integrated transponder; details in Bulla et al. 2014a). Thus, the temperature-based determination of incubation was overlaid with the RFID data, which allowed assigning each incubation bout to a parent (Supplementary Figure S4 in Bulla et al. 2014a).

The length of each incubation bout was extracted as the total time allocated to a single parent (i.e., the time between the arrival of a parent and its departure from the nest followed by incubation by its partner). The exchange-gap duration (the time between the departure of 1 parent and the return of the other) was excluded from the length of the incubation bout. The constancy of incubation was calculated from the temperature-based incubation data as the percentage of time a bird actually incubated within a given incubation bout (i.e., sat tightly on the eggs as opposed to egg rolling, nest maintenance or being off the nest).

In addition to previously described procedures, in this study, we protected control and treatment nests against avian predators using enclosures made of mesh wire (Supplementary 1: Section 3 Pictures, Picture S1).

#### Heating treatment

We reduced the energetic demands of incubation by exchanging 1 egg for an artificial heat-producing egg (29.4×21.0mm; Jan Petrů, http://www.forestspy.com). This egg was made of a heating element (with resistance 13.7–13.9 Ω and heating power of 10.5W; Jan Petrů, http://www.forestspy.com), a 1-wire digital thermometer (0.5 °C accuracy; model DS18S20, Maxim Integrated Products, http://www.maximintegrated.com), and a highly conductive “liquid metal” (70% mixture of powder aluminum—with particles 65 µm in diameter—and high-temperature epoxy), and was painted to resemble a semipalmated sandpiper egg (Supplementary 1: Section 3, Picture S2 and S3). This artificial egg was connected to a 12-V 12-Ah battery (Gel Werker) and the RFID. The RFID contained the “heat-SD-card” with a preprogrammed action—to heat or not—for each incubation bout, depending on the identity of the bird on the nest. If the transponder of the focal bird was read and the given incubation bout was set to “heat,” the RFID automatically turned on the artificial egg and the nest was heated (for further details, see Experimental procedure). If the focal bird was absent for more than 10min, the egg automatically turned itself off. The temperature sensor inside the egg was set to 42 °C. Whenever the sensor detected a temperature less than 41.5 °C, the thermistor turned on. Due to thermal inertia of the egg material, the temperature on the surface of the egg (where the bird was touching it) was approximately 40 °C (±1 °C), which is at the higher end of incubation temperatures of semipalmated sandpipers. The actual energy provided by the artificial egg to the nest during incubation was measured by a data logger (SH1, Jan Petrů, http://www.forestspy.com) connected between the 12-V battery and the artificial egg. The logger recorded the energy consumption of the artificial egg every 50th of a second.

#### Experimental procedure

The general aim was to conduct the heating experiment such that it could detect a possible 55-min effect on bout length, that is, the effect reported in Cresswell et al (2003), with sufficient statistical power (0.8; recommended by Cohen 1988).

The exact experimental procedure was based on sets of a priori power analyses (described in Supplementary 1: Section 4, A Priori Power Analyses). Thus, using 50 incubation monitoring systems, the specific aim was to collect incubation data for 4 incubation bouts in each bout category (i.e., before treatment, treated, and after treatment) for both the treated and the untreated parent in at least 22 treated nests (experimentally heated) and 22 control nests (natural nests without any treatment and without artificial egg).

In the field, we assigned the first nest found as treated with the male as the treated parent, the second nest as control with the male as the “treated” parent, the third nest as treated with the female as the treated parent, the fourth nest as a control with the female as “treated,” and so on. If the treated-assigned nest failed before the application of the treatment (e.g., due to predation), we adjusted the assignment of the treatment to the sex in the remaining, not yet treated nests, so that the final sample of female- and male-treated nests remained similar (details in Supplementary 1: Section 5, Sample Sizes). The sex of individuals was known from previous years or estimated from measurements and later confirmed by molecular analyses (Bulla et al. 2014a).

**Figure 1:**
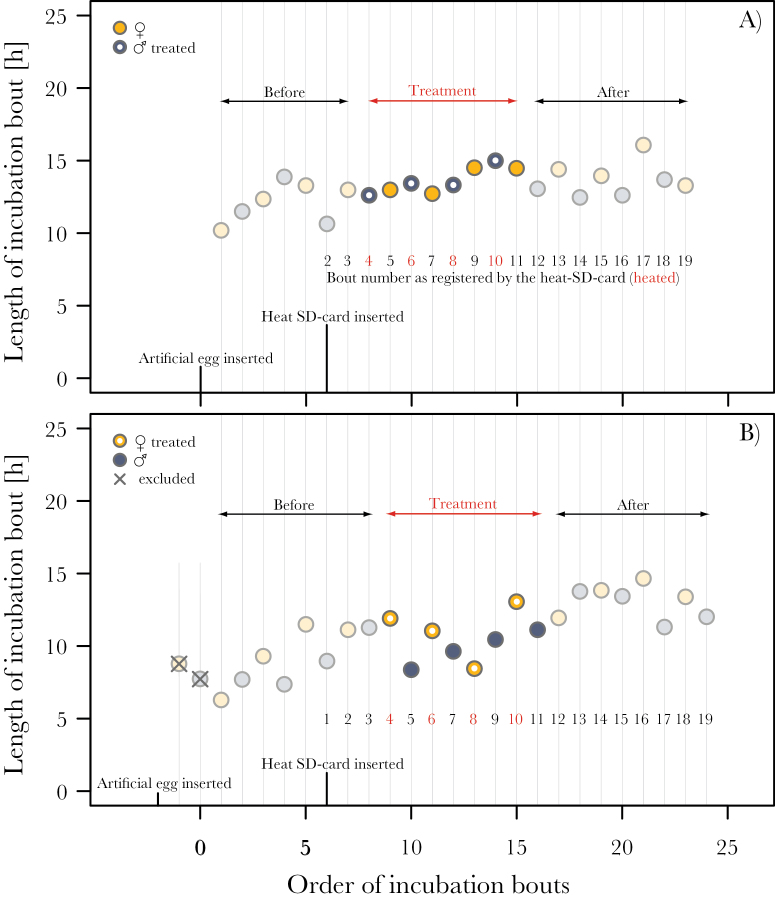
Examples illustrating the experimental procedure. The first bout after introduction of the artificial egg marked the start of the before-treatment period (first circle), which lasted until the first heated bout (first circle emphasized by a white point inside); maximum 4 before-treatment bouts per individual were used in the statistical analyses (i.e., earlier bouts were excluded; indicated by crosses in (B). Once the incubation monitoring system received a “heat-SD-card” (sixth circle in (A) and sixth uncrossed circle in (B), it started to count the incubation bouts (black and red numbers). The egg produced heat during bout 4, 6, 8, and 10 (depicted in red and emphasized by white points inside the circles); these bouts together with the subsequent bouts of the untreated parent (5, 7, 9, and 11) were called treatment bouts. The following bouts (maximum 4 per individual) were defined as after-treatment bouts. If the treated bird incubated when the heat-SD-card was inserted, this bout was recorded as number 2 (A); if the untreated bird incubated, this bout was recorded as number 1 (B). Similar graphs depicting the raw data for all treated and control nests are in Supplementary 2.

In the treated nests, the chronology of the experiment across the season was as follows. We introduced the artificial egg to the treated nests between the 2nd and 11th day of incubation (median = 6th day of incubation; *N* = 21 nests). During the same nest-visit, we exchanged the SD-card of the RFID, which allowed the identification of parents transpondered in the previous year and a check of whether birds transpondered in the current year were successfully detected by the system. Three days later, between the 6th and 14th day of incubation (median = 9th day of incubation; *N* = 21 nests), we connected the artificial egg to the 12-V battery and exchanged the SD-card in the RFID for the “heat-SD-card.” If the treated bird incubated when the heat-SD-card was inserted, the treatment started with the third incubation bout after the insertion (Figure 1A); if the untreated bird incubated, the treatment started with the fourth incubation bout after the insertion (Figure 1B). This led to variable number of before-treatment bouts. The artificial egg was heated on 4 consecutive bouts of the treated bird (Figure 1). Before-treatment bouts were defined from the first bout after the insertion of the artificial egg until the first heated bout (as indicated in Figure 1), but maximum 4 before-treatment bouts per individual were used for statistical analyses (Figure 1B); treated bouts were the heated bouts of the treated parent (4, 6, 8, and 10) and the subsequent bouts of the untreated parent (5, 7, 9, and 11; as indicated in Figure 1); later bouts of both birds were classified as after-treatment bouts (as indicated in Figure 1), and the first 4 after-treatment bouts per individual were used for statistical analyses.

In the control nests, before-treatment bouts of the “treated” bird were assigned from day 4 of incubation until day 7, then 4 “treated” (control) bouts were assigned and the remaining bouts were after-treatment. In 6 nests, the before-treatment bouts were assigned to start before day 4 to be able to include at least 1 after-treatment bout (in 2 nests that failed early), or at least 1 before-treatment bout (in 4 late-found nests). As in the treated nests, the bouts of the “untreated” birds were assigned following the assignment for the “treated” bird.

The final data set consisted of 25 control nests (24 in case of comparison of incubation constancy) and 21 treated nests (the distribution of bouts for all treated and control nests is in Supplementary 1: Section 5, Table S2, and the raw data for each nest in Supplementary 2).

### Background of the insulation experiment

The insulation experiment was conducted between 10 June and 4 July 2000 in the same study area, on the same species and using a similar, RFID-based, incubation monitoring system as in the heating experiment; details are in Cresswell et al. (2003). Briefly, the insulation quality of nests was improved by a polystyrene drinking cup (cut down to 5cm and painted dull brown) inserted under the lining of the nest. The experiment used a matched-pairs cross-over design, that is, one nest of a pair was insulated for 48h, while its paired nest acted as a control (first period of the experiment), then the insulation was swapped within the pair and the previously insulated nest served as a control for 48h, while its paired nest was insulated (second period of the experiment). The statistical analyses were conducted on the mean bout length per nest and treatment (control or insulated). Nests, not individual birds, were the units of analyses. The reported 55-min effect (95% CI: 11–99min) is based on the within-nest comparison (paired *t-*test) of mean length of untreated incubation bouts and mean length of insulated incubation bouts (which crucially included some control bouts outside the two 48-h periods to increase the sample size incubation bouts). The analysis did not control statistically for a period effect (i.e., whether control or insulation occurred within the first or second 48-h experimental period). Also, contrary to the requirements of the experimental design, the data set was unbalanced. Hence, we reanalyzed the data, following the procedures outlined in Supplementary 1: Section 1, which also includes full details on the sources of bias and error in the original analysis.

### Artificial experiment

We tested the difference in the strength of the treatment between the heating and insulation experiment with an artificial experiment. On 3 June 2013, we made 3 artificial nest scrapes and equipped each with an artificial egg (heat-producing egg described in Heating treatment). The experiment consisted of 3 treatments: artificial egg turned on (as in the heating experiment), artificial egg turned off (control), and artificial egg turned off but nest insulated (as in the insulation experiment; see Supplementary 1: Section 3, Picture S2). We made an artificial brood-patch by embedding a heat-producing egg in polystyrene (Supplementary 1: Section 3, Picture S3) and placed it over each nest scrape in contact with the artificial egg (Supplementary 1: Section 3, Picture S4). The heat-producing egg inside the “brood-patch” was connected to a data logger (described in Heating treatment) that registered its energy consumption for 50min. Hence, this setup allowed measuring how much energy the brood-patch needed to keep the artificial egg warm in each of the 3 treatments. We repeated the experiment on the same day, such that each nest scrape consecutively received each of the 3 treatments. The whole procedure was repeated 10 days later.

### Statistical analyses

R version 3.0.3 (R Core Team 2014) was used for all statistical analyses, the “lme4” package (Bates and Maechler 2010) for mixed-effects modeling and the “rmeta” package (Lumley 2012) for the meta-analysis. The results of the linear and mixed models include adjusted approximations of confidence intervals (CIs) and *P* values based on multiple comparisons (simultaneous inference) of predictors using the “glht” function from the “multcomp” package (Hothorn et al. 2008). This function allows immediate test of only specifically defined hypotheses (reported in the Results; the full statistical summaries are reported in the Supplementary 1: Section 6, Models). Uncertainties are reported as 95% CIs.

### Data and R-scripts

Data are available from figshare.com digital repository at http:// figshare.com/articles/Data_from_Biparental_incubation_ scheduling_no_experimental_evidence_for_major_energetic_constraints_/1035052 (Bulla et al. 2014b). 

R-script (of the statistical analyses, figures, power analyses, etc.) is available from figshare.com digital repository at http://figshare.com/articles/R_script_from_Biparental_incubation_scheduling_no_experimental_evidence_for_major_energetic_constraints/1035048 (Bulla et al. 2014c).

## RESULTS

### Energy provided by the heating

The actual energy provided by the heated artificial egg to the nest was measured at 3 nests (during 5 female and 7 male incubation bouts). The artificial egg provided 537 mW (95% CI: 476–597 mW; *N =* 12 incubation bouts; variance of random intercept [nest] = 175, residual variance = 10750). Assuming that a 27-g semipalmated sandpiper invests between 68 and 284 mW to keep 4 eggs at incubation temperature (based on Norton 1973; Biebach 1979; Vleck 1981; described in Supplementary 1: Section 7, Estimating Energetic Demands of Incubation), the artificial egg provided approximately 2–8 times more energy than required to incubate the clutch and provided energy equivalent to approximately 40% of the bird’s resting metabolic rate (Norton 1973). Thus, the heating treatment should have led to a substantial reduction in the overall energetic demands of incubation for the treated parent (including a reduction in the costs of thermoregulation).

### Heating experiment

**Figure 2:**
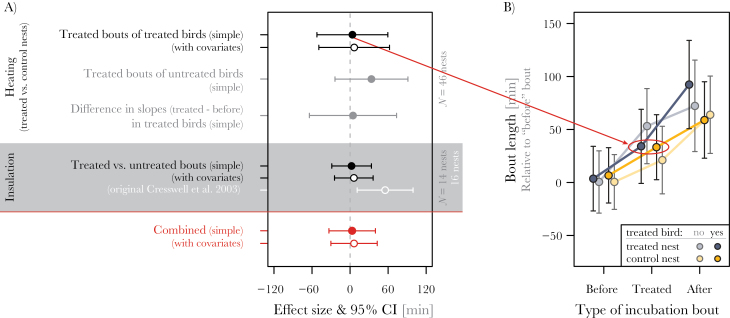
No major effects of heating (A and B) or nest insulation (A, gray area) on the length of incubation bouts. In (A), the red dots show the combined effect size for the heating and insulation experiment; filled circles: simple model estimates (statistical details are in Supplementary 1: Section 6, Tables S3 and S5); open circles: estimates from complex models with covariates (statistical details are in Supplementary 1: Section 6, Tables S4 and S6); in white: the estimate from the original insulation study (Cresswell et al. 2003). In (B), the red ellipse highlights the main comparison of interest, namely the difference between treated bouts in treated nests (heated bouts) and control nests (nonheated bouts). The values depicted in (B) are derived from the simple model using the “allEffects” function of the R package “effects” (Fox 2003); shown are bout lengths relative to the before-treatment bout length (i.e., for each individual, the mean before-bout length was calculated and subtracted from all incubation bouts). This allowed direct comparison of the treatment effects among nests, and controlled for changes in bout length with date and with day of incubation.

To investigate whether the heating treatment changed the length or constancy of incubation, we specified the 3-way interaction of interest, namely whether the nest, parent, and incubation bout were treated (Supplementary 1: Section 6, Tables S3–S6). We used both simple models without covariates and complex models including covariates known to explain variation in the dependent variable (Bulla et al. 2014a). In each model, we controlled for pseudoreplicaton by adding nest as a random intercept and day of incubation (quadratic) as a random slope. Because the simple and complex models gave qualitatively similar results (Supplementary 1: Section 6, Tables S3–S6), here we only describe in detail the outcome of the simple models and for the complex model present the estimates of interest in Figure 2A.

There was no major effect of the heating on the length of incubation bouts (Figure 2). During heating, the treated birds in treated nests had 3.9min (95% CI: −52 to 59.8min) longer incubation bouts than “treated” birds in control nests (*P* = 1, *N*_total_ = 976 incubation bouts from 46 nests; see Figure 2A); the statistical power to detect an effect of 55min was >0.8 (detailed in Supplementary 1: Section 4). In comparison, during the treatment period, the untreated birds in treated nests had 34min (−24 to 92min) longer incubation bouts than untreated birds in control nests—an effect in the opposite direction to that expected for the untreated partner (*P* = 0.4; *N*_total_ = 976 incubation bouts from 46 nests; Figure 2A). The change in the length of incubation bouts from before-treatment to treatment in the treated birds in treated nests was 5min (−64 to 74min) larger than in “treated” birds in control nests (*P* = 1; Figure 2A, difference in slopes); here, the statistical power to detect an effect of 55min was <0.6 (Supplementary 1: Section 4, Figure S2). We did not test for a sex-specific effect of the heating treatment because of low statistical power (<0.2; Supplementary 1: Section 4, Figure S2).

The lack of response to the heating in terms of incubation bout length was not compensated by major changes in the constancy of incubation, that is, in the proportion of actual incubation within bouts. During heating, the bouts of treated birds in treated nests had 0.5% (95% CI: −2.0 to 2.9%) larger incubation constancy than “treated” bouts in control nests (*P* = 0.95, *N*_total_ = 952 incubation bouts from 45 nests). During the treatment period, the bouts of untreated birds in treated nests had 0.3% (−2.3 to 2.9 %) lower incubation constancy than the bouts of “untreated” birds in control nests (*P* = 0.98). The change in the incubation constancy from before-treatment to treatment in the treated birds in treated nests was 0.4% (−2.8 to 3.7%) larger than in “treated” birds in control nests (*P* = 0.98).

Moreover, the presence and duration of exchange gaps was unaffected by the heating (Supplementary 1: Section 6: Tables S7 and S8).

In sum, we found no evidence that reduced energetic demands of incubation (through egg heating) changed the length or constancy of incubation bouts in biparentally incubating semipalmated sandpipers.

### Insulation experiment: reanalysis of Cresswell et al. (2003)

To verify whether the different outcomes of the heating experiment and the insulation experiment (as originally reported) hold, after taking into account the matched-pair cross-over experimental design (Hills and Armitage 1979; Jones and Kenward 1989; Díaz-Uriarte 2002), we reanalyzed the data from the insulation experiment. A detailed description of the original analysis and the reanalysis is in Supplementary 1 (Section 1, Reanalysis of the Insulation Experiment).

Once the analysis controlled for the period within which the control or treatment was applied (first or second 48h), in all models (3 using nest-mean bout length, 2 mixed models on individual bout lengths) the originally reported effect (an increase in bout length of 55min after insulation) disappeared (Figure 2A; Supplementary 1: Section 1, Table S1). Here, we only describe in detail the outcome of the 2 mixed models.

The treated bouts were 2.7min (95% CI: −28.6 to 33.9min) longer than untreated bouts (*P* = 0.87, *N* = 45 treated and 42 untreated bouts from 14 nests and 7 pairs; Figure 2A; statistical details are in Supplementary 1: Section 6, Table S9, the raw data for each nest are in Supplementary 3). Even after controlling the model for the confounding variables (sex and day of incubation; Bulla et al. 2014a), the estimated difference remained small; treated bouts were 6.2min (95% CI: −24.3 to 36.6min) longer than untreated bouts (*P* = 0.69; Figure 2A; statistical details are in Supplementary 1: Section 6, Table S10).

In sum, contrary to the original finding (Figure 2A), we found no evidence that reduced energetic demands of incubation by nest insulation increased the length of incubation bouts.

### Artificial experiment: comparing the treatments

To find out in how far the results of the 2 experiments are comparable, we investigated whether the heating and insulation experiment differed in the strength of their treatment. The approximate amount of energy needed to “incubate” nests was measured with 3 artificial nest scrapes (heated, control, and insulated).

The artificial brood-patch consumed the least energy when the nest was heated: on average, 233 mW (95% CI: 111–354, *P* < 0.0001) less than in control nests and 158 mW (95% CI: 36–279, *P* = 0.0079) less than in insulated nests (mixed model with “nest” and “day of the experiment” as random intercepts; *N* = 15, 50-min measurements acquired during 2 days with 3 repeats per day from each of 3 nest scrapes; 3 missing values are due to system failure at one nest scrape during day 1; statistical details are in Supplementary 1: Section 6, Table S11).

These results indicate that—at least under the described experimental conditions—the heating treatment was stronger than the insulation treatment, and that both potentially saved energy for the incubating birds.

### Combined effect of the 2 experiments

To estimate the overall effect of reduced energetic demands of incubation on the length of incubation bouts, we performed a meta-analysis that combined the effects from the heating and insulation experiment and weighted them by the number of nests. The combined effect of heating and insulation on the length of incubation bouts was a prolongation of 3.6min (95% CI: −33 to 40; red dot in Figure 2A) for the estimates from the simple models and 6.5min (95% CI: −30 to 43; red circle in Figure 2A) for the estimates from models that controlled for the confounding variables. Both combined effects demonstrate no major energetic constraint on the length of incubation bouts.

## DISCUSSION

We found no evidence for major energetic constraints on biparental incubation-scheduling in this system. Both the new experimental heating and the previous experimental insulation (here reanalyzed) had little influence on the length of incubation bouts (Figure 2). The experimental heating also left the incubation constancy unaffected (Supplementary 1: Section 6, Tables S5 and S6) and did not influence the presence or duration of exchange gaps (Supplementary 1: Section 6, Tables S7 and S8). Further, neither the parent that received heat nor the untreated parent changed their incubation bout length (Figure 2) or constancy (Supplementary 1: Section 6, Tables S5 and S6). These results indicate that it is unlikely that the energetic reserves either of the incubating parent or of the off-nest parent determine incubation-scheduling in semipalmated sandpipers. Below, we discuss potential limitations of our approach, the benefit of replicating a previously published study, and the main biological implications of our results.

### Limitations

The absence of major energetic constraints on the length and constancy of incubation bouts under the current experimental setup does not exclude the possibility of minor energetic constraints on incubation-scheduling or the possibility that such constraints become relevant when the demands of incubation are more extreme.

First, although the heating treatment was severe (providing energy equivalent of up to 40% of the parent’s resting metabolic rate), the setup had sufficient statistical power only to detect a change in the length of treated incubation bouts higher than 52min (Supplementary 1: Section 4, Figures S1–S4). Thus, smaller changes in bout length were likely to be undetected. However, the effect of heating was only in the order of a few minutes (Figure 2A), equivalent to a 0.2% change in the average incubation bout of semipalmated sandpipers (Bulla et al. 2014a).

Second, regardless of the strength of the treatment, we only manipulated the birds in one direction (decreasing energetic demands) and we did not measure the energy expenditure or weight loss of the incubating birds. Thus, we do not know how the treatments influenced the birds’ condition. We cannot exclude the possibility that an increase in the energetic demands of incubation (e.g., due to severe weather or through experimental cooling of the nest) would influence the length of incubation bouts. Contrary to this prediction and in line with our findings, experimental cooling of Kentish plovers’ nests increased (not decreased) incubation constancy (Kosztolanyi et al. 2009). However, this experiment was conducted under conditions that were less stressful to the birds (temperatures within their thermo-neutral zone) and effects on incubation bout length were not measured.

In sum, our results cannot fully exclude the existence of some energetic constraints on incubation-scheduling, but suggest that such constraints—if they exist—will only appear under severe conditions. Future studies would benefit from measuring changes in the condition of manipulated birds.

### Replication

Our finding revises previously published work (Cresswell et al. 2003), which suggested (using experimental nest insulation) that incubation-scheduling in semipalmated sandpipers was energetically constrained and driven by the energy reserves of the incubating parent. We failed to replicate this finding using a nest-heating experiment that targeted a single parent and was carried out in the same study area, on the same species and using a similar incubation monitoring system. This then prompted a reanalysis of the data from the earlier insulation experiment that targeted both parents. Using a rigorous statistical control of the matched-pair cross-over experimental design, the originally reported 55-min effect disappeared. Our results further demonstrate that both experiments should have had large effects on the energetic demands of incubation, whereby the heating procedure saved more energy than the insulation procedure. Furthermore, both experiments were carried out in relatively similar environmental conditions (apart from rain; Supplementary 1: Section 2, Figure S1). In sum, our findings amend the previously published results and interpretations, and demonstrate the benefit of replicating published experiments and the advantage of making data freely available for reanalysis.

### Biological implications

The lack of major energetic constraints on biparental incubation-scheduling has 3 biological implications. First, the absence of such constraints in relatively severe high Arctic conditions is perhaps surprising, but suggests either that food is abundant, and hence parents less constrained by foraging time, or that the incubating parents of Arctic breeding species are adapted to buffer fluctuations in energetic demands of incubation (e.g., due to a spell of colder weather), just as their eggs (developing embryos) are adapted to survive prolonged conditions below the optimal for embryonic development (reviewed by Hicklin and Gratto-Trevor 2010).

Second, our findings suggest that biparental incubation-scheduling is less energetically constrained than uniparental incubation (e.g., Aldrich and Raveling 1983; Bryan and Bryant 1999; Reid et al. 1999; Cresswell et al. 2004; Ardia et al. 2009). This implies that individual semipalmated sandpipers—and perhaps most biparentally incubating species—might be able to incubate continuously for much longer than they actually do (see also Kosztolanyi et al. 2009). Therefore, other factors, such as predation risk, circadian fluctuations in prey availability, or synchronization of the daily rhythms of the 2 parents (discussed in Bulla et al. 2014a), may play a more important role in determining the length of incubation bouts.

Third, our findings also revise Cresswell et al.’s (2003) conclusion that the incubating parent may play an important role in driving incubation-scheduling. The fact that our heating experiment, which manipulated energy demands of only one of the 2 pair members, did not cause a change in bout length in either the treated or the untreated parent implies that we still do not understand which parent drives the length of incubation bouts. Knowledge about how parents behave while off-nest or near the nest during the exchange (e.g., whether the returning bird waits for a signal from its incubating partner, or whether the incubating parent waits for its off-nest partner to return) may help understand the factors determining biparental incubation-scheduling.

## CONCLUSIONS

Our study illustrates the merit of replicating previously published experiments, as well as the usefulness of making data of published work freely available. Most importantly, our results reveal that it is unlikely that biparental incubation-scheduling in the semipalmated sandpiper is driven by major energetic constraints and that we still do not understand what drives variation in biparental incubation patterns, both in this and in other species.

## SUPPLEMENTARY MATERIAL

Supplementary material can be found at http://www.beheco.oxfordjournals.org/

## FUNDING

This work was funded by the Max Planck Society. 

## Supplementary Material

Supplementary Data
