# Supplementary material for: Biparental incubation-scheduling: no experimental evidence for major energetic constraints
Source: Behav Ecol. 2014 Sep 3;26(1):30–7. doi: 10.1093/beheco/aru156 (PMC4309980; doi:10.1093/beheco/aru156)
Supplement: Supplementary Data [file supp_26_1_30__index.html]

Biparental incubation-scheduling: no experimental evidence for major energetic constraints — Supplementary Data 

# Biparental incubation-scheduling: no experimental evidence for major energetic constraints

## Supplementary Data

Data files

**Files in this Data Supplement:**

- Supplementary Data - Supplementary Data
- Supplementary Data - Supplementary Data
- Supplementary Data - Supplementary Data
- Supplementary Data - Supplementary Data
