# Supplementary material for: Biparental incubation-scheduling: no experimental evidence for major energetic constraints
Source: Behav Ecol. 2014 Sep 3;26(1):30–7. doi: 10.1093/beheco/aru156 (PMC4309980; doi:10.1093/beheco/aru156)
Supplement: Supplementary Data [file supp_aru156_Supplementary_3_Insulation_experiment.pdf]

INSULATION EXPERIMENT - DATA

Each figure depicts all data for a given nest. Red circles mark incubation bouts excluded by Cresswell et al. (2003) because the incubating bird was disturbed or because the polystyrene-insulation was inserted or removed from the nest. The insertion and removal of insulation is indicated by red point. Incubation bouts indicated by red cross were outside of the 48 h experimental period; these bouts were included by Cresswell et al. as control bouts, but (due to the cross-over experimental design) excluded in the current analyses. Order indicates whether the nest served first as a control and then was treated (CT) or whether it was first treated and then served as a control (TC).

pair 1, nest 6, order = CT

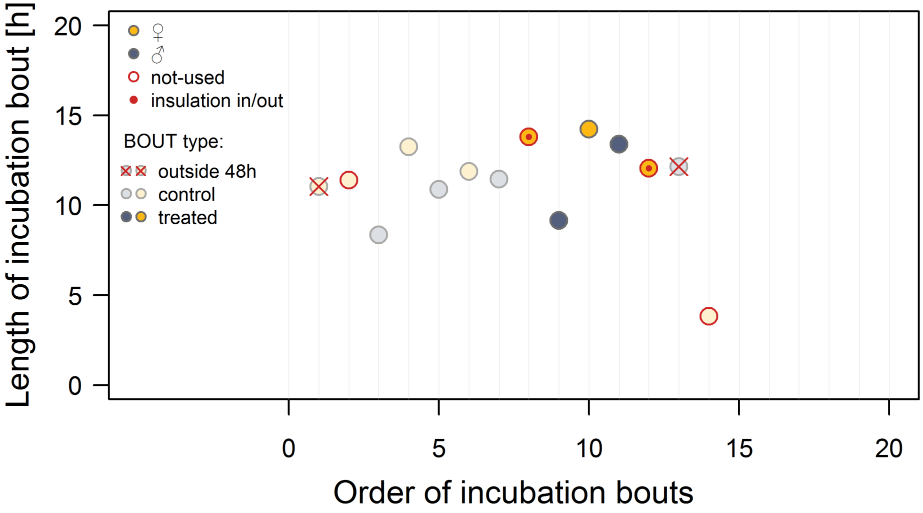

pair 1, nest 2, order = TC

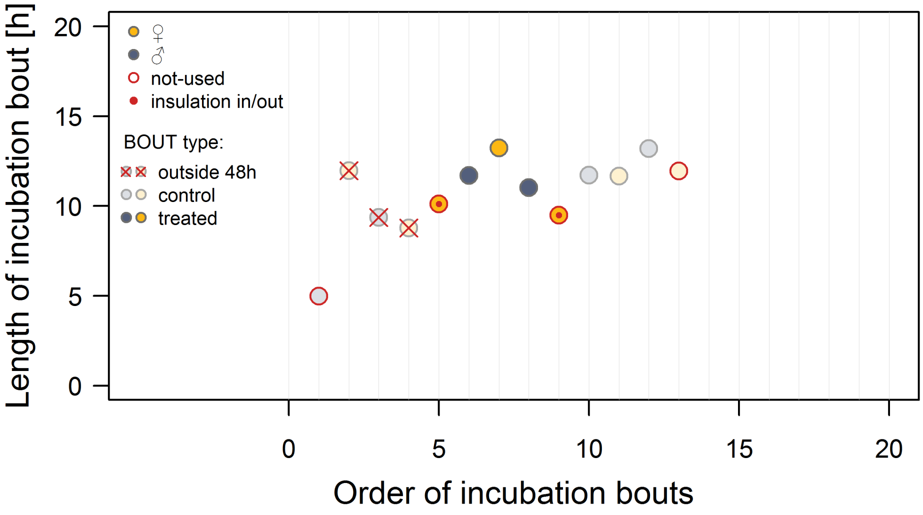

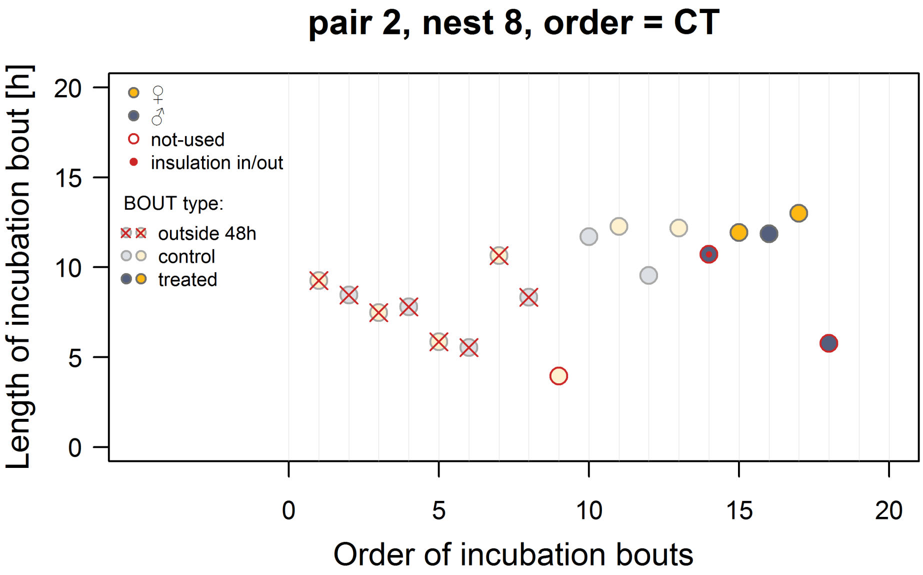
pair 2, nest 8, order = CT
Length of incubation bout [h]
Order of incubation bouts
♀
♂
not-used
insulation in/out
BOUT type:
outside 48h
control
treated
0
5
10
15
20

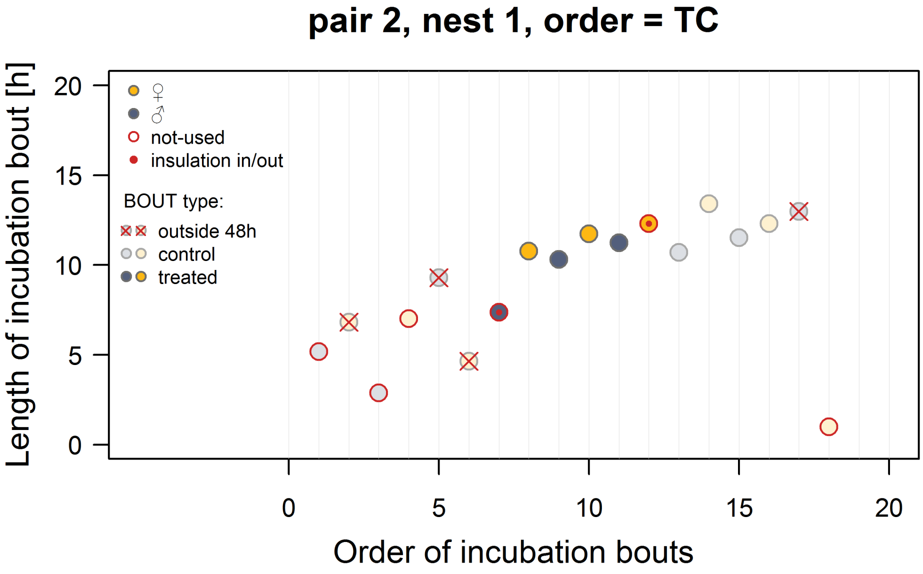
pair 2, nest 1, order = TC
Length of incubation bout [h]
Order of incubation bouts
♀
♂
not-used
insulation in/out
BOUT type:
outside 48h
control
treated
0
5
10
15
20

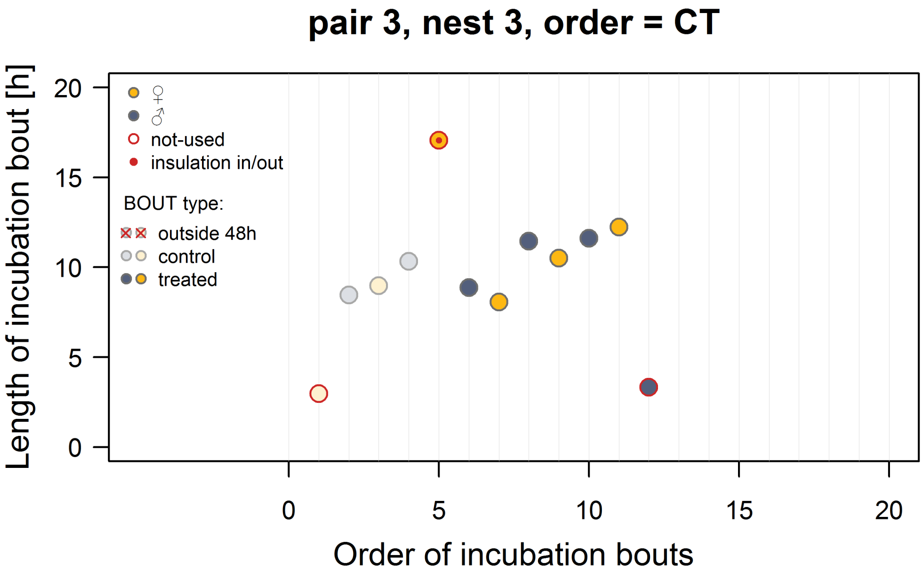
pair 3, nest 3, order = CT
Length of incubation bout [h]
Order of incubation bouts
♀
♂
not-used
insulation in/out
BOUT type:
outside 48h
control
treated
0
5
10
15
20
0
5
10
15
20

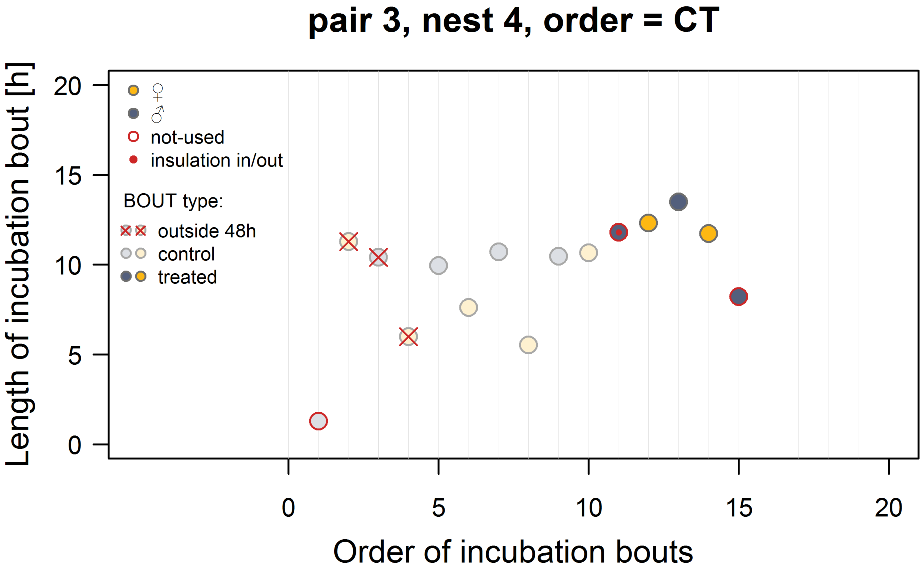
pair 3, nest 4, order = CT
Length of incubation bout [h]
Order of incubation bouts
♀
♂
not-used
insulation in/out
BOUT type:
outside 48h
control
treated
0
5
10
15
20
0
5
10
15
20

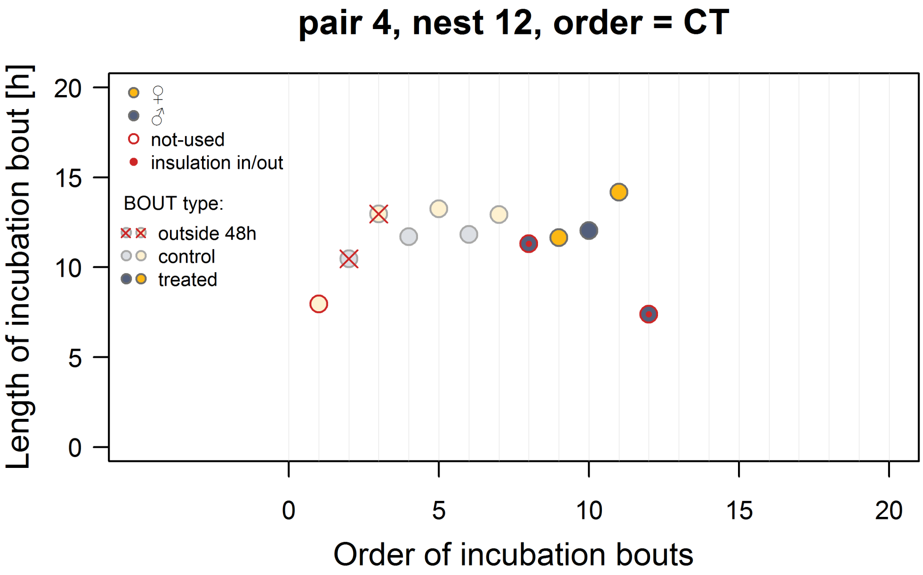
pair 4, nest 12, order = CT
Length of incubation bout [h]
Order of incubation bouts
0
5
10
15
20
♀
♂
not-used
insulation in/out
BOUT type:
outside 48h
control
treated

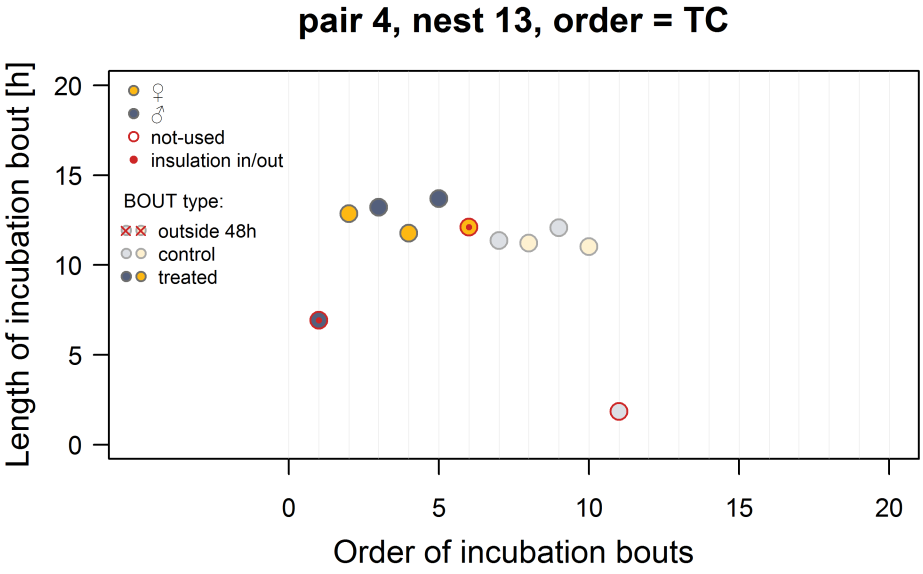
pair 4, nest 13, order = TC
Length of incubation bout [h]
Order of incubation bouts
0
5
10
15
20
♀
♂
not-used
insulation in/out
BOUT type:
outside 48h
control
treated

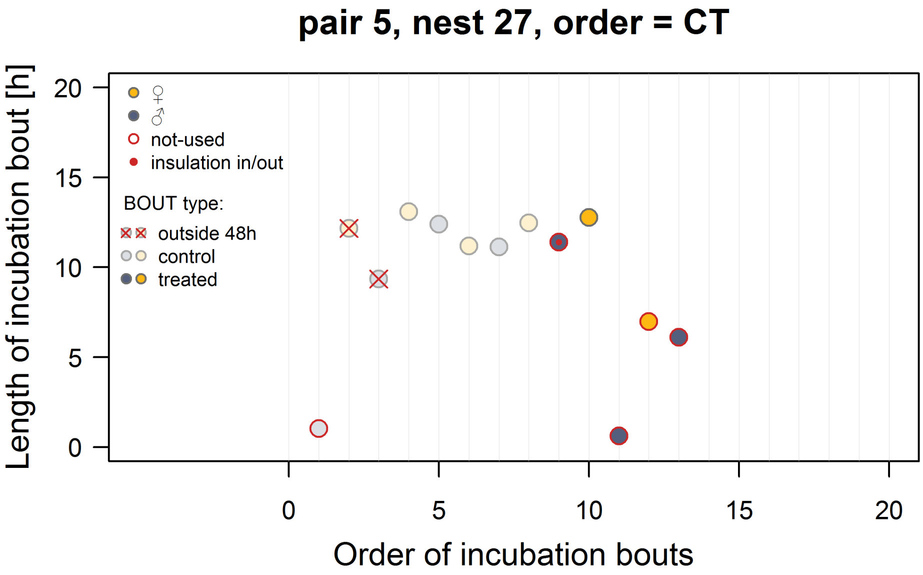
pair 5, nest 27, order = CT
Length of incubation bout [h]
Order of incubation bouts
♀
♂
not-used
insulation in/out
BOUT type:
outside 48h
control
treated
0
5
10
15
20

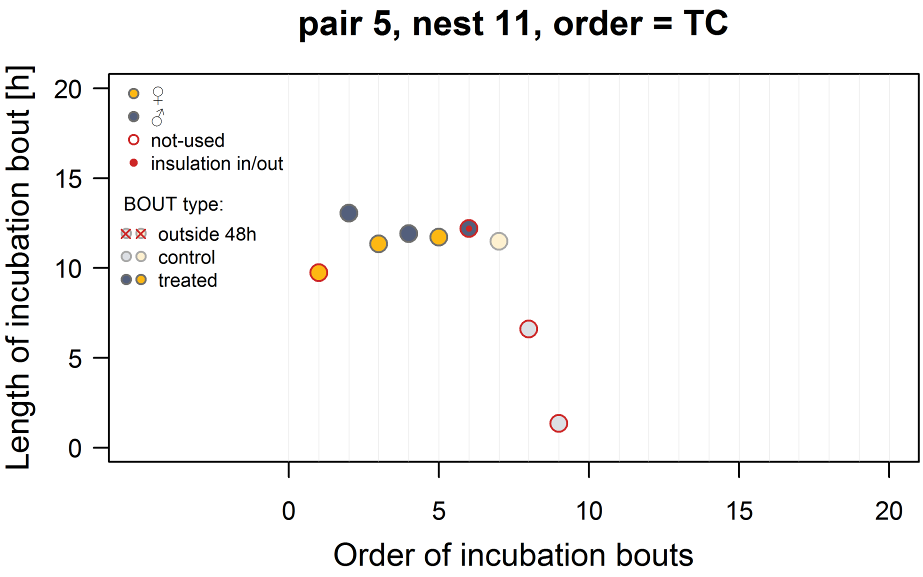
pair 5, nest 11, order = TC
Length of incubation bout [h]
Order of incubation bouts
♀
♂
not-used
insulation in/out
BOUT type:
outside 48h
control
treated
0
5
10
15
20

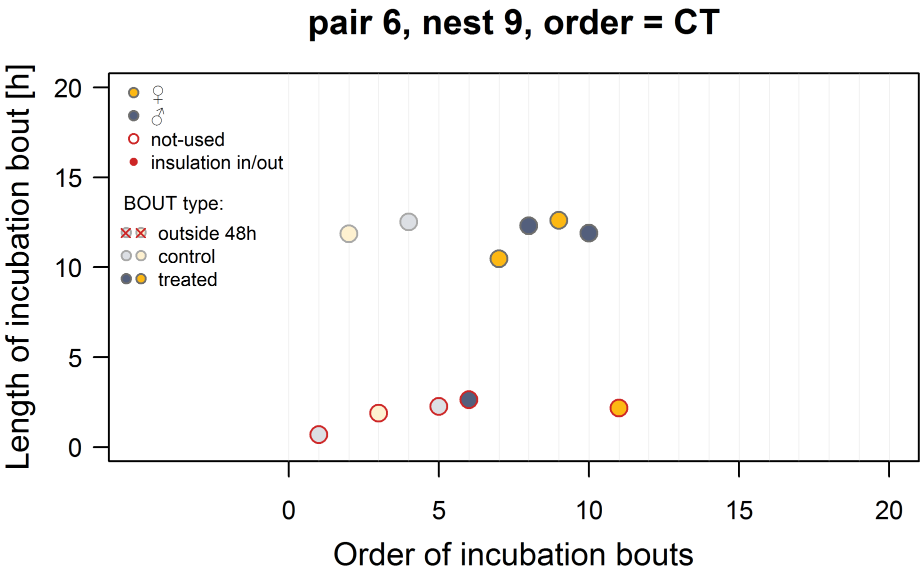
pair 6, nest 9, order = CT
Length of incubation bout [h]
Order of incubation bouts
♀
♂
not-used
insulation in/out
BOUT type:
outside 48h
control
treated

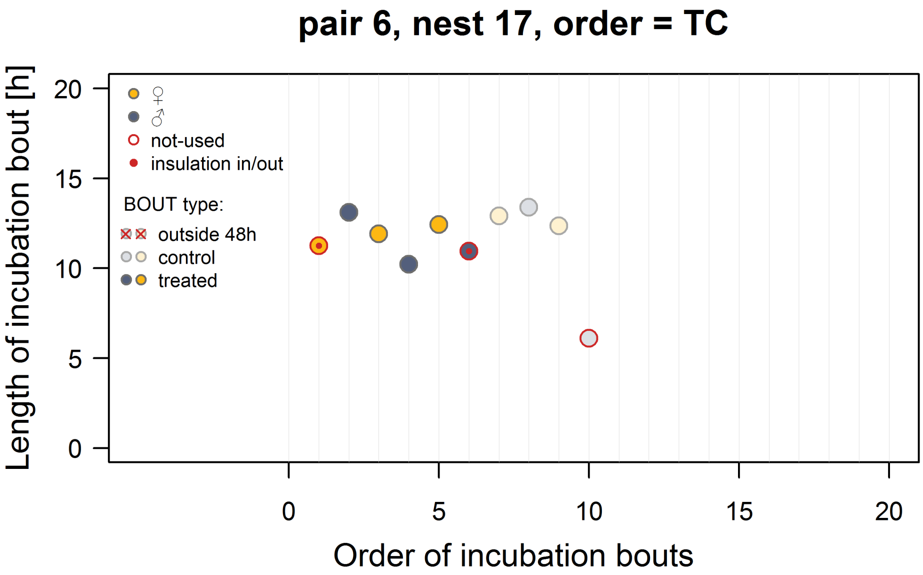
pair 6, nest 17, order = TC
Length of incubation bout [h]
Order of incubation bouts
♀
♂
not-used
insulation in/out
BOUT type:
outside 48h
control
treated

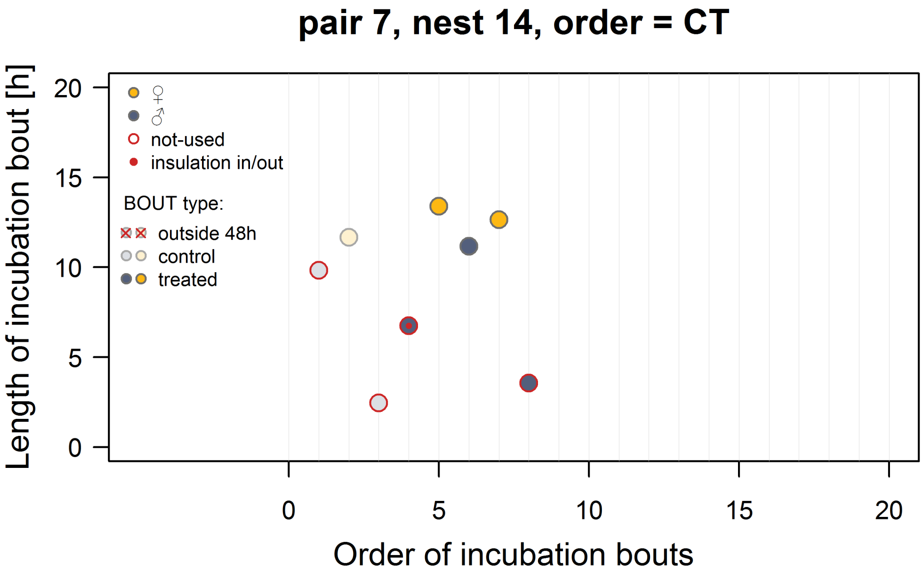
pair 7, nest 14, order = CT
Length of incubation bout [h]
Order of incubation bouts
♀
♂
not-used
insulation in/out
BOUT type:
outside 48h
control
treated
0
5
10
15
20

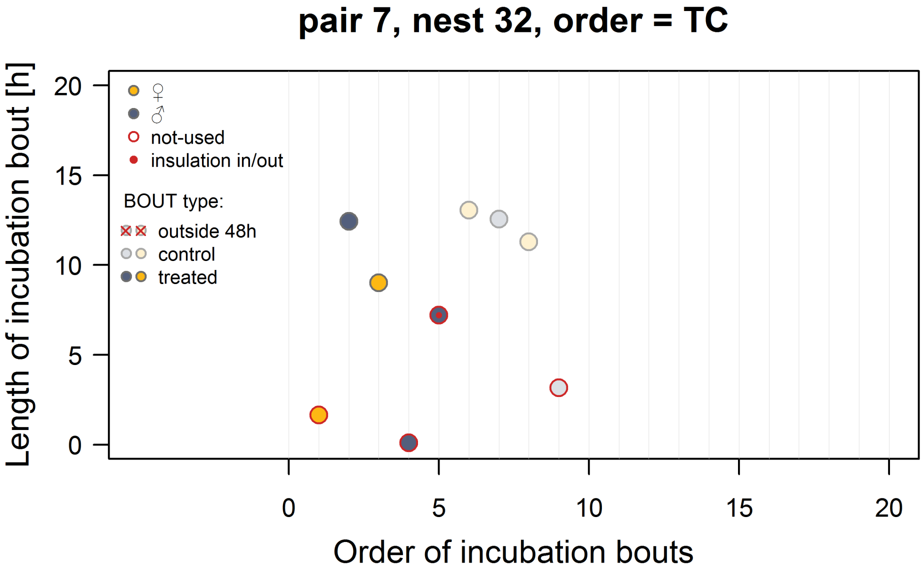
pair 7, nest 32, order = TC
Length of incubation bout [h]
Order of incubation bouts
♀
♂
not-used
insulation in/out
BOUT type:
outside 48h
control
treated
0
5
10
15
20

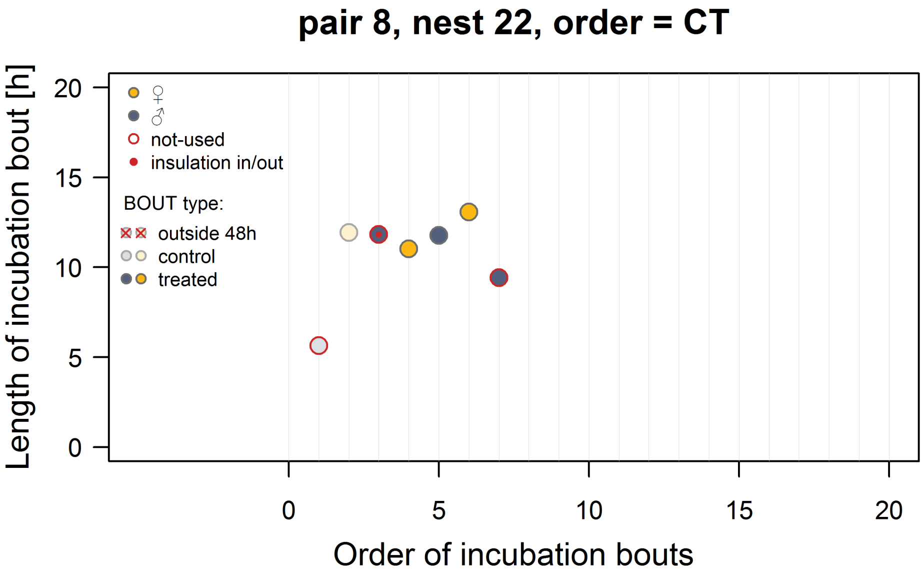
pair 8, nest 22, order = CT
♀
♂
not-used
insulation in/out
BOUT type:
outside 48h
control
treated
Length of incubation bout [h]
Order of incubation bouts
0
5
10
15
20
0
5
10
15
20

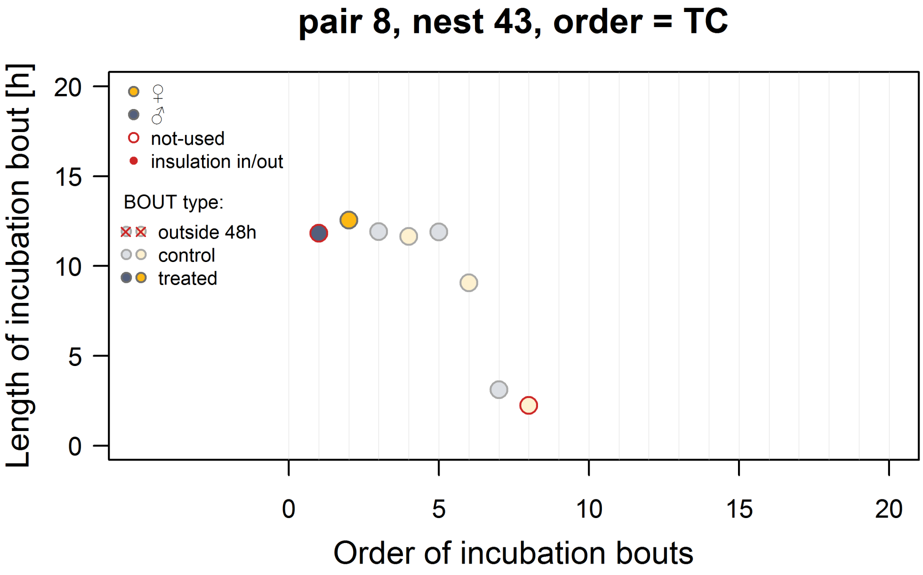
pair 8, nest 43, order = TC
♀
♂
not-used
insulation in/out
BOUT type:
outside 48h
control
treated
Length of incubation bout [h]
Order of incubation bouts
0
5
10
15
20
0
5
10
15
20
