# Supplementary material for: Biparental incubation-scheduling: no experimental evidence for major energetic constraints
Source: Behav Ecol. 2014 Sep 3;26(1):30–7. doi: 10.1093/beheco/aru156 (PMC4309980; doi:10.1093/beheco/aru156)
Supplement: Supplementary Data [file supp_aru156_Supplementary_2_Heating_experiment.pdf]

HEATED-EGG EXPERIMENT - DATA

Each figure depicts all data for a given nest that were included in the statistical models. Light dots represent before treatment or after treatment bouts, dark dots indicate treatment bouts, white point indicates bouts of the treated (heated) bird.

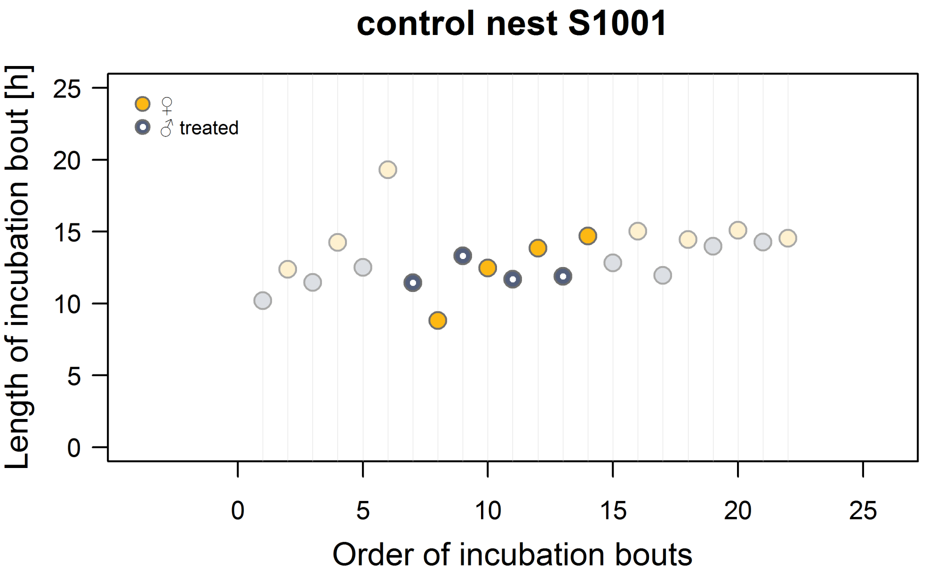
control nest S1001
♀
♂ treated
Length of incubation bout [h]
Order of incubation bouts
0
5
10
15
20
25
0
5
10
15
20
25

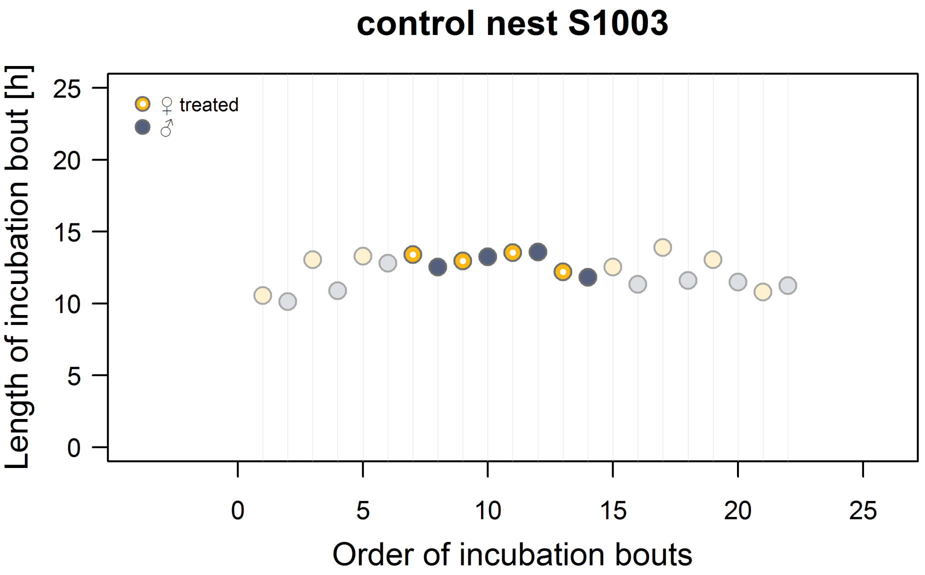
control nest S1003
♀ treated
♂
Length of incubation bout [h]
Order of incubation bouts
0
5
10
15
20
25

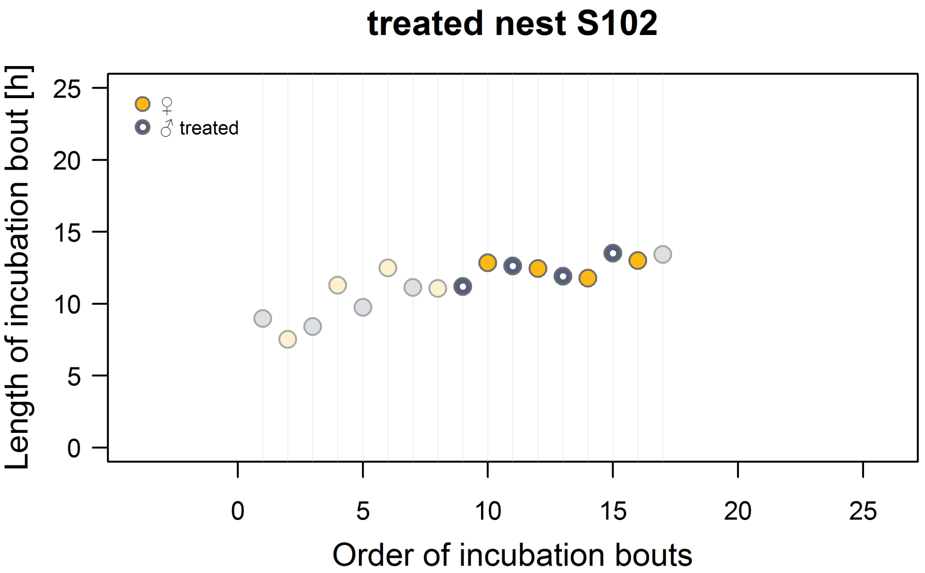
treated nest S102
♀
♂ treated
Length of incubation bout [h]
Order of incubation bouts
0
5
10
15
20
25

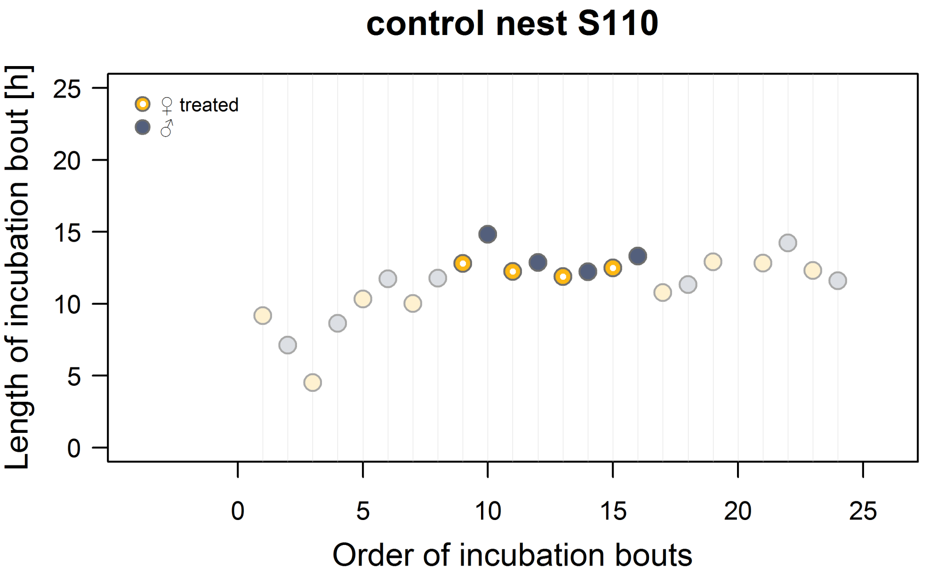

control nest S110
♀ treated
♂
Length of incubation bout [h]
Order of incubation bouts
0
5
10
15
20
25

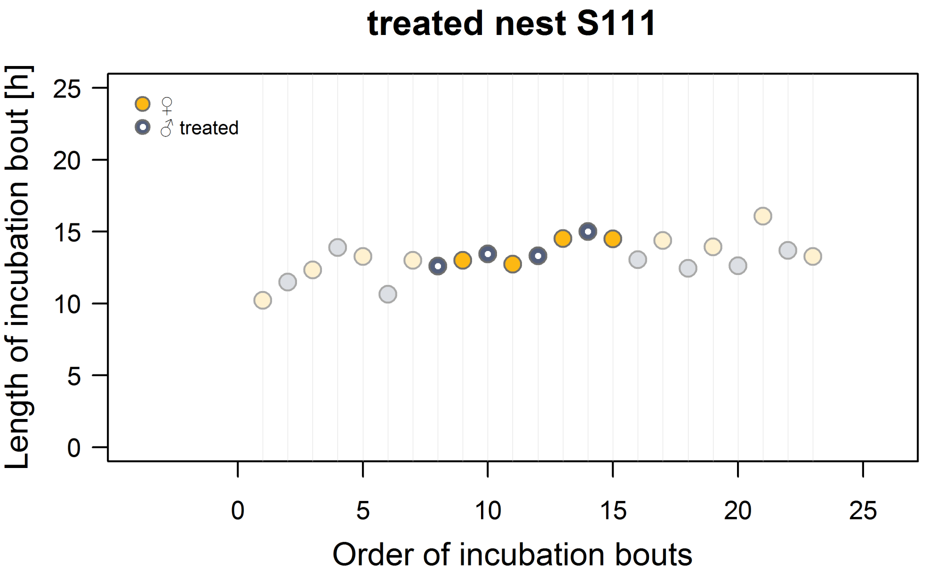

treated nest S111
♀
♂ treated
Length of incubation bout [h]
Order of incubation bouts
0
5
10
15
20
25

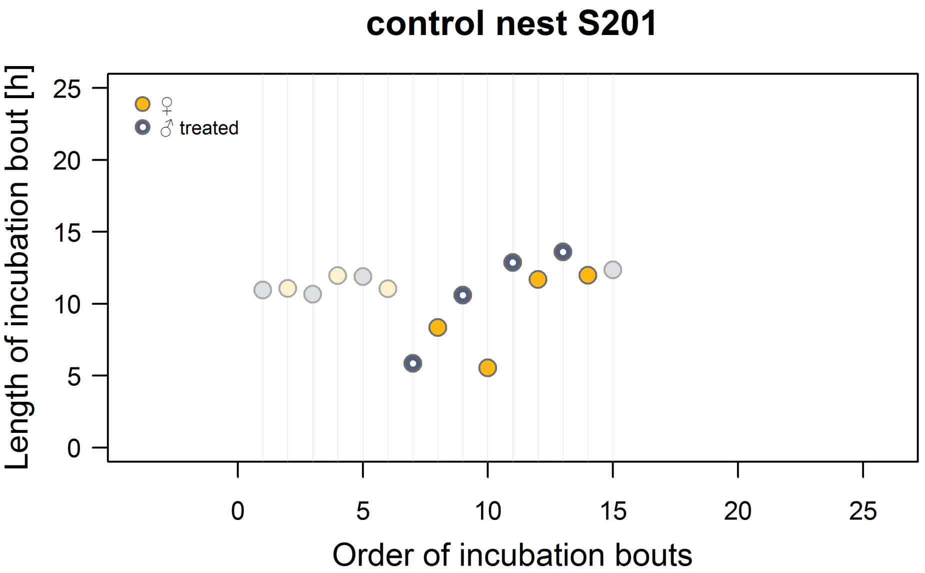
control nest S201
♀
♂ treated
Length of incubation bout [h]
Order of incubation bouts
0
5
10
15
20
25

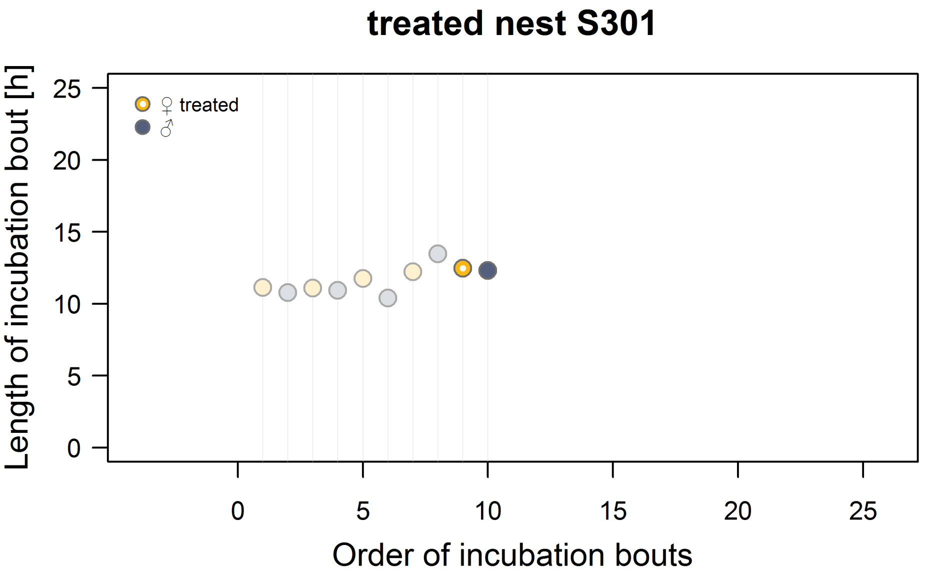
treated nest S301
♀ treated
♂
Length of incubation bout [h]
Order of incubation bouts
0
5
10
15
20
25

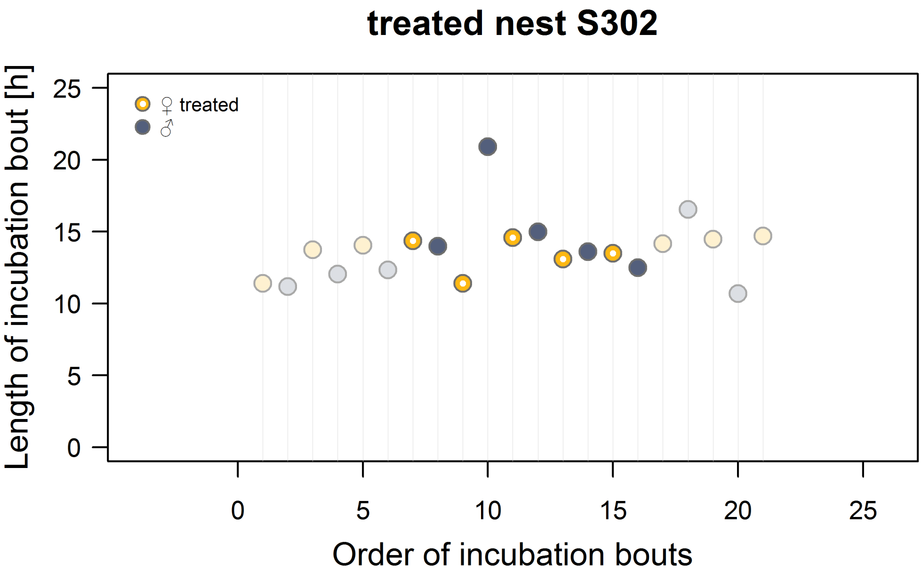

treated nest S302
♀ treated
♂
Length of incubation bout [h]
Order of incubation bouts
0
5
10
15
20
25

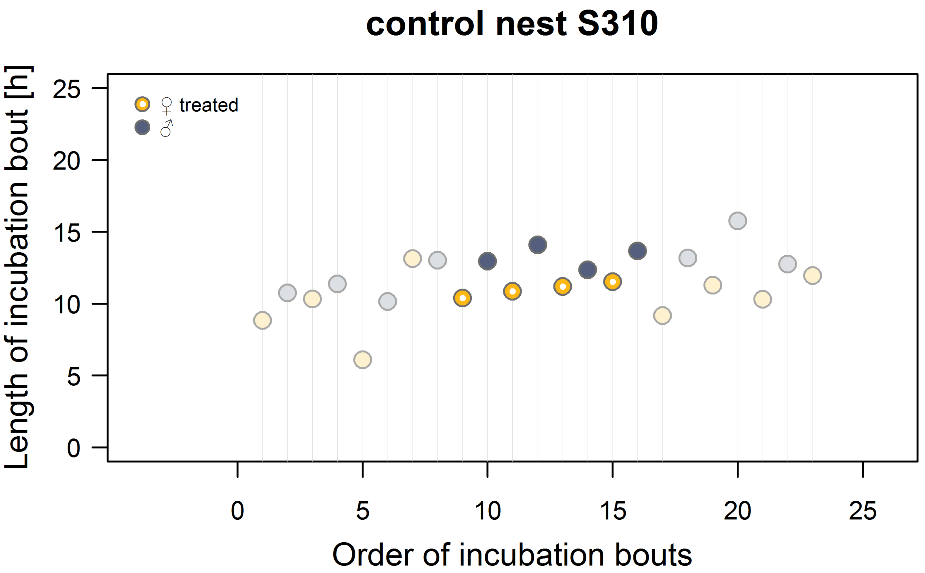
control nest S310
♀ treated
♂
Length of incubation bout [h]
Order of incubation bouts
0
5
10
15
20
25

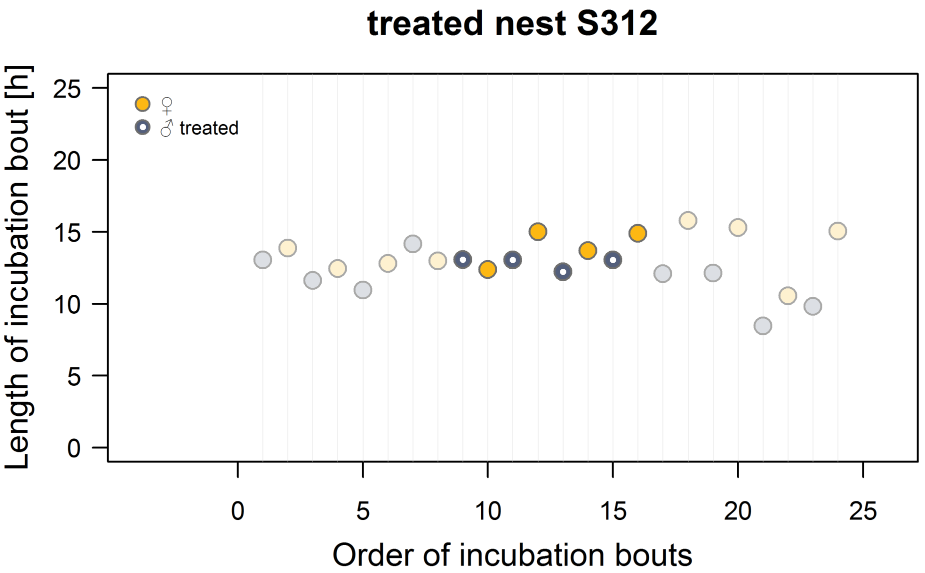
treated nest S312
♀
♂ treated
Length of incubation bout [h]
25
20
15
10
5
0
0
5
10
15
20
25
Order of incubation bouts

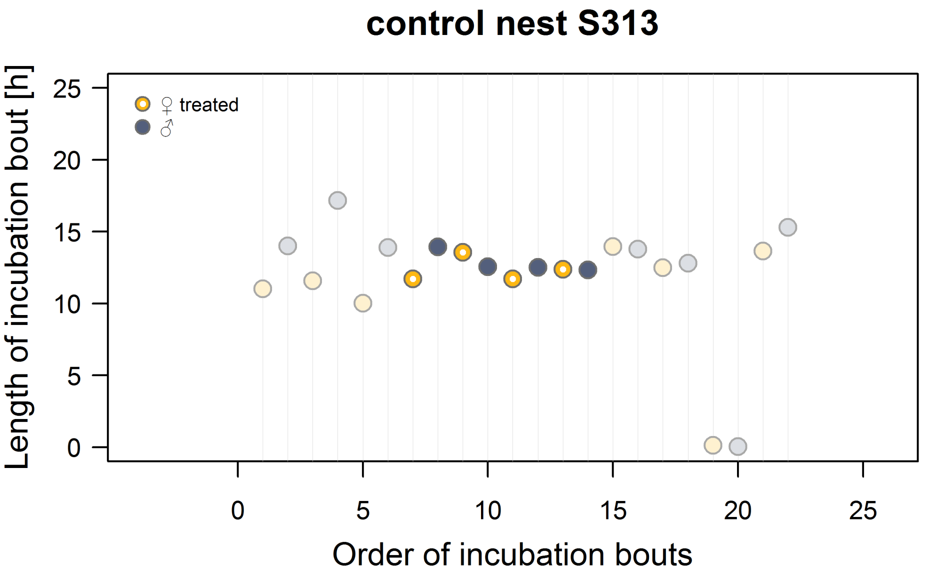

control nest S313
♀ treated
♂
Length of incubation bout [h]
0
5
10
15
20
25
Order of incubation bouts
0
5
10
15
20
25

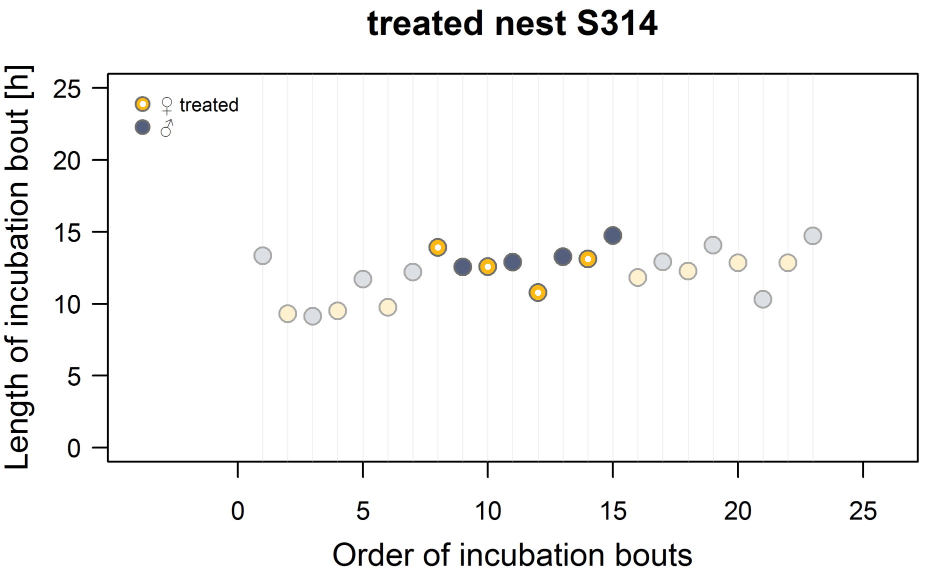
treated nest S314
♀ treated
♂
Length of incubation bout [h]
0
5
10
15
20
25
Order of incubation bouts
0
5
10
15
20
25

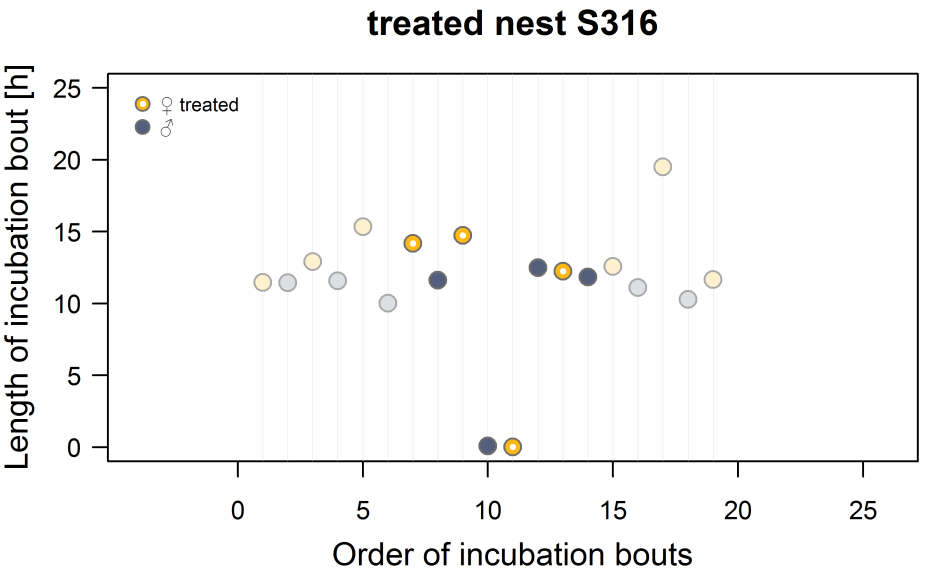
treated nest S316
♀ treated
♂
Length of incubation bout [h]
0
5
10
15
20
25
Order of incubation bouts

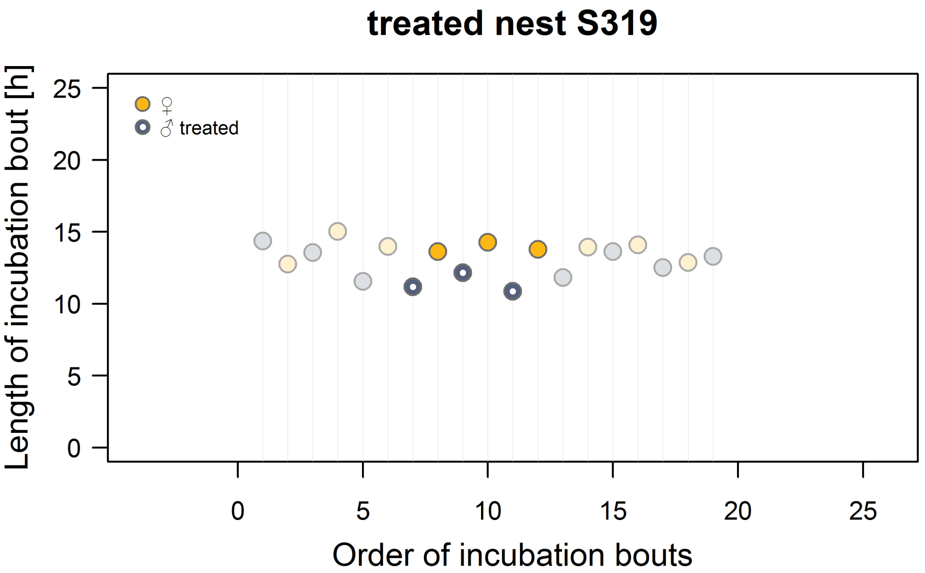
treated nest S319
♀
♂ treated
Length of incubation bout [h]
0
5
10
15
20
25
Order of incubation bouts
0
5
10
15
20
25

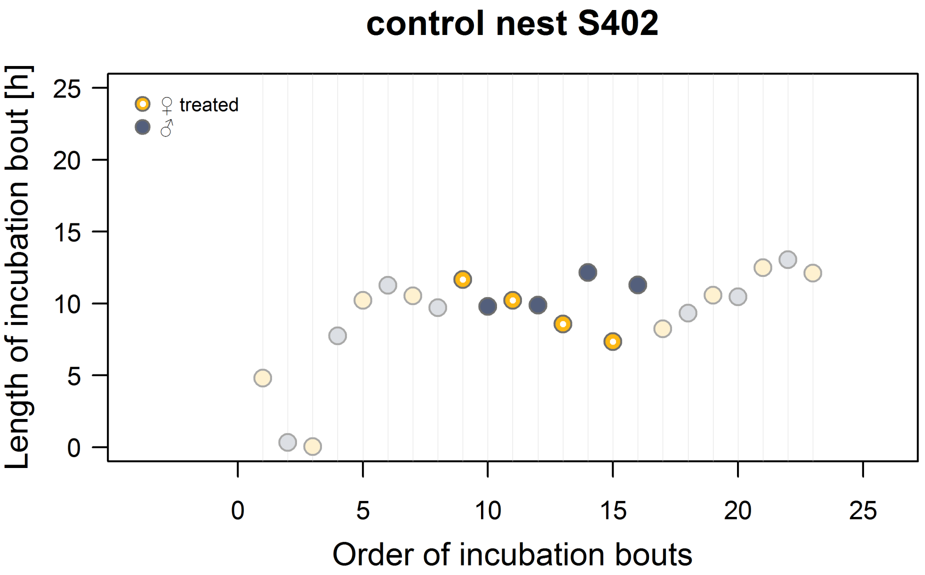

control nest S402
♀ treated
♂
Length of incubation bout [h]
Order of incubation bouts
0
5
10
15
20
25

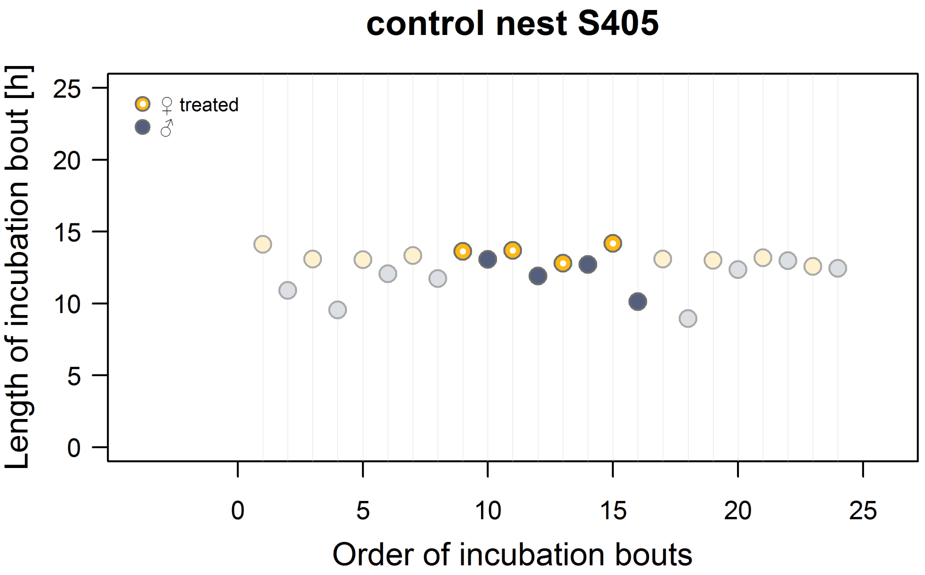

control nest S405
♀ treated
♂
Length of incubation bout [h]
0
5
10
15
20
25
Order of incubation bouts
0
5
10
15
20
25

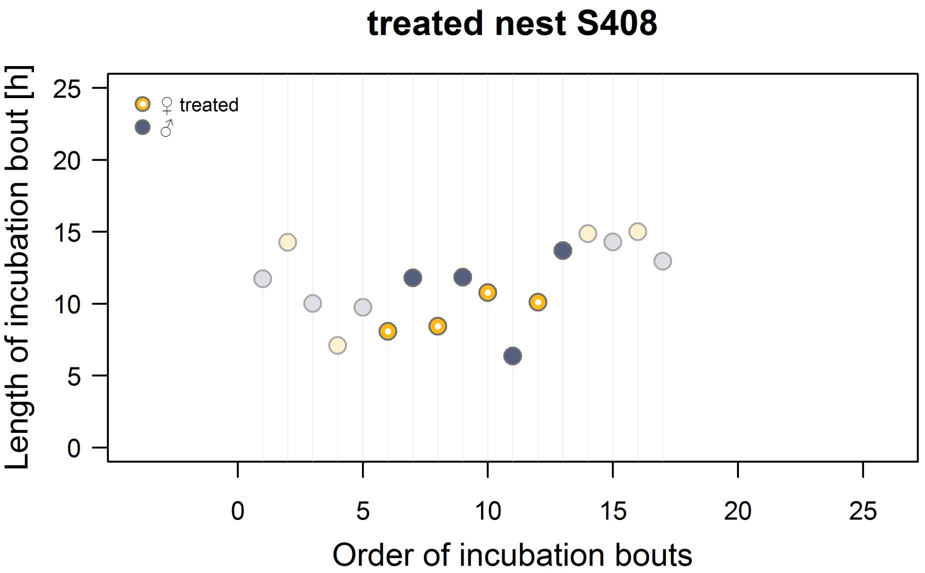

treated nest S408
♀ treated
♂
Length of incubation bout [h]
0
5
10
15
20
25
0
5
10
15
20
25
Order of incubation bouts

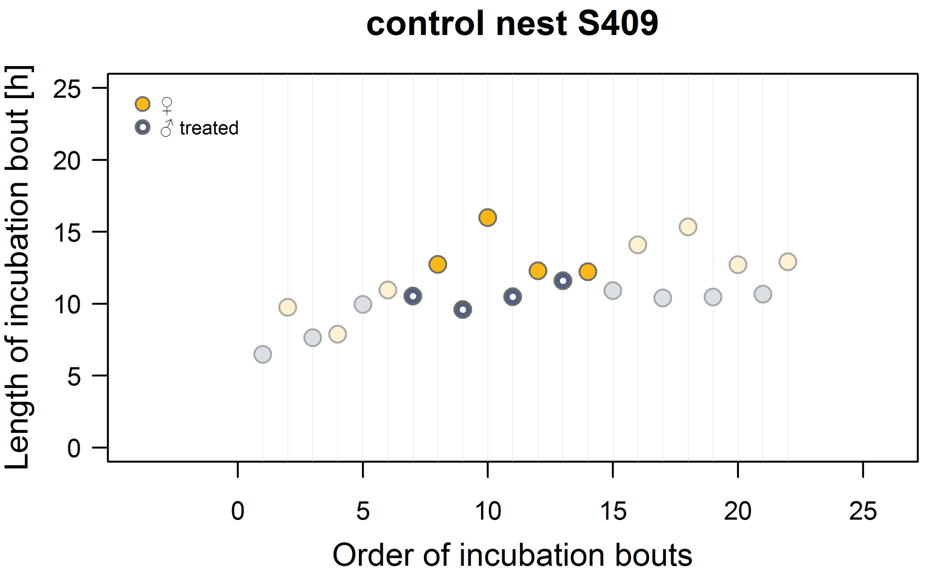
control nest S409
♀
♂ treated
Length of incubation bout [h]
Order of incubation bouts
0
5
10
15
20
25
0
5
10
15
20
25

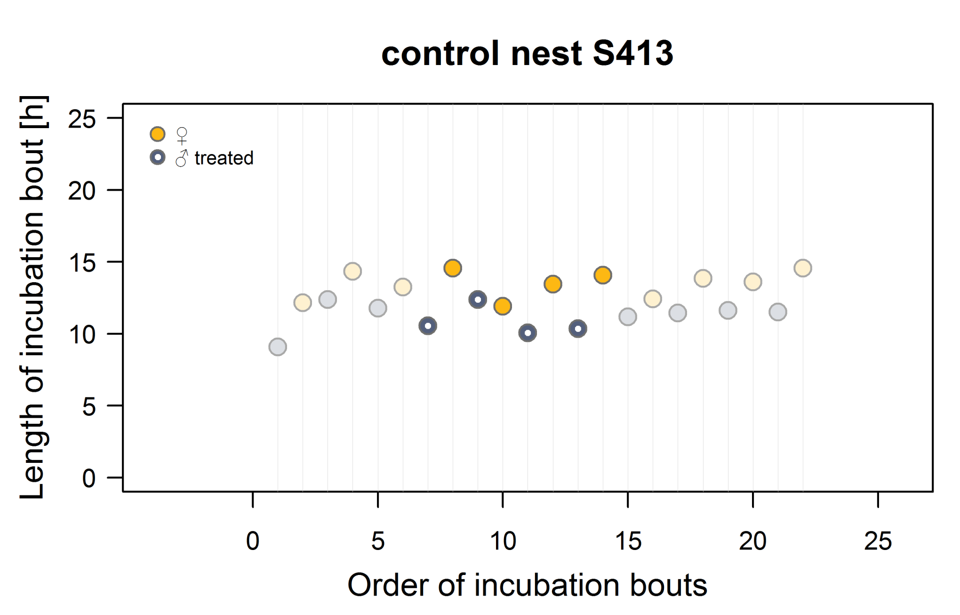

control nest S413
Length of incubation bout [h]
Order of incubation bouts
♀
♂ treated
0
5
10
15
20
25

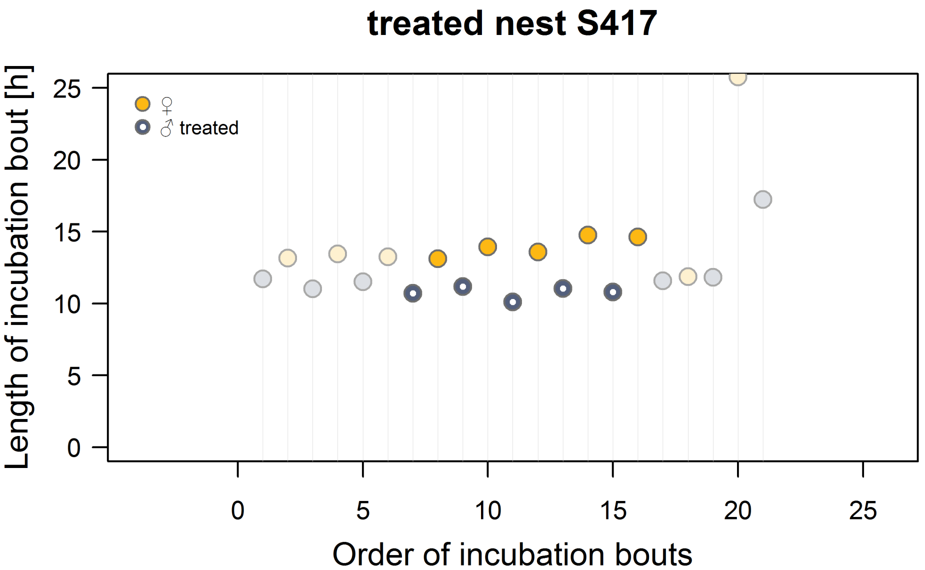

treated nest S417
♀
♂ treated
Length of incubation bout [h]
Order of incubation bouts
0
5
10
15
20
25

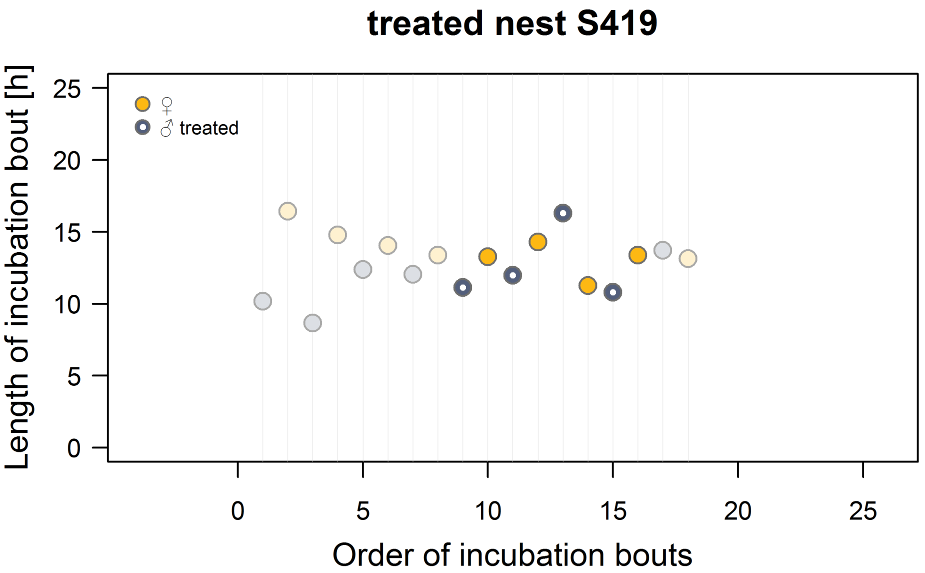

treated nest S419
♀
♂ treated
Length of incubation bout [h]
0
5
10
15
20
25
Order of incubation bouts
0
5
10
15
20
25

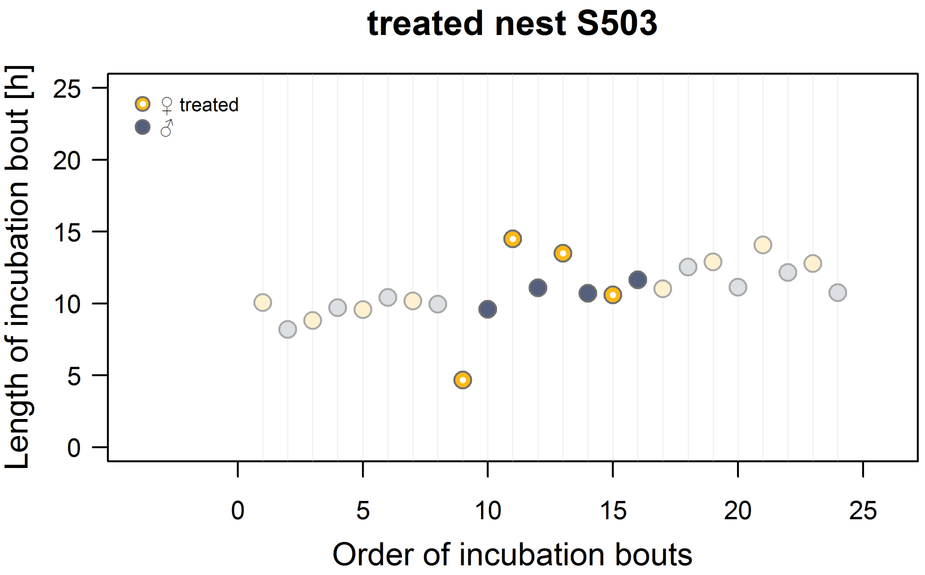
treated nest S503
♀ treated
♂
Length of incubation bout [h]
Order of incubation bouts
0
5
10
15
20
25

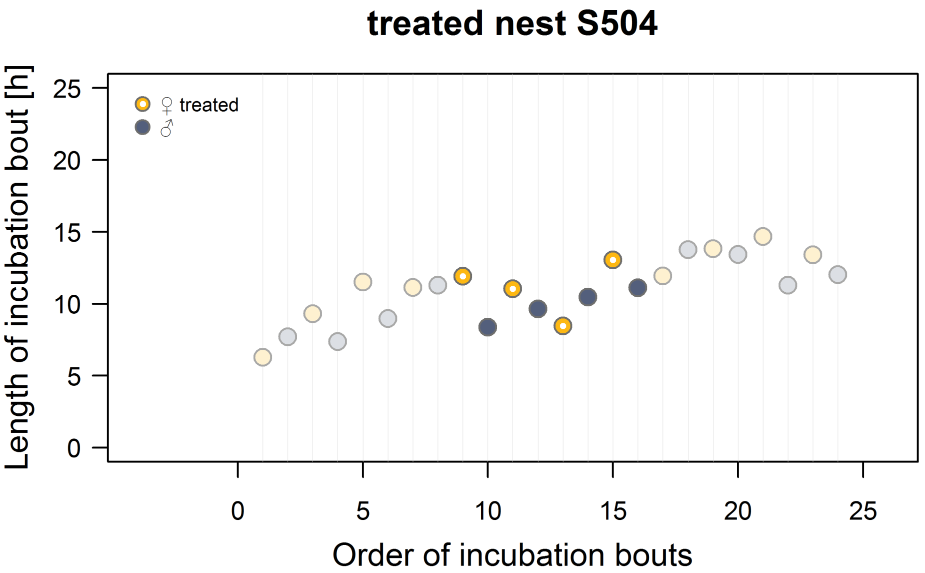

treated nest S504
♀ treated
♂
Length of incubation bout [h]
Order of incubation bouts
0
5
10
15
20
25
0
5
10
15
20
25

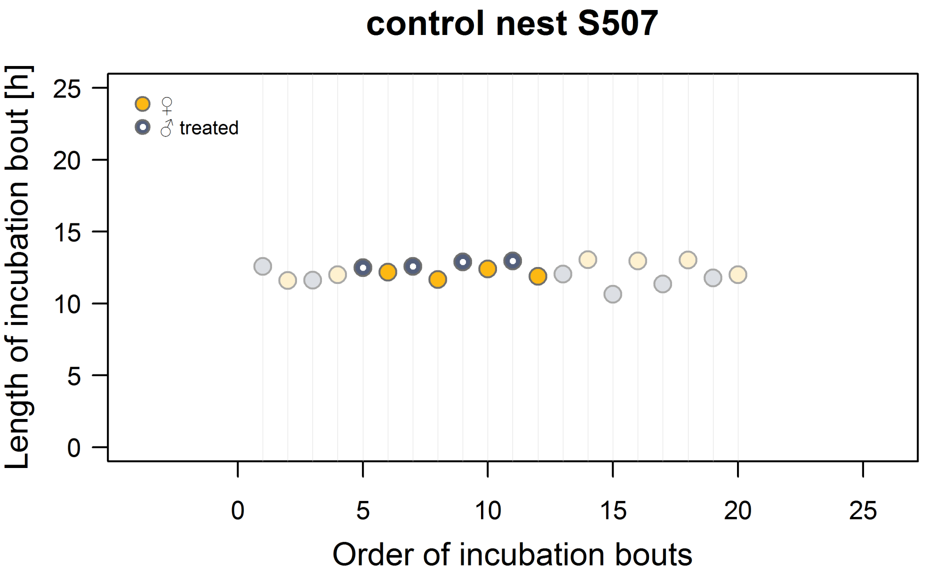

control nest S507
♀
♂ treated
Length of incubation bout [h]
Order of incubation bouts
0
5
10
15
20
25

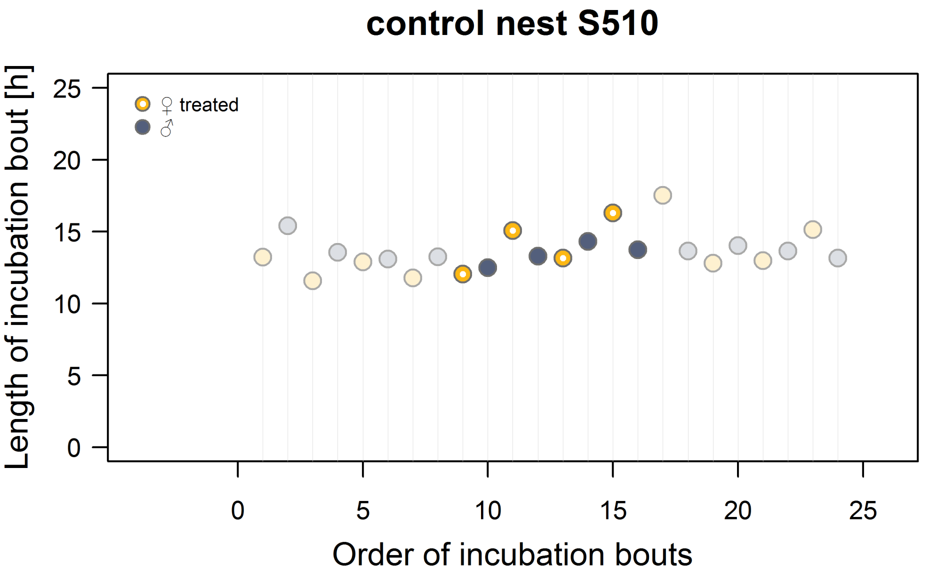
control nest S510
♀ treated
♂
Length of incubation bout [h]
Order of incubation bouts
0
5
10
15
20
25
0
5
10
15
20
25

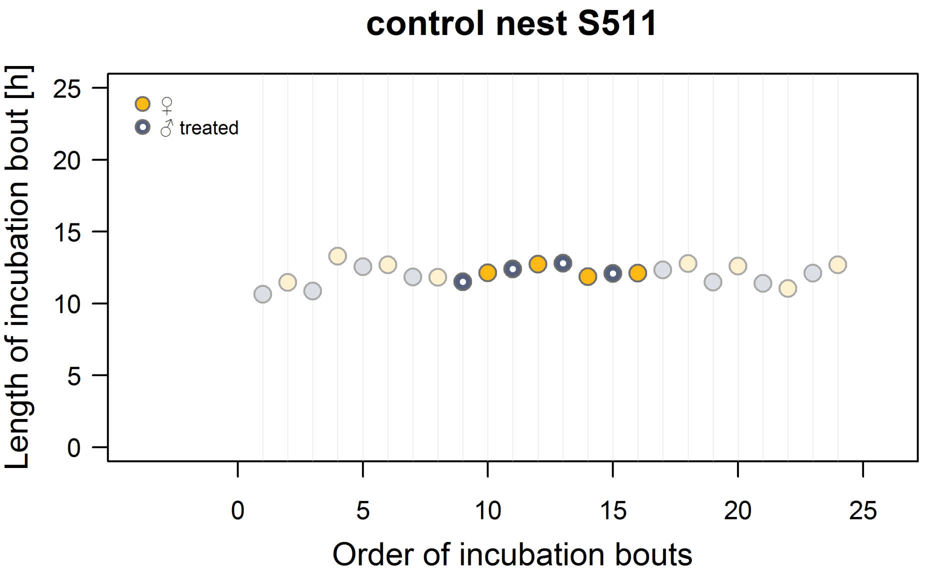
control nest S511
♀
♂ treated
Length of incubation bout [h]
Order of incubation bouts
0
5
10
15
20
25
0
5
10
15
20
25

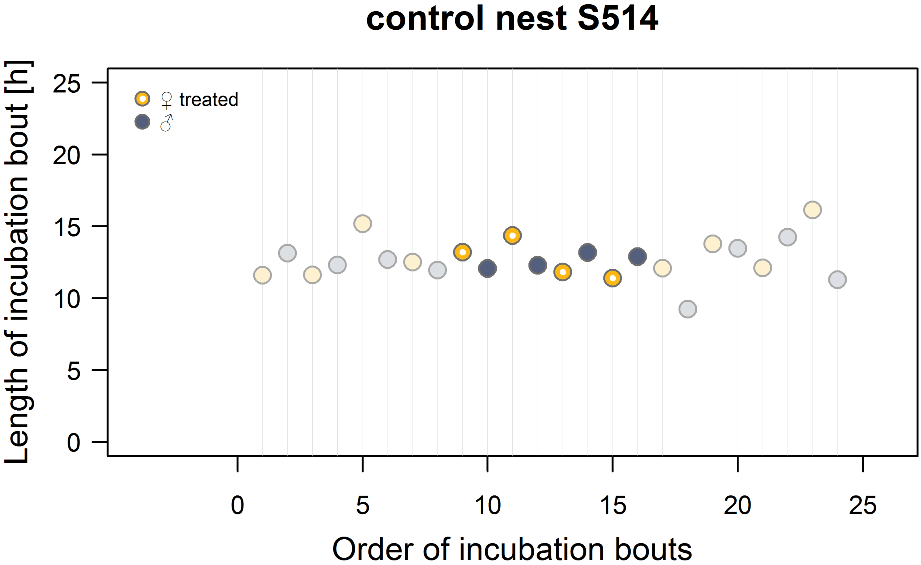

control nest S514
♀ treated
♂
Length of incubation bout [h]
Order of incubation bouts
0
5
10
15
20
25

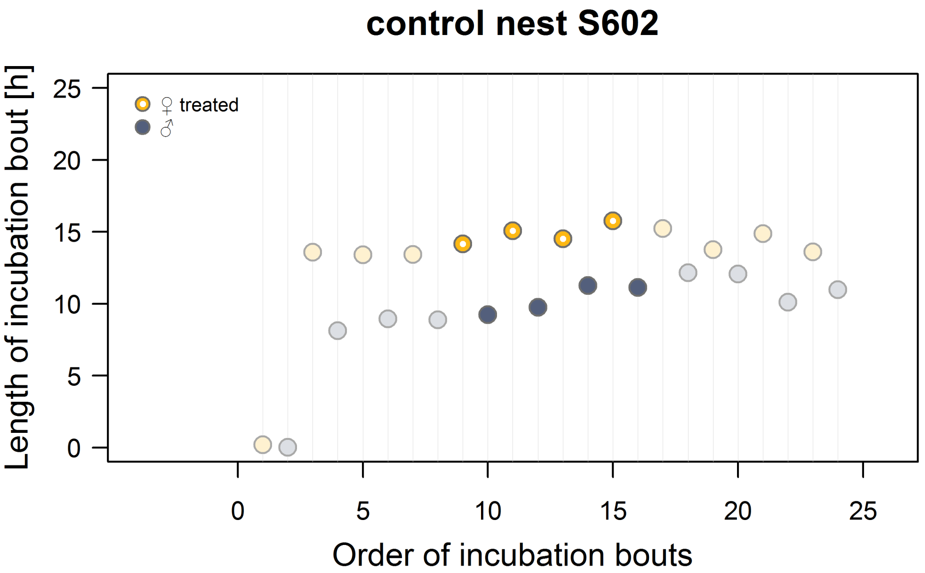
control nest S602
♀ treated
♂
Length of incubation bout [h]
0
5
10
15
20
25
Order of incubation bouts
0
5
10
15
20
25

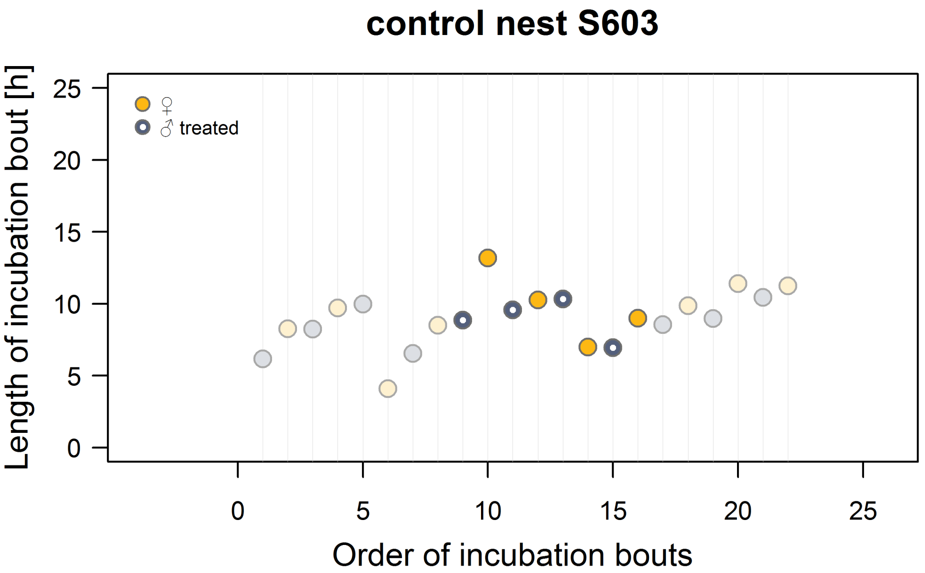

control nest S603
♀
♂ treated
Length of incubation bout [h]
0
5
10
15
20
25
Order of incubation bouts

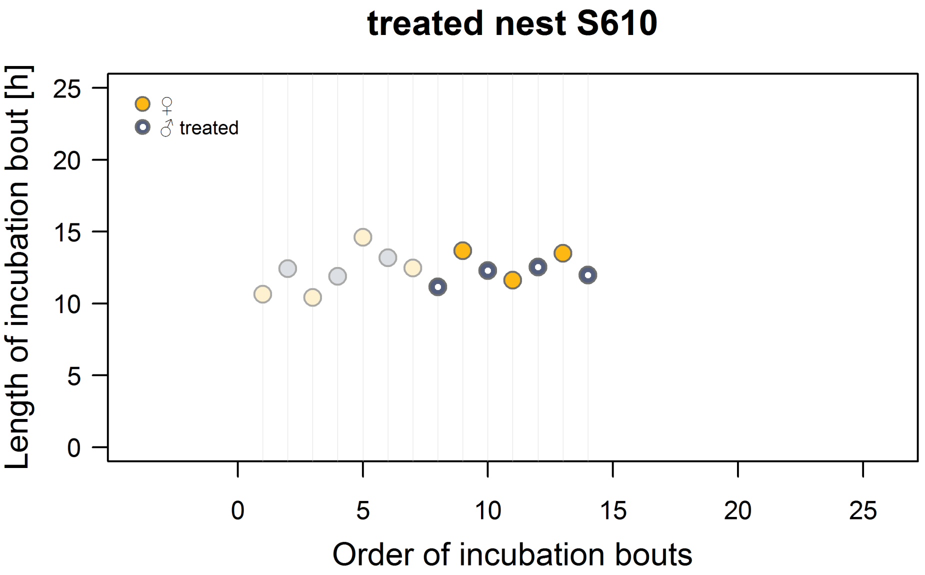

treated nest S610
♀
♂ treated
Length of incubation bout [h]
0
5
10
15
20
25
Order of incubation bouts
0
5
10
15
20
25

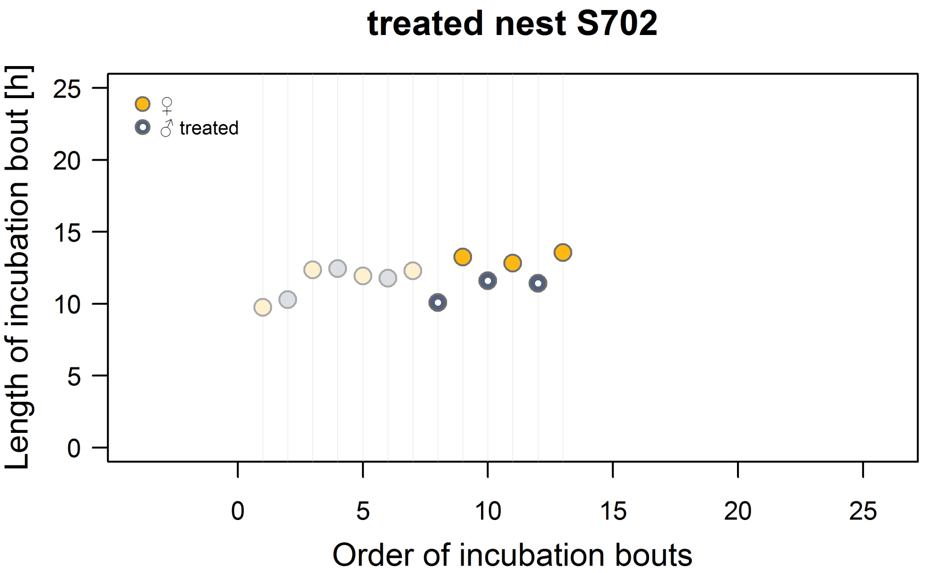
treated nest S702
♀
♂ treated
Length of incubation bout [h]
Order of incubation bouts
0
5
10
15
20
25
0
5
10
15
20
25

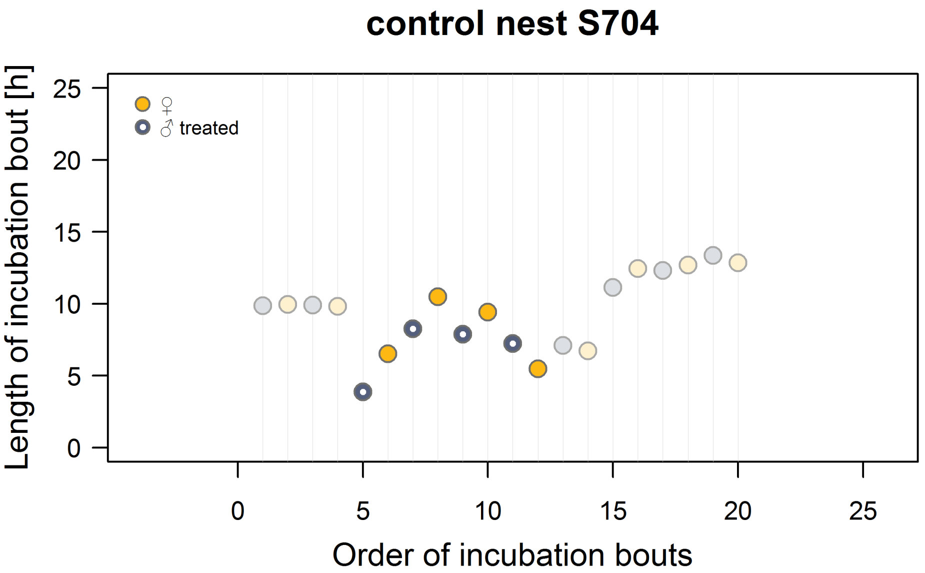
control nest S704
♀
♂ treated
Length of incubation bout [h]
Order of incubation bouts
0
5
10
15
20
25

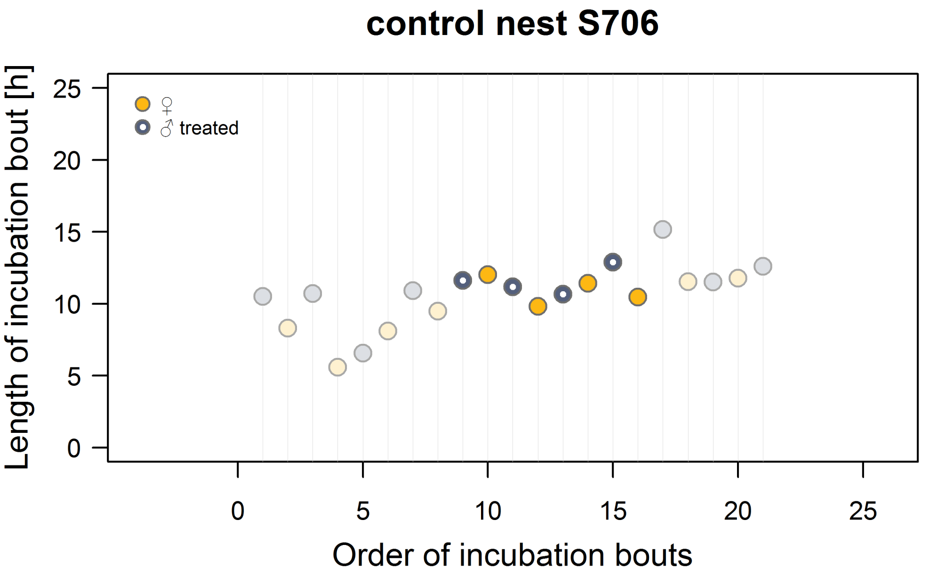
control nest S706
Length of incubation bout [h]
Order of incubation bouts
♀
♂ treated
0
5
10
15
20
25

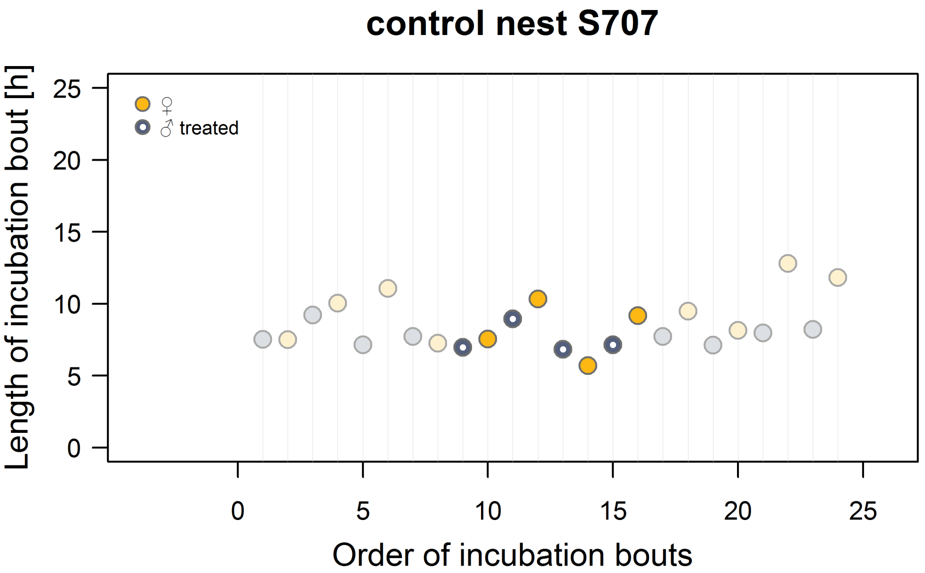

control nest S707
♀
♂ treated
Length of incubation bout [h]
Order of incubation bouts
0
5
10
15
20
25

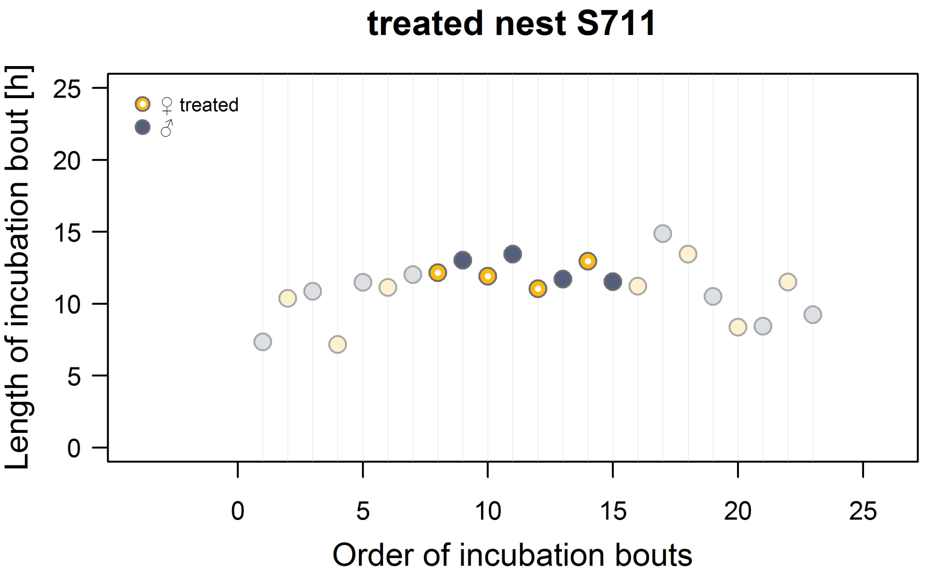
treated nest S711
♀ treated
♂
Length of incubation bout [h]
Order of incubation bouts
0
5
10
15
20
25
0
5
10
15
20
25

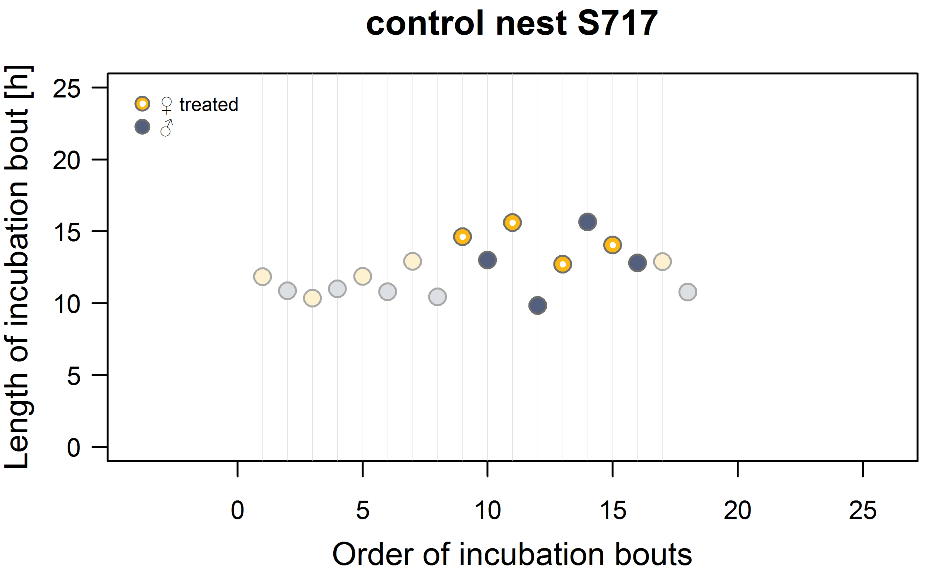

control nest S717
♀ treated
♂
Length of incubation bout [h]
0
5
10
15
20
25
Order of incubation bouts
0
5
10
15
20
25

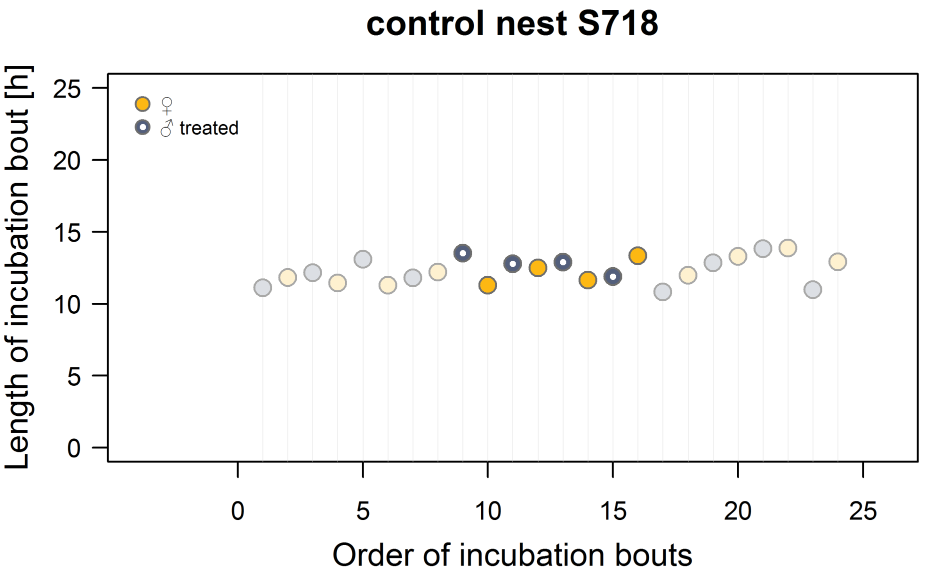
control nest S718
♀
♂ treated
Length of incubation bout [h]
Order of incubation bouts
0
5
10
15
20
25
0
5
10
15
20
25

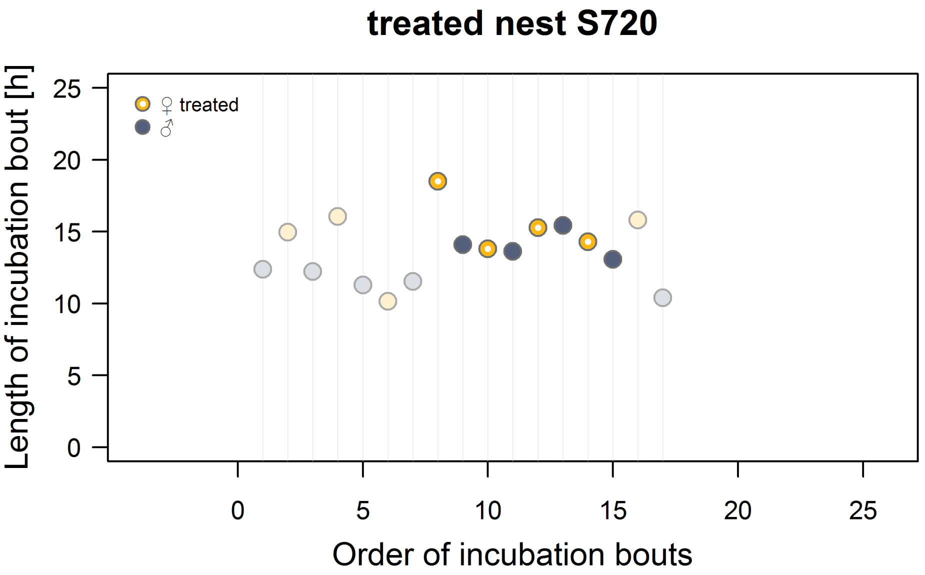

treated nest S720
♀ treated
♂
Length of incubation bout [h]
0
5
10
15
20
25
Order of incubation bouts
0
5
10
15
20
25

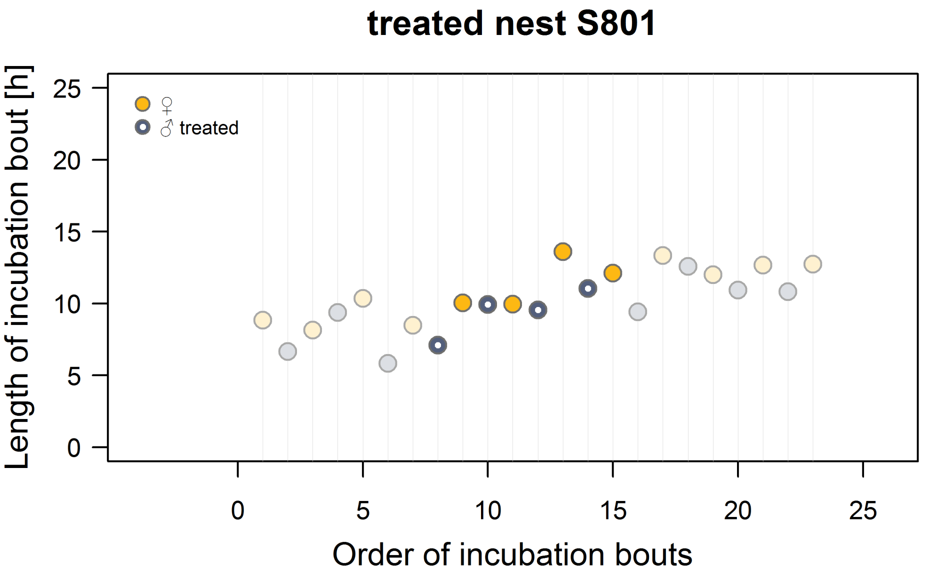

treated nest S801
♀
♂ treated
Length of incubation bout [h]
Order of incubation bouts
0
5
10
15
20
25

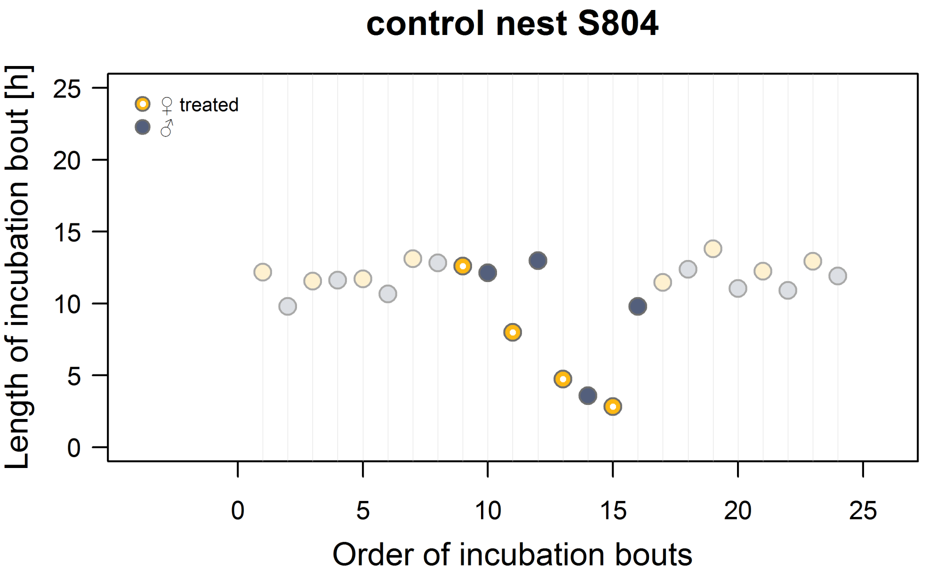

control nest S804
♀ treated
♂
Length of incubation bout [h]
25
20
15
10
5
0
0
5
10
15
20
25
Order of incubation bouts

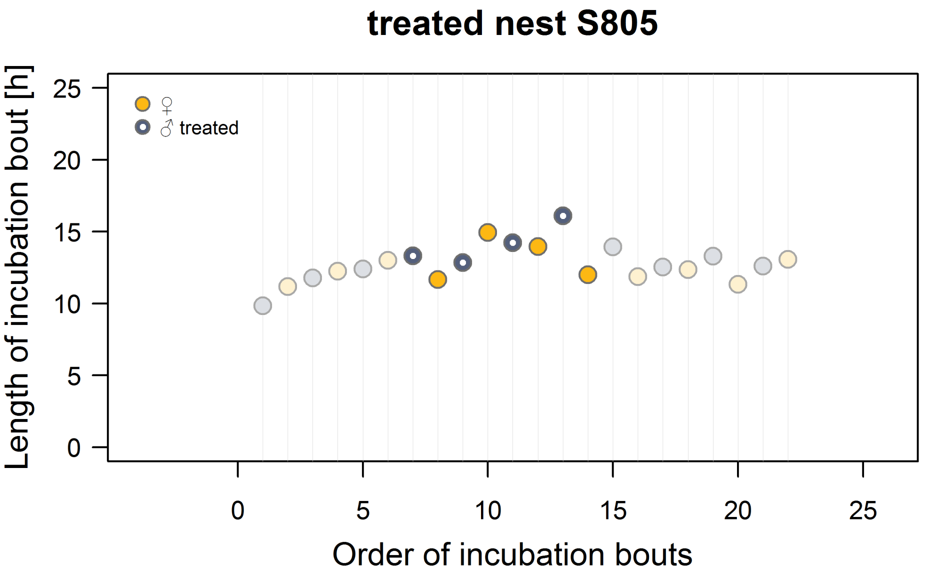

treated nest S805
♀
♂ treated
Length of incubation bout [h]
Order of incubation bouts
0
5
10
15
20
25

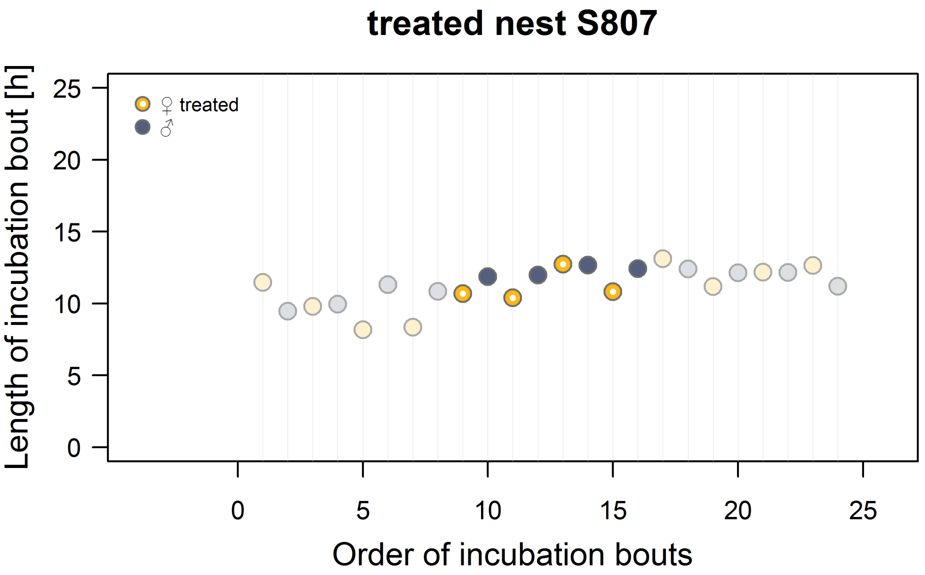

treated nest S807
Length of incubation bout [h]
25
20
15
10
5
0
♀ treated
♂
0
5
10
15
20
25
Order of incubation bouts

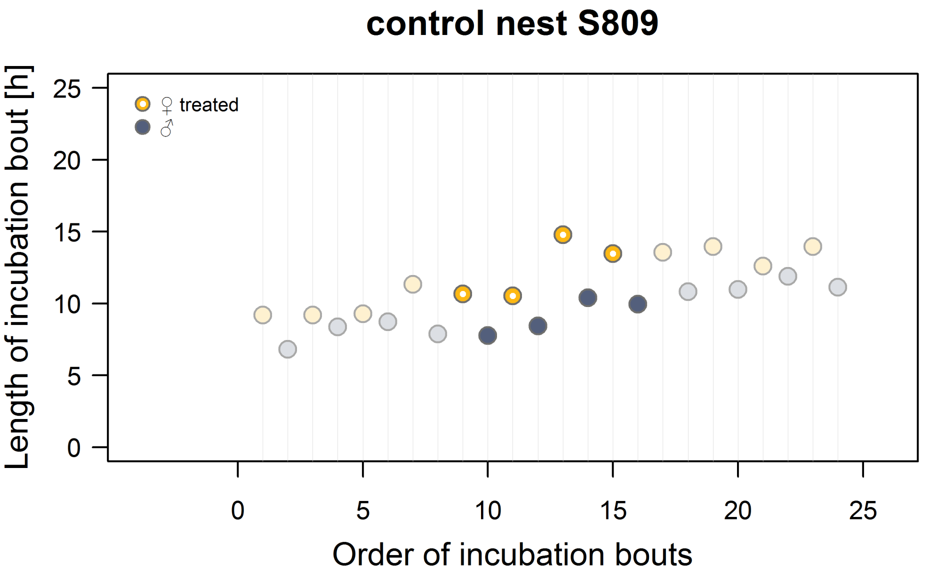

control nest S809
♀ treated
♂
Length of incubation bout [h]
0
5
10
15
20
25
Order of incubation bouts

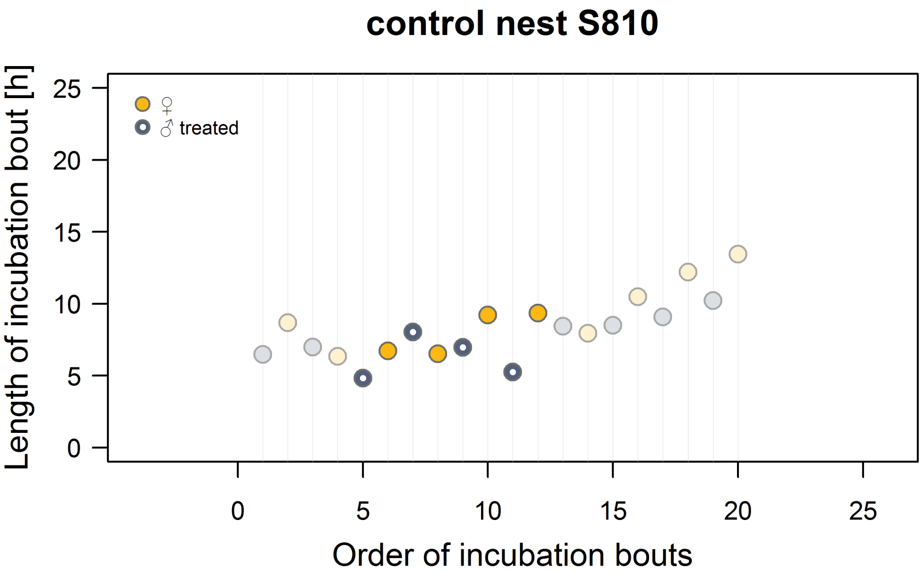

control nest S810
♀
♂ treated
Length of incubation bout [h]
0
5
10
15
20
25
0
5
10
15
20
25
Order of incubation bouts

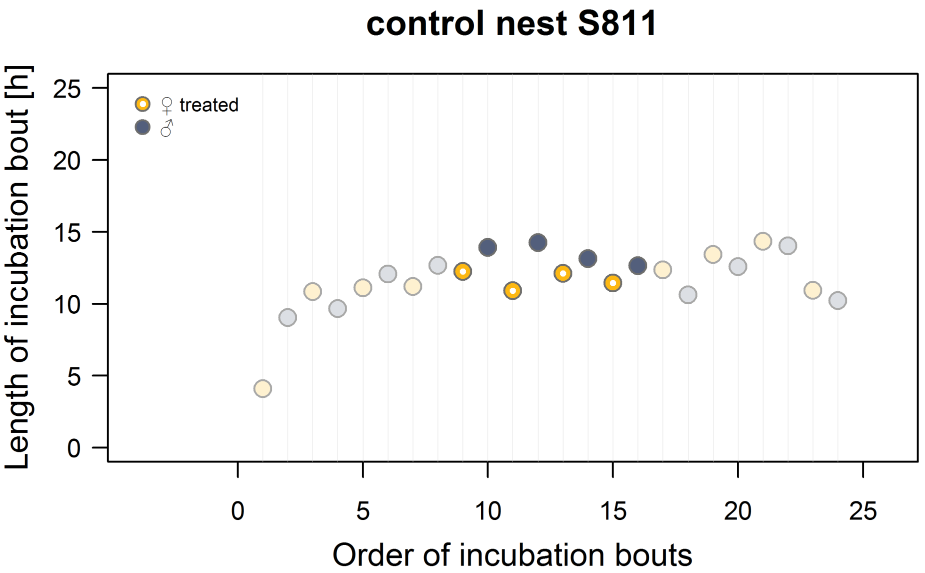
control nest S811
♀ treated
♂
Length of incubation bout [h]
Order of incubation bouts
0
5
10
15
20
25

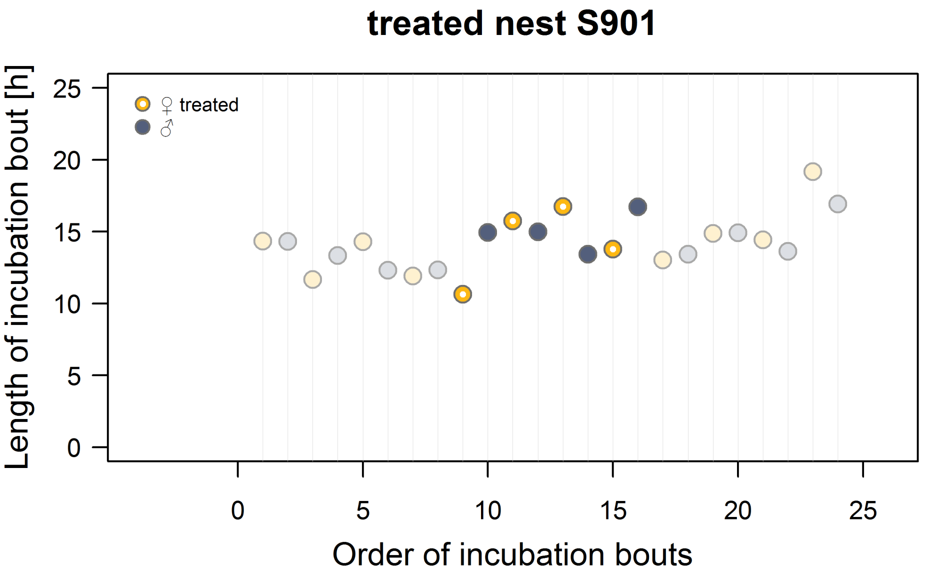

treated nest S901
♀ treated
♂
Length of incubation bout [h]
0
5
10
15
20
25
0
5
10
15
20
25
Order of incubation bouts
