# Supplementary material for: Biparental incubation-scheduling: no experimental evidence for major energetic constraints
Source: Behav Ecol. 2014 Sep 3;26(1):30–7. doi: 10.1093/beheco/aru156 (PMC4309980; doi:10.1093/beheco/aru156)
Supplement: Supplementary Data [file supp_aru156_Supplementary_1.pdf]

1. REANALYSIS OF THE INSULATION EXPERIMENT

There are four potential sources of bias and error in the original analysis of Cresswell et al. (2003) that we addressed with the reanalysis: unbalanced number of incubation bouts, lack of control for the period effects, unbalanced nest-pairs, and the use of means instead of the initial unit of measurement. Here we describe each issue separately and demonstrate how it contributed to the overestimation of the originally reported 55 min effect (95%CI: 11-99 min, t_{15} = 2.67, P = 0.017; Table S1).

Unbalanced number of incubation bouts

The matched-pair cross-over experimental design requires that each nest contains only control incubation bouts from within one of the 48 h experimental periods, but control bouts outside of these periods were included. Thus, in nests that first served as control and then were treated, bouts from before the 48 h control-period and from after the 48 h treatment period were included as control, whereas in nests that first were treated and then served as control, bouts from before the treatment period and from after the control period were included (see Supplementary 3).

We recalculated the mean bout lengths per nest and experimental period, excluding bouts that ended outside the first or started outside the second 48 h experimental period. Applying the same test (paired *t*-test) as in the original analysis to the new dataset reduced the treatment effect to 32.7 min (-9.7-75.1 min; paired *t*-test: t_{15} = 1.6, P = 0.12; Table S1 – model 1).

Lack of control for the period effects

The data were collected using a matched-pair cross-over experimental design, but analyzed as a typical matched-pair design (paired *t*-test). Such analysis did not explicitly account for period effects (i.e., whether control or treatment was applied in the first or second 48 h experimental period; an issue emphasized by Hills and Armitage 1979; Jones and Kenward 1989; Díaz-Uriarte 2002). The period effect might have been substantial because the bout lengths of semipalmated sandpipers increase over the incubation period (Ashkenazie and Safriel 1979; Cresswell et al. 2003; Bulla et al. 2014). Carryover effects (i.e., whether the after-treatment control periods were influenced by the treatment) might also bias the results, but the experimental design prohibits controlling for those.

Using the same dataset as above, but controlling for the period effect (by including it in the linear model; a method described by, e.g., Jones and Kenward 1989; Díaz-Uriarte 2002), further reduced the effect of treatment: 30.3 min (95% CI: -20.6-81.2 min, P = 0.32, N = 32 period nest-means from 16 nests; Table S1 – model 2; an alternative method to control for the period effect is described in Hills and Armitage 1979).

Unbalanced nest-pairs

The matched-pair cross-over experimental design also requires that each nest-pair contains one nest that first serves as control and then is treated (CT) and one nest that is first treated and then serves as control (TC), but Cresswell et al. (2003) included one nest-pair where both nests were CT. Because the incubation bouts prolong

over the incubation period (Ashkenazie and Safriel 1979; Cresswell et al. 2003; Bulla et al. 2014), this nest-pair likely led to an overestimate of the final treatment effect on the length of incubation bouts. Indeed, excluding this nest-pair from the analysis (presented above) decreased the treatment effect to 19.4 min (-20.7-59.4 min, P = 0.47, N = 28 period nest-means from 14 nests; Table S1 – model 3).

Table S1

Period and treatment estimates (in minutes) from models on nest-mean bout lengths for control and treatment.

Model	Type	*N*	48 h period (95% CI)	Treatment (95% CI)
Original study	Paired *t*-test	32 period nest-means; 16 nests		55.2 (11.2-99.3), t = 2.7, P = 0.02
1	Paired *t*-test	32 period nest-means[a]; 16 nests		32.7 (-9.7-75.1), t = 1.6, P = 0.12
2	Linear model	32 period nest-means[a]; 16 nests	19.4 (-31.6-70.3), t = 0.93, P = 0.63	30.3 (-20.6-81.2), t = 1.5, P = 0.32
3	Linear model	28 period nest-means[a]; 14 nests	8.4 (-31.7-48.5), t = 0.51, P = 0.88	19.4 (-20.7-59.4), t = 1.2, P = 0.47

[a] Period nest-means (recalculated) included only bouts that ended within the 1st or started within the 2nd 48 h experimental period.

Use of means instead of original measurements

Semipalmated sandpipers show large within-nest variation in the length of incubation bouts (SD is about 17% of median incubation bout length; Bulla et al. 2014). Because of this variation, the precision of the nest means depends on the number of incubation bouts used for their calculation. Because the original mean-based analysis did not control for the number of bouts used to calculate the nest means, the precision of the final result depends on how balanced the number of bouts between nests and treatment sessions was (see Supplementary 3; e.g., in 5 nests one of the period means was derived from only one incubation bout). Furthermore, the use of nest-means made controlling the models for some confounding variables of bout length (e.g., sex) impossible.

Thus, we used mixed-effect models with individual bout lengths (instead of nest means) as dependent variable. We controlled for period effects (by including the period in the model) and for other confounding effects by centering bout lengths within the nest, by including day of incubation (quadratic) as a random slope and nest ID as random intercept, and by including confounding variables that act on the level of the bout in the analysis. These are the results reported in the main text and Supplementary Table S8 and S9.

2. SEASONAL DIFFERENCES

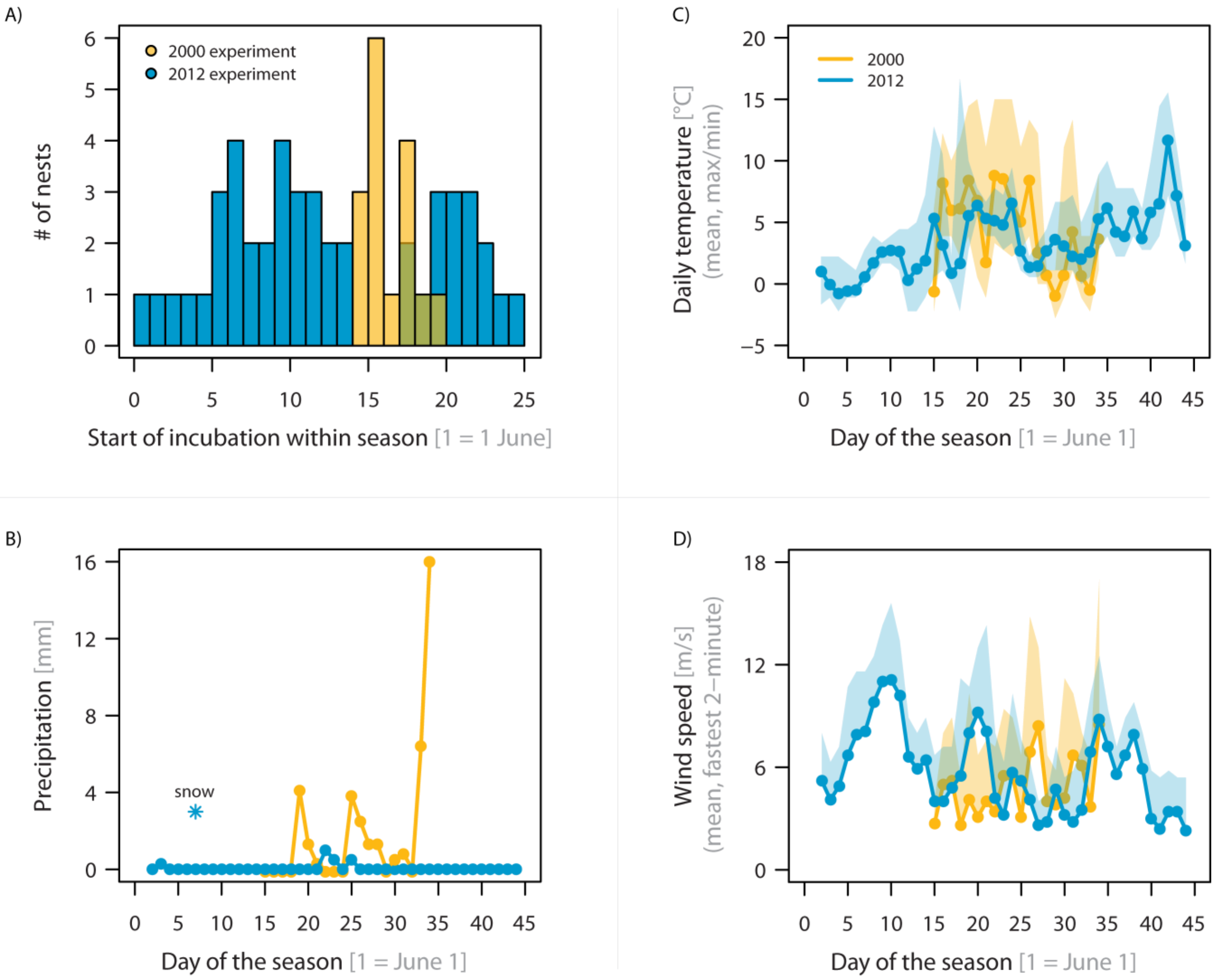

Figure S1

Between-year differences in the distribution of the start-date of incubation **A**, daily precipitation **B**, daily mean, minimum and maximum temperatures **C**, and daily mean and fastest 2 min wind speed **D**. The data for each year are limited to the period experienced by the nests prior or during the experiment (i.e., the period between the earliest start-date of incubation and the latest day of the experiment). The environmental data are freely available from NOAA: http://www.ncdc.noaa.gov/cdo-web/
and http://cdo.ncdc.noaa.gov/pls/plclimprod/poemain.accessrouter?datasetabbv=DS3505.

3. PICTURES

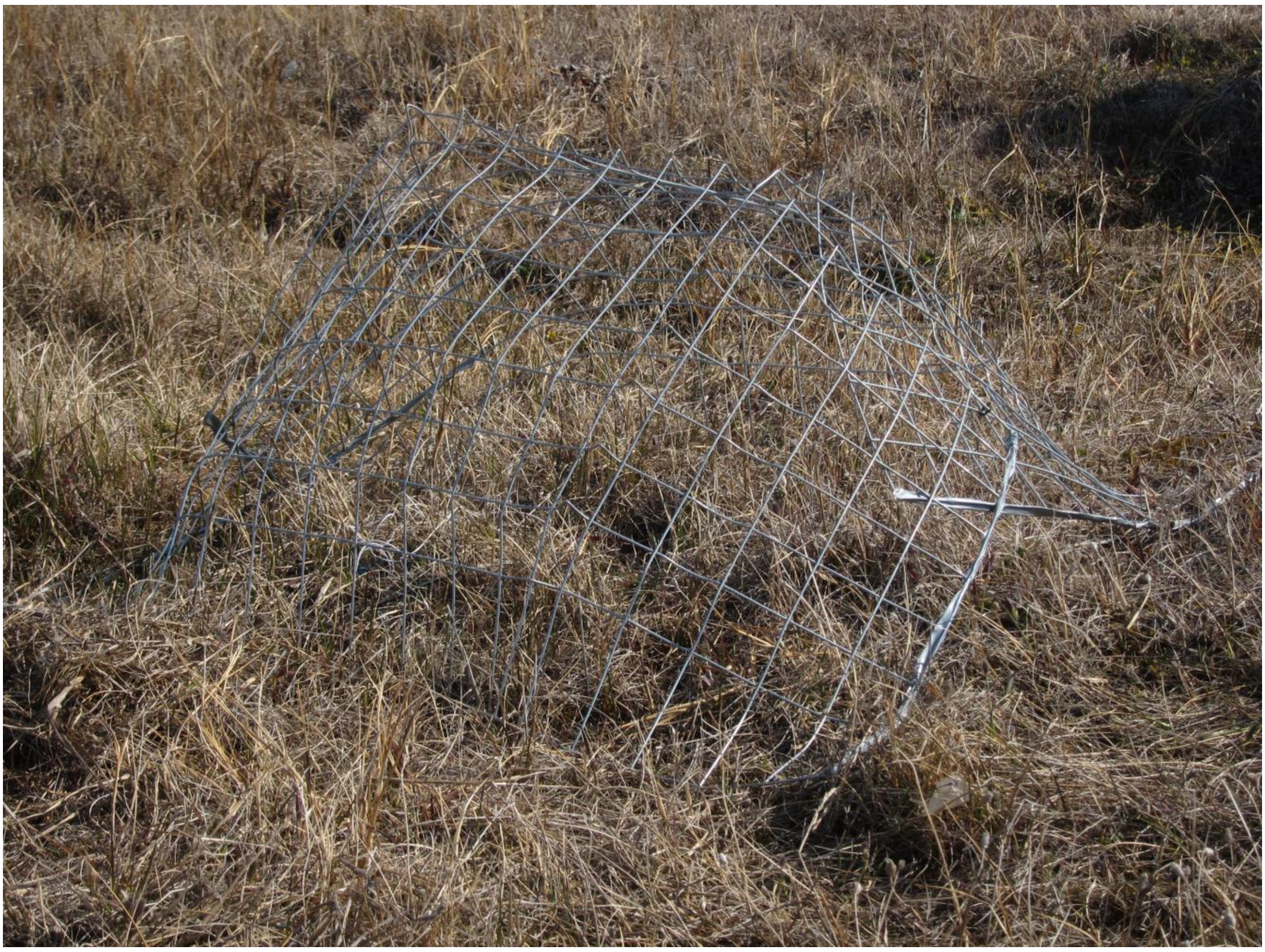

Picture S1

Exclosure that protected the nest against avian predators (approximate size 0.8 × 0.7 × 0.5 m; mesh size 5 × 5 cm and 5 × 10 cm where the cage touched the ground; wire ø 1.9 mm).

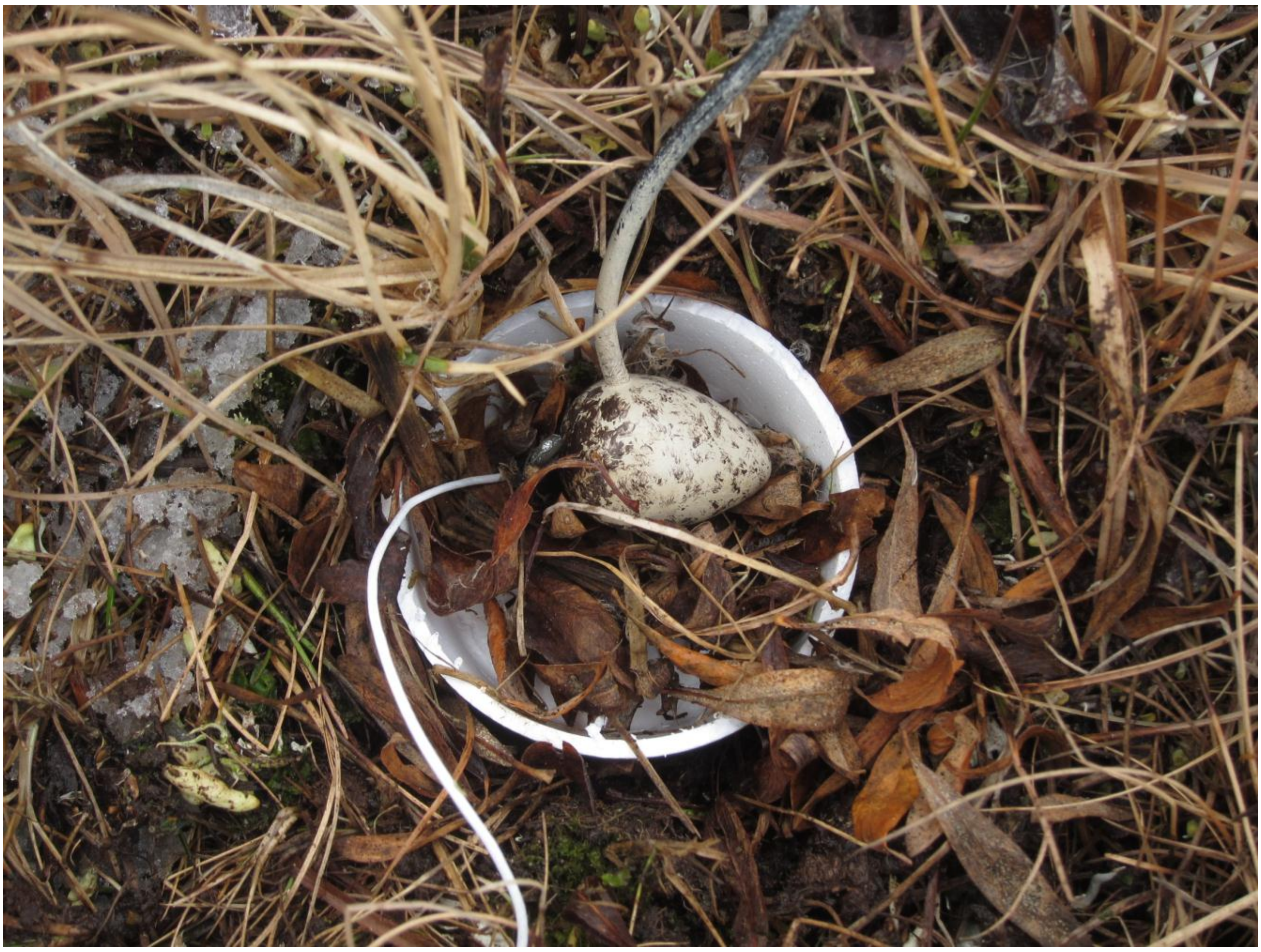

Picture S2

Artificial nest scrape with artificial egg and polystyrene insulation. The white cable connects the temperature probe with the data-logger.

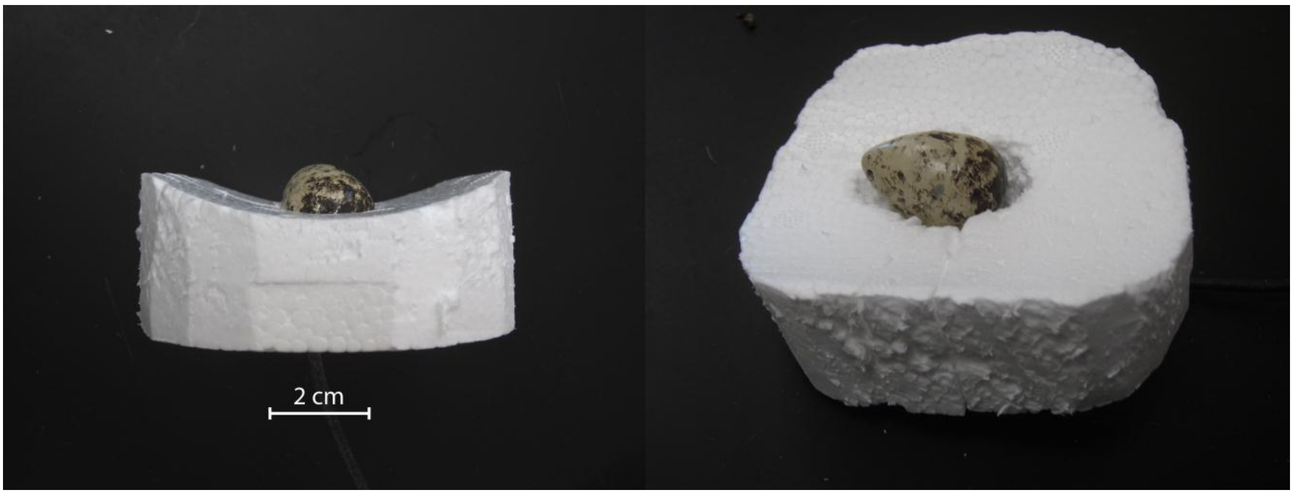

Picture S3

Artificial brood patch – heated egg embedded in polystyrene of approximately 9 × 8 × 2.5 cm.

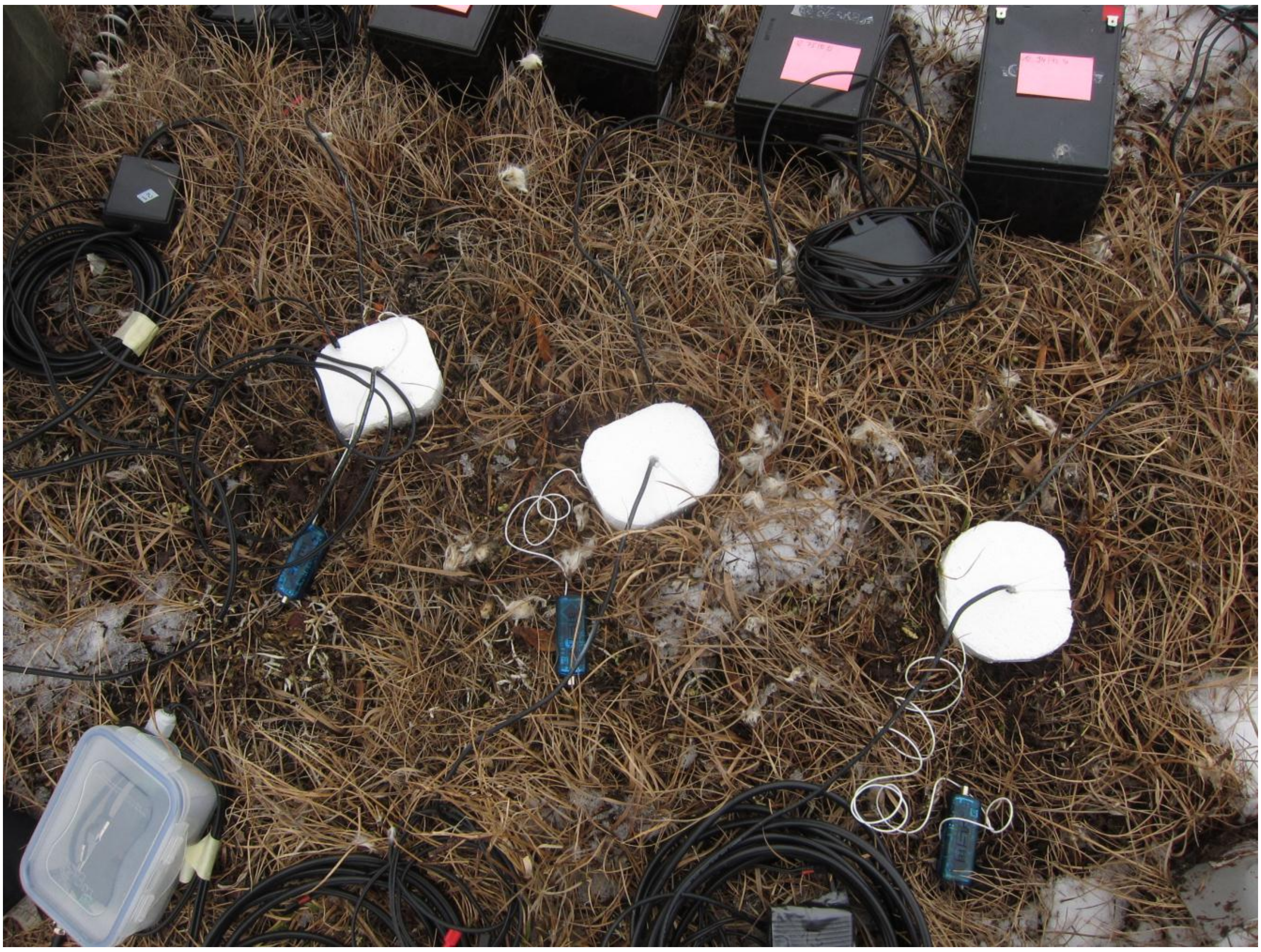

Picture S4

Artificial brood patches (white) covering the artificial nest scrapes.

4. A PRIORI POWER ANALYSES

In all power analyses the bout lengths were sampled from a normal distribution with mean bout length of 687 min (based on Bulla et al. 2014). The results of the power analyses are summarized in Figures S2-5. The statistical power for each comparison (each point in the graph) represents the proportion of correct findings ($P < 0.05$) from 1000 tests of 1000 randomly generated datasets.

In the first set of power analyses (Figure S2), each dataset was tested in two types of linear mixed-effect models, with bout length as dependent variable and nest as random intercept. In the first model, nest (control or treated) was entered in interaction with type of incubation bout (before, treated, or after); we only tested the difference of the "treated" bouts between control and treated nests, and the difference in slopes (treated bouts minus before bouts) in control and treated nests. In the second model, sex was entered in interaction with nest (control or treated) and type of incubation bout (before, treated, or after); we only tested the difference between male treated-bouts and female treated-bouts in treated nests.

Only the difference of the "treated" bouts between control and treated nests had sufficient statistical power with attainable sample sizes (Figure S2). Hence, the remaining three sets of power-analyses (Figure S3-S5) concerned only the treatment-assigned bouts ("treated") in control nests and treated (heated) bouts in treated nests. The analyses explored the effect of the number of repeats per period, ranging from 1 to 5 bouts of before, during and after treatment, as well as a scenario with 3 before, 4 treated and 3 after-treatment incubation bouts. Although 5 or more repeats would increase statistical power, their use would not be feasible because it would be difficult to fit the experiment within the 21-day incubation period, given that nests are found at various stages of incubation and that usually one or both birds have to be caught before the experiment can start.

Each dataset was tested in the linear mixed-effect model with bout length as dependent variable, nest (control or treated) in interaction with type of incubation bout (before, treated, or after) as a predictor, and nest as a random intercept. We only tested the difference in treated bouts in control and treated nests, and the difference in slopes (treated bouts minus before bouts) in control and treated nests; the figures display only the first comparison.

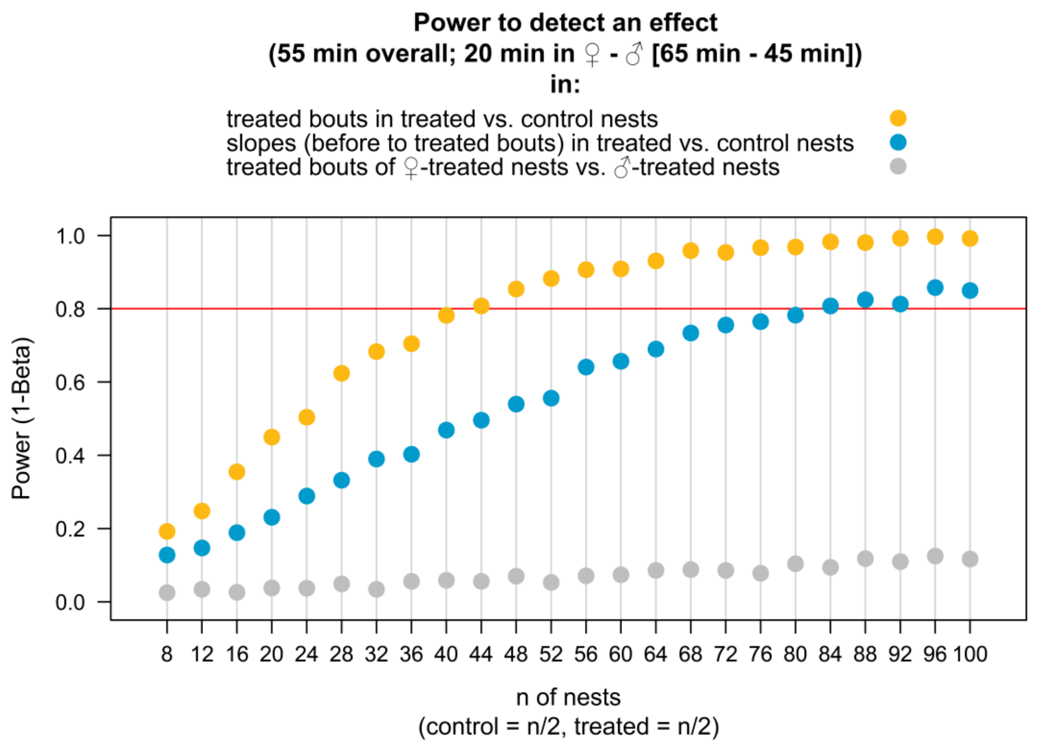

Figure S2 - Statistical power for the experiment in relation to sample size.

Depicted are the relationships between sample size and statistical power when "treated" bouts in control nests are compared with treated bouts in treated nests (yellow dots), when slopes (e.g., from before to treated bouts) in control nests are compared with slopes in treated nests (blue dots) or when treated bouts in female-treated nests are compared with treated bouts in male-treated nests (grey dots). Effect size was 65 min for females and 45 min for males (averaging 55 min – the effect reported in Cresswell et al. 2003), standard deviation (SD) in bout length was 118 min (based on the within-nest SD in bout length from Bulla et al. 2014), each bout type had 4 repeats within each nest (i.e., 4 before, 4 treated, 4 after bouts). The samples contained equal numbers of female-treated and male-treated nests. The red horizontal line indicates the acceptable recommended power of 0.8 (Cohen 1988).

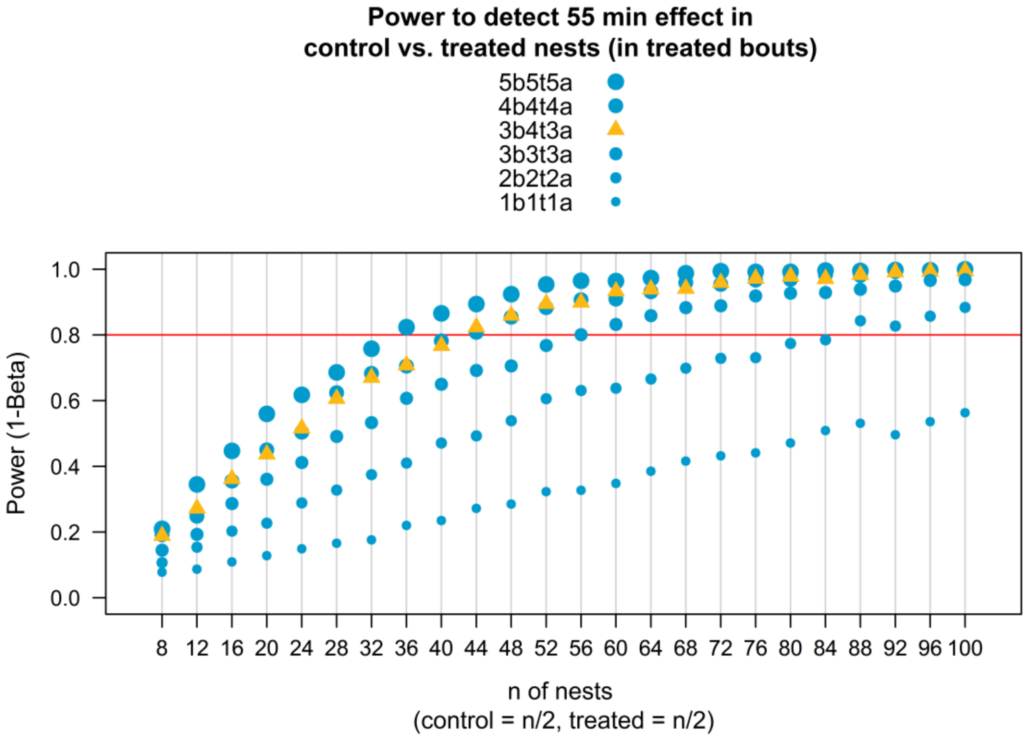

Figure S3 - Statistical power for the experiment in relation to sample size

Depicted are the relationships between sample size and statistical power for different experimental protocols (different symbols), differing in the number of before-treatment bouts (b), treated bouts (t) and after-treatment bouts (a). Effect sizes and bout length SD are similar as in Figure S2. The red horizontal line indicates the acceptable recommended power of 0.8 (Cohen 1988).

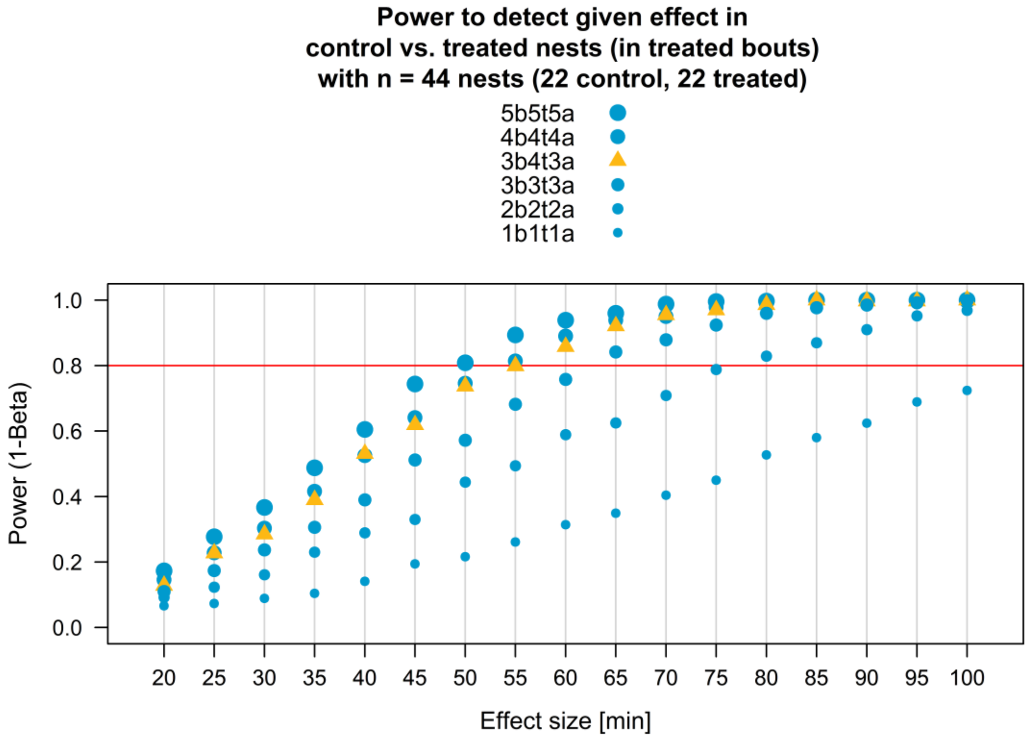

Figure S4 - Statistical power for the experiment in relation to effect size

Depicted are the relationships between effect size and statistical power for different experimental protocols (different symbols), differing in the number of before-treatment bouts (b), treated bouts (t) and after-treatment bouts (a). The results are based on a sample size of 22 control and 22 treated nests (based on Figure S2), and a SD in bout length of 118 min (based on the within-nest SD in bout length from Bulla et al. 2014). The red horizontal line indicates the acceptable recommended power of 0.8 (Cohen 1988).

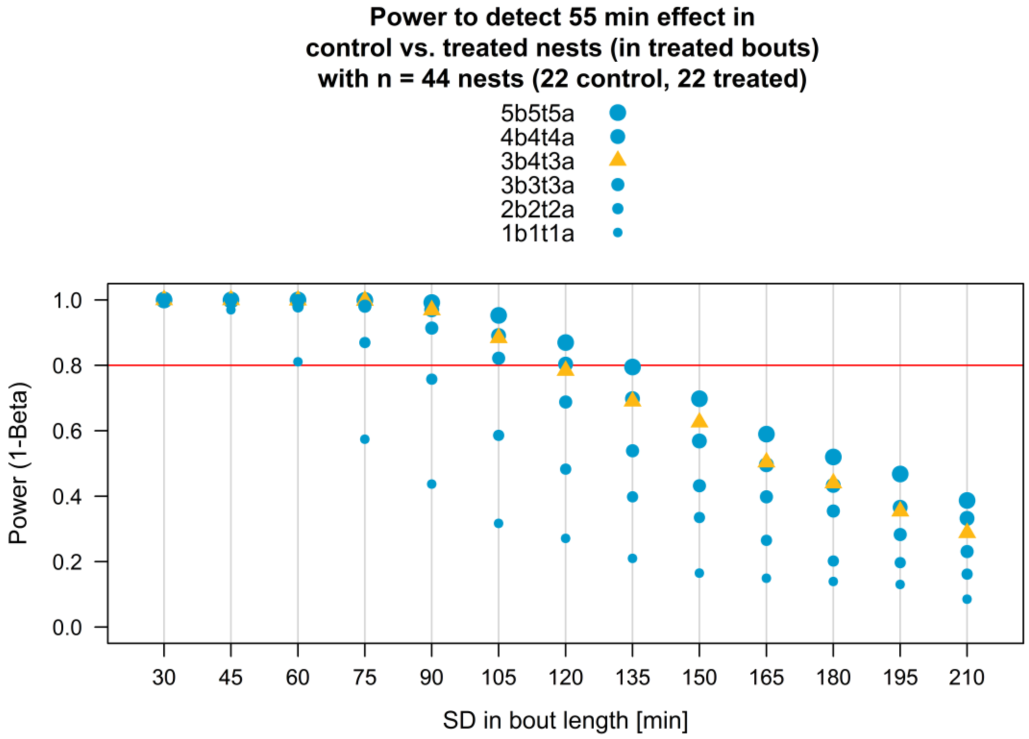

Figure S5 - Statistical power for the experiment in relation to the standard deviation (SD) in bout length.

Depicted are the relationships between SD in bout length and statistical power for different experimental protocols (different symbols), differing in the number of before-treatment bouts (b), treated bouts (t) and after-treatment bouts (a). The results are based on a sample size of 22 control and 22 treated nests (based on Figure S2), and an overall effect size of 55 min (based on Cresswell et al. 2003). The red horizontal line indicates the acceptable recommended power of 0.8 (Cohen 1988).

5. SAMPLE SIZES

Out of 99 nests equipped (at least for some time) with the incubation monitoring system, the following nests were excluded: nests with less than 4 recorded incubation bouts, nests where the monitoring system failed or nests used to test other experimental procedures (*N* = 30); control nests with less than 9 days of recorded incubation data (*N* = 12); treated nests where the treatment failed because a heated egg never turned on (*N* = 2), or because the nest was deserted by at least one parent or depredated (*N* = 9). This left 25 control nests (13 coded as female "treated", 12 as male "treated") and 21 treated nests (11 female-treated, 10 male-treated).

For the 46 nests used in the study, the following bouts were excluded: bouts during which eggs were laid; bouts when the nest was deserted or depredated; bouts that ended within 6 h before the start of hatching; bouts that started 2 days after the estimated hatch date; bouts during which the incubation-monitoring system was installed at the nest (if at the same time catching took place and the off-nest bird, instead of the incubating [caught] bird, returned after catching, this "after catching" bout was also excluded); bouts during which the artificial egg was inserted in the nest. Additional bouts were excluded in 2 nests with only one tagged parent. In these nests, the heated egg was heating whenever the battery was connected and the focal bird sat on the nest. As a result, when connecting and disconnecting the battery, the heating might have started or ended in the middle of the incubation bout. These bouts together with the next bout (bout of the untreated partner) were also excluded. Finally, given the experimental set up, a maximum of 4 before-treatment bouts and 4 after-treatment bouts per individual were used.

The final dataset contained 976 incubation bouts from 46 nests (median [range] = 22 [10 - 24] incubation bouts per nest; 100 "treated" incubation bouts in control nests, 81 treated incubation bouts in treated nests). The distribution of the incubation bouts (Table S2) deviated from the ideal one (4 before, 4 treated and 4 after bouts), for following reasons. First, the treatment-assigned birds in all 25 control nests had always 4 "treated" bouts, but in 9 nests were assigned less than 4 before bouts because these nests were found later in the incubation period, and in 4 nests were assigned less than 4 after bouts because later only a single bird incubated (3 nests), or the nest was depredated (1 nest). Second, the treated birds in all 21 treated nests had always 3 or 4 before-treatment bouts, depending on whether the nest was found early or late in the incubation period, and whether the treated bird incubated when the artificial egg or heat-SD-card was inserted. The treated birds had less than 4 treated bouts, if the nest was depredated during treatment (2 nests) or if the system was accidentally turned off before the end of the treatment (1 nest). Similarly, the treated birds had less than 4 after-treatment bouts, if the nest was depredated (3 nests), deserted (1 nest), later only a single bird incubated (3 nests), or was found later in the incubation period and thus started hatching (3 nests).

The final dataset for incubation-constancy contained 952 incubation bouts from 45 nests (median [range] = 22 [10 - 24] incubation bouts per nest; 96 "treated" incubation bouts in control nests, 81 treated incubation bouts in treated nests; the distribution of incubation bouts is in Table S2). One control nest was excluded because it lacked incubation temperatures and thus constancy could not be calculated.

The final dataset for probability of detectable exchange gap contained 928 exchanges from 46 nests (median [range] = 21 [9 - 23] exchanges per nest; 100 exchanges following "treated" incubation bouts [of

treatment-assigned birds] in control nests, 80 exchanges following treated incubation bouts [of treated birds] in treated nests).

The final dataset for duration of detectable exchange gaps **(≥5 s)** contained 655 detectable exchange gaps from 44 nests (median [range] = 15 [3 - 23] detectable exchange gaps per nest; 71 detectable exchange gaps following "treated" incubation bouts [of treatment-assigned birds] in control nests, 55 exchanges following treated incubation bouts [of treated birds] in treated nests).

Table S2

Distribution (number of nest with given number) of incubation bouts.

**Bout type**	**Before**			**Treated**				**After**				
Number of bouts	2	3	4	1	3	4	5	0	1	2	3	4
Control nest, "treated" assigned bird	3	6	16[a]			25[a]			2		2	21[a]
Control nest, untreated bird	3	6	16[a]			25[a]		1	1	1	3	19[a]
Treated nest, treated bird	1	12	8	1	2	16	2[b]	3	3	1	3	11
Treated nest, untreated bird		6	15	1	3	15	2[b]	4	2	4	1	10

[a]The dataset for incubation-constancy contained one nest less.

[b]At two nests with only one tagged parent the heating ran until the battery was disconnected.

6. MODELS

Table S3

Mixed model estimates of incubation bout length (transformed[a]) in relation to whether the nest, parent, and incubation bout were treated (heated).

Fixed effects on bout length (min)	Relative to	Estimate	95% CI	*z*	*P*
(Intercept)		-4.1	(-39.3, 31.1)		
Nest (treated)	control	1.9	(-51.8, 55.5)	0.1	1
Parent (treated)	untreated	6.2	(-43, 55.5)	0.35	1
Incubation bout (treated)	before	22.4	(-32.7, 77.6)	1.12	0.88
Incubation bout (after)	before	68.8	(7.2, 130.4)	3.08	0.02
Nest (treated) × Parent (treated)		-2.8	(-75.5, 69.9)	-0.11	1
Nest (treated) × Incubation bout (treated)		32.1	(-47.5, 111.7)	1.11	0.88
Nest (treated) × Incubation bout (after)		10.4	(-85.5, 106.2)	0.3	1
Parent (treated) × Incubation bout (treated)		5.5	(-61.7, 72.7)	0.23	1
Parent (treated) × Incubation bout (after)		-11.6	(-80.8, 57.7)	-0.46	1
Nest (treated) × Parent (treated) × Incubation bout (treated)		-27.3	(-127.3, 72.7)	-0.75	0.99
Nest (treated) × Parent (treated) × Incubation bout (after)		25.4	(-82, 132.9)	0.65	1

Random effects	Variance
Nest (intercept)	3301
Day of incubation (1st polynomial)	845782
Day of incubation (2nd polynomial)	1475503
Residual	13857

N = 976 incubation bouts from 46 nests (25 control, 21 treated); 100 “treated” incubation bouts (of treatment-assigned birds) in control nests, 81 treated incubation bouts (of treated birds) in treated nests. The model lacked major spatial and temporal auto-correlation, thus, unlike in Bulla et al. (2014), was not controlled for the length of the previous (partner’s) incubation bout.

[a] By subtracting for each individual the mean before-treatment bout length from all other incubation bout lengths.

Table S4

Mixed model estimates of incubation bout length (transformed[a]) in relation to whether the nest, parent, and incubation bout were treated (heated); model was controlled for disturbance[b], sex, and day of incubation (quadratic).

Fixed effects on bout length (min)	Relative to	Estimate	95% CI	*z*	*P*
(Intercept)		-1.8	(-59.5, 55.8)		
Disturbance		41.8	(16.4, 67.3)	4.75	<0.0001
Sex (male)	female	-14	(-35.6, 7.6)	-1.87	0.50
Day of incubation (1st polynomial)		139.9	(-804.7, 1084.4)	0.43	1
Day of incubation (2nd polynomial)		-800.2	(-1518.6, -81.9)	-3.21	0.02
Nest (treated)	control	-7.4	(-63.2, 48.4)	-0.38	1
Parent (treated)	untreated	8.1	(-42.8, 59)	0.46	1
Incubation bout (treated)	before	4.1	(-57.9, 66)	0.19	1
Incubation bout (after)	before	53.8	(-27.1, 134.7)	1.92	0.47
Nest (treated) × Parent (treated)		-3.6	(-78.5, 71.4)	-0.14	1
Nest (treated) × Incubation bout (treated)		45.3	(-37.3, 127.8)	1.58	0.72
Nest (treated) × Incubation bout (after)		35.8	(-65.8, 137.3)	1.02	0.98
Parent (treated) × Incubation bout (treated)		6.8	(-62.5, 76.1)	0.28	1
Parent (treated) × Incubation bout (after)		-11.2	(-82.9, 60.5)	-0.45	1
Nest (treated) × Parent (treated) × Incubation bout (treated)		-27.7	(-130.7, 75.3)	-0.78	1
Nest (treated) × Parent (treated) × Incubation bout (after)		27	(-83.8, 137.7)	0.7	1

Random effects		Variance
Nest (intercept)		3111
Day of incubation (1st polynomial)		852144
Day of incubation (2nd polynomial)		1358075
Residual		13418

N = 976 incubation bouts from 46 nests (25 control, 21 treated); 100 "treated" incubation bouts (of treatment-assigned birds) in control nests, 81 treated incubation bouts (of treated birds) in treated nests. The model lacked major spatial and temporal auto-correlation, thus, unlike in Bulla et al. (2014), was not controlled for the length of the previous (partner's) incubation bout.

[a] By subtracting for each individual the mean before-treatment bout length from all other incubation bout lengths.

[b] Disturbance (no = 0, yes = 1; continuous) was defined as in Bulla et al. (2014).

Table S5

Mixed model estimates of incubation-constancy within incubation bout (transformed[a]) in relation to whether the nest, parent, and incubation bout were treated (heated).

Fixed effects on constancy of incubation (%)	Relative to	Estimate	95% CI	*z*	*P*
(Intercept)		0.1	(-1.6, 1.8)		
Nest (treated)	control	0.1	(-2.4, 2.6)	0.08	1
Parent (treated)	untreated	0.1	(-2.4, 2.5)	0.06	1
Incubation bout (treated)	before	0.4	(-2.2, 3)	0.43	1
Incubation bout (after)	before	2.4	(-1.6, 6.4)	1.63	0.53
Nest (treated) × Parent (treated)		0	(-3.6, 3.5)	-0.04	1
Nest (treated) × Incubation bout (treated)		-0.4	(-4.2, 3.4)	-0.29	1
Nest (treated) × Incubation bout (after)		-2	(-8.3, 4.3)	-0.87	0.97
Parent (treated) × Incubation bout (treated)		-0.4	(-3.8, 2.9)	-0.37	1
Parent (treated) × Incubation bout (after)		0.7	(-2.7, 4.2)	0.59	1
Nest (treated) × Parent (treated) × Incubation bout (treated)		0.8	(-4, 5.7)	0.47	1
Nest (treated) × Parent (treated) × Incubation bout (after)		-1.3	(-6.6, 4)	-0.68	0.99

Random effects	Variance
Nest (intercept)	47
Day of incubation (1st polynomial)	58091
Day of incubation (2nd polynomial)	9862
Residual	32

N = 952 incubation bouts from 45 nests (24 control, 21 treated); 96 “treated” incubation bouts (of treatment-assigned birds) in natural nests, 81 treated incubation bouts (of treated birds) in treated nests. The model lacked spatial and temporal auto-correlation.

[a] By subtracting for each individual the mean before-treatment incubation constancy from all other incubation constancies.

Table S6

Mixed model estimates of incubation-constancy within incubation bout (transformed[a]) in relation to whether the nest, parent, and incubation bout were treated (heated); model was controlled for disturbance[b], sex, and length of incubation bout.

Fixed effects on constancy of incubation (%)	Relative to	Estimate	95% CI	*z*	*P*
(Intercept)		-6.5	(-9.6, -3.5)		
Disturbance		-0.6	(-1.8, 0.7)	-1.31	0.87
Sex (male)	female	0.1	(-0.9, 1.1)	0.28	1
Length of incubation bout		0.01	(0.007, 0.01)	8.11	<0.0001
Nest (treated)	control	-0.2	(-2.8, 2.3)	-0.26	1
Parent (treated)	untreated	0.3	(-2.1, 2.7)	0.35	1
Incubation bout (treated)	before	-0.2	(-2.7, 2.4)	-0.2	1
Incubation bout (after)	before	1.4	(-2.7, 5.4)	0.95	0.98
Nest (treated) × Parent (treated)		-0.1	(-3.6, 3.5)	-0.06	1
Nest (treated) × Incubation bout (treated)		-0.4	(-4.2, 3.4)	-0.31	1
Nest (treated) × Incubation bout (after)		-1.5	(-7.8, 4.8)	-0.68	1
Parent (treated) × Incubation bout (treated)		-0.5	(-3.8, 2.8)	-0.46	1
Parent (treated) × Incubation bout (after)		0.8	(-2.7, 4.2)	0.64	1
Nest (treated) × Parent (treated) × Incubation bout (treated)		1.1	(-3.8, 6)	0.63	1
Nest (treated) × Parent (treated) × Incubation bout (after)		-1.6	(-6.9, 3.6)	-0.89	0.99

Random effects	Variance
Nest (intercept)	52
Day of incubation (1st polynomial)	66460
Day of incubation (2nd polynomial)	12366
Residual	30

N = 952 incubation bouts from 45 nests (24 control, 21 treated); 96 'treated' incubation bouts (of treatment-assigned birds) in natural nests, 81 treated incubation bouts (of treated birds) in treated nests. The model lacked spatial and temporal auto-correlation.

[a] By subtracting for each individual the mean before-treatment incubation constancy from all other incubation constancies.

[b] Disturbance (no = 0, yes = 1; continuous) was defined as in Bulla et al. (2014).

Table S7

Binomial mixed model estimates[a] of the presence of an exchange gap[b] (≥5 s) in relation to whether the nest and parent in the preceding incubation bout were treated (heated); model was controlled for disturbance[c], and day of incubation[d].

Fixed effects on presence of detectable exchange gap	Relative to	Estimate[a]	95% CI	*z*	*P*
(Intercept)		0.2	(-1, 1.5)		
Disturbance		-0.2	(-0.7, 0.4)	-0.8	0.99
Day of incubation		-0.6	(-1.2, 0.1)	-2.55	0.11
Nest (treated)	control	1	(-0.5, 2.6)	1.93	0.40
Parent (treated)	untreated	0.6	(-0.4, 1.7)	1.68	0.58
Incubation bout (treated)	before	0.9	(-0.3, 2.1)	2.14	0.27
Incubation bout (after)	before	1.3	(-0.3, 3)	2.26	0.21
Nest (treated) × Parent (treated)		-0.3	(-2, 1.4)	-0.48	1
Nest (treated) × Incubation bout (treated)		-0.2	(-1.8, 1.4)	-0.3	1
Nest (treated) × Incubation bout (after)		-0.4	(-2.4, 1.5)	-0.61	1
Parent (treated) × Incubation bout (treated)		-0.7	(-2.1, 0.8)	-1.33	0.84
Parent (treated) × Incubation bout (after)		-1.2	(-2.7, 0.3)	-2.25	0.22
Nest (treated) × Parent (treated) × Incubation bout (treated)		-0.2	(-2.5, 2.1)	-0.27	1
Nest (treated) × Parent (treated) × Incubation bout (after)		0.8	(-1.7, 3.3)	0.9	0.98

Random effects	Variance
Nest (intercept)	1.43
Day of incubation	0.13

N = 928 exchanges from 46 nests (25 control, 21 treated); 100 exchanges following “treated” incubation bouts (of treatment-assigned birds) in control nests, 80 exchanges following treated incubation bouts (of treated birds) in treated nests. Results for only specifically defined (simultaneously tested) hypotheses demonstrate no major effect of the heating on the occurrence of the exchange gap: the detectability of exchange gaps following the treated bouts in treated nests was higher than after the “treated” bouts in control nests (0.4, 95%CI: -0.9–1.6, z = 0.71, P = 0.82) – an effect in the opposite direction to that expected for exchange gaps following treated bouts; during treatment period, the occurrence of exchange gaps following the untreated bouts in treated nests (i.e., off-nest bouts of the treated bird) was higher than after the untreated bouts in control nests (0.9 [-0.4–2.2], z = 1.59, P = 0.26) – an effect in the opposite direction to that expected for exchange gaps following off-nest bouts of treated birds during treatment period; for comparison, during before-treatment period the occurrence of exchange gaps following the untreated bouts in treated nests was also higher than after the untreated bouts in control nests (1 [-0.2–2.3], z = 1.42, P = 0.14) – a similar effect to the (above described) effect during treatment period.

[a] On logit scale.

[b] Exchange gap (undetected = 0, detected = 1)

[c] Disturbance (no = 0, yes = 1; continuous) was defined as in Bulla et al. (2014).

[d] Day of incubation was mean centered.

Table S8

Mixed model estimates[a] of exchange gap duration (in minutes, ln transformed[b]) in relation to whether the nest and parent in preceding incubation bout were treated (heated); model was controlled for disturbance[c], and day of incubation[d].

Fixed effects on duration of detectable exchange gap	Relative to	Estimate[a]	95% CI	*z*	*P*
(Intercept)		-0.1	(-0.7, 0.6)		
Disturbance		0.1	(-0.2, 0.4)	0.99	0.96
Day of incubation		0	(-0.3, 0.4)	0.24	1
Nest (treated)	control	0	(-0.6, 0.7)	0.09	1
Parent (treated)	untreated	-0	(-0.6, 0.6)	-0.04	1
Incubation bout (treated)	before	-0.1	(-0.8, 0.6)	-0.24	1
Incubation bout (after)	before	-0.5	(-1.5, 0.4)	-1.55	0.67
Nest (treated) × Parent (treated)		0	(-0.8, 0.9)	0.04	1
Nest (treated) × Incubation bout (treated)		0	(-0.9, 0.9)	0.09	1
Nest (treated) × Incubation bout (after)		0.5	(-0.7, 1.7)	1.2	0.89
Parent (treated) × Incubation bout (treated)		0.2	(-0.7, 1)	0.53	1
Parent (treated) × Incubation bout (after)		0.4	(-0.5, 1.3)	1.22	0.88
Nest (treated) × Parent (treated) × Incubation bout (treated)		-0.4	(-1.6, 0.8)	-1.03	0.95
Nest (treated) × Parent (treated) × Incubation bout (after)		-0.5	(-1.8, 0.9)	-0.94	0.97

Random effects	Variance
Nest (intercept)	0.26
Day of incubation	0.15
Residual	1.36

N = 655 detectable exchange gaps (≥5 s) from 44 nests (24 control, 20 treated); 71 detectable exchange gaps following “treated” incubation bouts (of treatment-assigned birds) in control nests, 55 detectable exchange gaps following treated incubation bouts (of treated birds) in treated nests. Results for only specifically defined (simultaneously tested) hypotheses demonstrate no major effect of the heating on the duration of detectable exchange gaps: the detectable exchange gaps following the treated bouts in treated nests were shorter than the exchange gaps following the “treated” bouts in control nests (-0.4, 95%CI: -1–0.2, *z* = -1.47, *P* = 0.36) – a tendency for an effect in the direction to that expected for exchange gaps following treated bouts; during treatment period, the detectable exchange gaps following the untreated bouts in treated nests (i.e., following the off-nest bouts of the treated bird) were similar to the exchange gaps following the untreated bouts in control nests (0 [-0.6–0.7], *z* = 0.19, *P* = 1); for comparison, during before-treatment period the detectable exchange gaps following the untreated bouts in treated nests were similar to the exchange gaps following the untreated bouts in control nests (0 [-0.5–0.6], *z* = 0.09, *P* = 1).

[a] On ln-scale.

[b] By subtracting for each individual the mean before-treatment exchange gap from all other exchange gaps.

[c] Disturbance (no = 0, yes = 1; continuous) was defined as in Bulla et al. (2014).

[d] Day of incubation was mean centered.

Table S9

Mixed model estimates of incubation bout length (mean-centered within individual) in relation to period (first or second 48 h of the experiment) and whether the incubation bout was treated (insulated).

Fixed effects on bout length (min)	Relative to	Estimate	95% CI	*z*	*P*
(Intercept)		-5.2	(-39.3, 29)		
Period (2nd)	1st	16.8	(-20.6, 54.2)	1.05	0.57
Incubation bout (treated)	untreated	2.7	(-34.7, 40.1)	0.17	1
**Random effects**		**Variance**			
Nest (Intercept)		8			
Day of incubation (1st polynomial)		1922			
Day of incubation (2nd polynomial)		26248			
Residual		4842			

N = 87 incubation bouts from 14 nests from 7 nest-pairs; 42 treated bouts, 45 untreated bouts. The model lacked major temporal auto-correlation, thus, unlike in Bulla et al. (2014), was not controlled for the length of previous (partner's) incubation bout. Model with random intercept of nest ID nested within pair ID did not converge.

Table S10

Mixed model estimates of incubation bout length (mean-centered within individual) in relation to period (first or second 48 h of the experiment) and whether the incubation bout was treated (insulated); model was controlled for sex, and quadratic day of incubation.

Fixed effects on bout length (min)	Relative to	Estimate	95% CI	*z*	*P*
(Intercept)		-14.2	(-55.3, 26.8)		
Sex (male)	female	1.6	(-38.9, 42)	0.1	1
Day of incubation (1st polynomial)		-26.9	(-238.7, 184.9)	-0.33	1
Day of incubation (2nd polynomial)		-118.3	(-312.4, 75.7)	-1.59	0.47
Period (2nd)	1st	21.9	(-23.1, 66.9)	1.27	0.70
Incubation bout (treated)	untreated	6.2	(-34.3, 46.7)	0.4	1
**Random effects**		**Variance**			
Nest (Intercept)		0			
Day of incubation (1st polynomial)		109			
Day of incubation (2nd polynomial)		2313			
Residual		5142			

N = 87 incubation bouts from 14 nests from 7 nest-pairs; 42 treated bouts, 45 untreated bouts. The model lacked major temporal auto-correlation, thus, unlike in Bulla et al. (2014), was not controlled for the length of the previous (partner's) incubation bout. Model with random intercept of nest ID nested within pair ID did not converge.

Table S11

Mixed model estimates of energy provided to the nest-scrape (transformed[a]) with treatment (heated egg, polystyrene insulation, or control).

Fixed effects on provided energy (mW)	Relative to	Estimate	95% CI	z	P
(Intercept)		-241	(-384, -97)		
Treatment (control)	heated egg	233	(104, 361)	4.25	<0.0001
Treatment (insulation))	heated egg	158	(29, 287)	2.89	0.011
**Random effects**		**Variance**			
Nest (Intercept)		0			
Day of experiment (Intercept)		4415			
Residual		7482			

N = 15 fifty-minute measurements acquired during 2 days with 3 repeated measurement from 3 nest scrapes (i.e., treatment was rotated among the nest-scrapes); 3 missing measurements due to system failure at one nest scrape during day one

[a] Provided energy was transformed within nest scrape and day of experiment by subtracting, for each nest scrape and day of the experiment, the control value from all other values.

7. ESTIMATING ENERGETIC DEMANDS OF INCUBATION

Precise estimates of energetic demands of incubation (i.e., costs of keeping eggs at incubation temperature) are scarce. Probably the most precise estimates for average demands of incubation were derived from the difference in oxygen consumption of incubating and non-incubating birds: for 11.6 g zebra finches, *Taenopygia guttata*, this was about 84 mW at 10°C (i.e., 7.3 mW/g or 305 W/h; estimated from equations in Vleck 1981); for 75 g starlings, *Sturnus vulgaris*, about 188 mW at 10°C (2.5 mW/g or 675 W/h; estimated from equations in Biebach 1979). Both estimates are about 20% of the resting metabolic rate. Although similar measurements are absent for semipalmated sandpipers, its energetic demands of incubation can be estimated either from Vleck‘s zebra finch and Biebach’s starling equations or as 20% of semipalmated sandpiper‘s resting metabolic rate. In either case the estimate is body mass and temperature dependent.

Our estimates (Table S12) are based on the average body mass of semipalmated sandpipers in Barrow, which is about 27 g (associated data in Dryad: Bulla et al. 2013; Bulla et al. 2014), and temperatures of 10°C (as in zebra finch and starling study) and 6.2°C (median tundra temperature in Barrow).

Table S12

Estimated energetic demands of keeping eggs at incubation temperature for a 27 g semipalmated sandpiper.

Temperature [°C]	Cost [mW]	Cost [mW/g]	Assumption	From
10.0	197	7.31	cost for zebra finch	(Vleck 1981)
6.2	215	7.95	cost for zebra finch	(Vleck 1981)
10.0	68	2.50	cost for starling	(Biebach 1979)
6.2	90	3.34	cost for starling	(Biebach 1979)
10.0	261	9.67	resting metabolic rate of 1305 mW*	(Norton 1973)
6.2	284	10.50	resting metabolic rate of 1422 mW*	(Norton 1973)

*Assuming that 1 liter of O2 = 20.1 kJ

8. REFERENCES

Ashkenazie S, Safriel UN. 1979. Breeding Cycle and Behavior of the Semipalmated Sandpiper at Barrow, Alaska. Auk. 96:56-67.

Biebach H. 1979. Energetik des Brütens beim Star (*Sturnus vulgaris*). J Ornithol. 120:121-138.

Bulla M, Valcu M, Rutten AL, Kempenaers B. 2013. Data from: Biparental incubation patterns in a high-Arctic breeding shorebird: how do pairs divide their duties? : Dryad Data Repository. Available from: http://dx.doi.org/10.5061/dryad.nh8f0.

Bulla M, Valcu M, Rutten AL, Kempenaers B. 2014. Biparental incubation patterns in a high-Arctic breeding shorebird: how do pairs divide their duties? Behav Ecol. 25:152-164.

Cohen J. 1988. Statistical power analysis for the behavioral sciences, 2nd ed. Hillsdale, New Jersey: Lawerence Erlbaum.

Cresswell W, Holt S, Reid JM, Whitfield DP, Mellanby RJ. 2003. Do energetic demands constrain incubation scheduling in a biparental species? Behav Ecol. 14:97-102.

Díaz-Uriarte R. 2002. Incorrect analysis of crossover trials in animal behaviour research. Anim Behav. 63:815-822.

Hills M, Armitage P. 1979. The two-period cross-over clinical trial. British Journal of Clinical Pharmacology. 58:S703-S716.

Jones B, Kenward MG. 1989. Design and analysis of cross over trials. New York: Chapman and Hall.

Norton D. 1973. Ecological Energetics of Calidridine Sandpipers Breeding in Northern Alaska. Fairbanks: University of Alaska, p. 195.

Vleck CM. 1981. Energetic Cost of Incubation in the Zebra Finch. Condor. 83:229-237.
