# Supplementary material for: Biparental incubation-scheduling: no experimental evidence for major energetic constraints
Source: Behav Ecol. 2014 Sep 3;26(1):30–7. doi: 10.1093/beheco/aru156 (PMC4309980; doi:10.1093/beheco/aru156)
Supplement: Supplementary Data [file supp_aru156_Explanation_of_supplementary_material.doc]

Supplementary Material and data

Supplementary material, <http://www.beheco.oxfordjournals.org/>.

**Supplementary 1** contains details regarding: 1. Reanalysis of the Insulation Experiment, 2. Seasonal Differences, 3. Artificial Experiment, 4. A Priori Power Analyses, 5. Sample Sizes, 6. Models, 7. Estimating Energetic Demands of Incubation, and 8. References.

**Supplementary 2** contains figures that depict all data from heated egg experiment for a given nest.

**Supplementary 3** contains figures that depict all data from nest insulation experiment for a given nest.

**Data** are available from figshare.com digital repository at http://figshare.com/articles/Data_from_Biparental_incubation_scheduling_no_experimental_evidence_for_major_energetic_constraints_/1035052

**R-script** (of the statistical analyses, figures, power analyses, etc.) is available from figshare.com digital repository at <http://figshare.com/articles/R_script_from_Biparental_incubation_scheduling_no_experimental_evidence_for_major_energetic_constraints/1035048>
